# Green synthesis of new chiral 1-(arylamino)imidazo[2,1-*a*]isoindole-2,5-diones from the corresponding α-amino acid arylhydrazides in aqueous medium

**DOI:** 10.3762/bjoc.14.271

**Published:** 2018-11-26

**Authors:** Nadia Bouzayani, Jamil Kraїem, Sylvain Marque, Yakdhane Kacem, Abel Carlin-Sinclair, Jérôme Marrot, Béchir Ben Hassine

**Affiliations:** 1 Laboratoire de Synthèse Organique Asymétrique et Catalyse Homogène, (UR 11ES56) Université de Monastir, Faculté des Sciences de Monastir, avenue de l’environnement, 5000 Monastir, Tunisie. Fax: (+216) 73 500 278; Tel: (+216) 73; 2 Laboratoire de Développement Chimique, Galénique et Pharmacologique des Médicaments, Faculté de Pharmacie de Monastir, Université de Monastir, Rue Avicenne, 5000 Monastir, Tunisie; 3 Université de Versailles Saint-Quentin-en-Yvelines, Institut Lavoisier de Versailles (ILV), UMR CNRS 8180, 45 avenue des Etats-Unis, 78 035 Versailles Cedex, France; 4 Université de Versailles Saint-Quentin-en-Yvelines, Département de chimie, 45 avenue des Etats-Unis, 78 035 Versailles Cedex, France

**Keywords:** sodium dodecyl sulfate, totally diastereoselective, *trans*-stereochemistry

## Abstract

New chiral 1-(arylamino)imidazo[2,1-*a*]isoindole-2,5-dione derivatives were obtained in good to excellent yields via the cyclocondensation of 2-formylbenzoic acid and various α-amino acid arylhydrazides using water as the solvent in the presence of sodium dodecyl sulfate as the surfactant and under simple and minimum manipulation, without purification. The reaction is totally diastereoselective and gives access to the nitrogenated tricyclic core with a relative *trans* stereochemistry.

## Introduction

**Figure 1:**
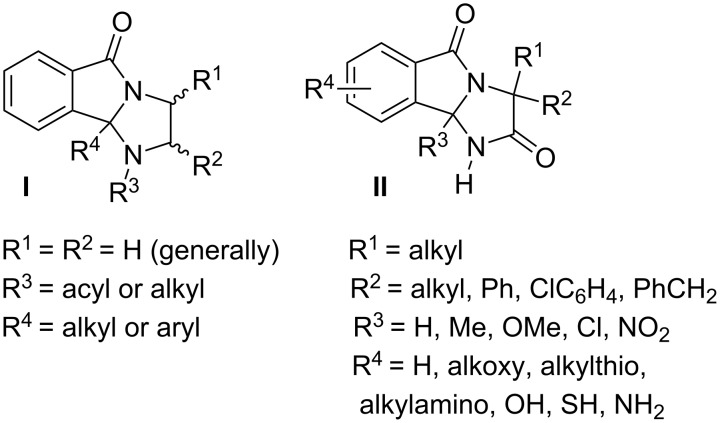
Chemical structures of analogues.

Tricyclic compounds, such as imidazo[2,1-*a*]isoindol-5-ones, are widely distributed in nature and possess several biological activities. In 1967, Geigy described the preparation of 1,2,3,9*b*-tetrahydro-5*H*-imidazo[2,1-*a*]isoindol-5-ones **I** which are recognized as anti-inflammatory and analgesic agents [1]. Other compounds, such as 1*H*-imidazo[2,1-*a*]isoindole-2,5-diones **II** have been known as an important herbicides for bud growth inhibition [2–6] and plant growth regulation [7–11]. Several research groups have turned to the synthesis of these structural analogues (Figure 1) [12]. For example, the stereoselective synthesis of chiral 1*H*-imidazo[2,1-*a*]isoindole-2,5(3*H*,9b*H*)-diones [6] and 1,2,3,9b-tetrahydro-5*H*-imidazo[2,1-*a*]isoindol-5-ones [13] has been described by Katritzky et al. using α-aminoamides and diamines, respectively.

**Scheme 1:**
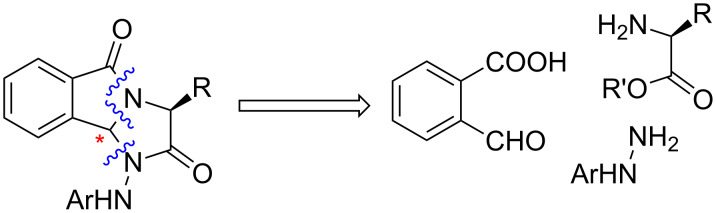
Strategy for the formation of 1-(arylamino)-1*H*-imidazo[2,1-*a*]isoindole-2,5(3*H*,9b*H*)-diones.

Also, the imidazo[2,1-*a*]isoindole-2,5-dione derivatives of primaquin have been synthesized from α-amino acids [14]. Focusing on the 1*H*-imidazo[2,1-*a*]isoindolone skeleton, Hosseini-Zare et al. reported the synthesis of new 2,3-diaryl-5*H*-imidazo[2,1-*a*]isoindol-5-ones via the one pot reaction of 1,2-diketones, 2-formylbenzoic acid and ammonium acetate [15]. These methodologies usually use toxic solvents such as benzene [16], dichloromethane and some catalyst such as *para*-toluenesulfonic acid [17]. Furthermore, most of them induce to manage undesirable waste produced in stoichiometric amounts. Recently, such synthetic methods utilizing hazardous solvents and reagents and generating toxic waste have become discouraged and there have been many efforts to develop safer and environmentally benign alternatives. The green chemistry concept emerged in 1990 [18] with the aim at developing cleaner approaches through the simplification of chemical processing along with the decreases of waste and cost. As a part of our ongoing efforts directed toward the development of environmentally safe conditions for the synthesis of heterocyclic compounds starting from natural (L)-α-amino acids [19–23] and the reactivity of α-amino acid phenylhydrazides [24–25], we now report a green and eco-friendly procedure for the synthesis of new chiral 1-(arylamino)-1*H*-imidazo[2,1-*a*]isoindole-2,5(3*H*,9b*H*)-diones (Scheme 1).

## Results and Discussion

### Synthesis

**Scheme 2:**
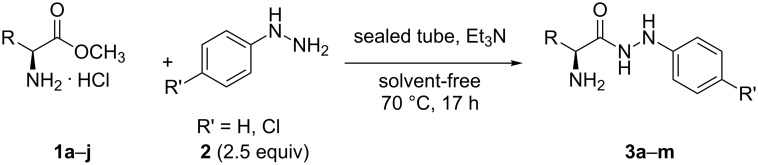
Synthesis of the starting (L)-α-amino acid phenylhydrazides and 4-chlorophenylhydrazides **3a**–**m** under solvent-free conditions.

The starting (L)-α-amino acid arylhydrazides **3** were prepared in a manner similar to the well-known procedures [26–28], improving the amount of the added arylhydrazine **2** down to 2.5 equiv (Scheme 2, Table 1) in a sealed tube in the presence of Et_3_N and under solvent-free conditions. From green chemistry point of view, this enhanced procedure appears to be a good alternative of the precedents for the synthesis of hydrazides **3a**–**m** [26–29]. Moreover, the phenylglycine phenylhydrazide (**3d**), (L)-cysteine phenylhydrazide (**3g**), (L)-tyrosine phenylhydrazide (**3j**), (L)-alanine 4-chlorophenylhydrazide (**3k**), (L)-phenylglycine 4-chlorophenylhydrazide (**3l**) and (L)-phenylalanine 4-chlorophenylhydrazide (**3m**) were synthesized for the first time in this work.

**Table 1:** Synthesis of the α-amino acid arylhydrazides **3a**–**m**.

entry	product (mol %)	R	R’	yield^a^ [%]	mp [°C]

1	**3a**	Me	H	87	122–124
2	**3b**	iPr	H	80	138–140
3	**3c**	iBu	H	70	152–154
4	**3d**^b^	Ph	H	83	138–140
5	**3e**	Bn	H	93	142–144
6	**3f**	(CH_2_)_2_SMe	H	81	128–130
7	**3g**	CH_2_SH	H	71	98–100
8	**3h**	CH_2_OH	H	71	163–165
9	**3i**	3-CH_2_-1*H*-indole	H	80	168–170
10	**3j**	CH_2_C_6_H_4_OH	H	77	134–136
11	**3k**	Me	Cl	69	128–130
12	**3l**	Ph	Cl	70	135–137
13	**3m**	Bn	Cl	74	140–142

^a^Yield of the isolated product. ^b^The compound **3d** was obtained as a racemic mixture.

**Scheme 3:**
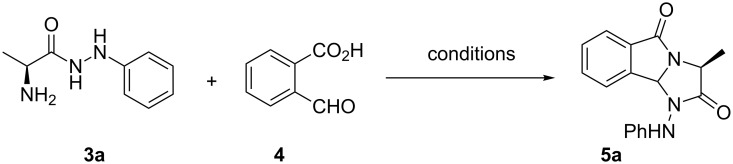
Cyclocondensation of 2-formylbenzoic acid (**4**) with (L)-alanine phenylhydrazide (**3a**).

Verardo et al. [29] have shown that refluxing α-amino acid phenylhydrazides with levulinic acid in toluene led to the corresponding dihydro-1*H*-pyrrolo[1,2-*a*]imidazole-2,5-diones. These authors explained clearly the behavior of the solvent and its influence in the reaction. In addition, substituted 1-hydroxy-1*H*-imidazo[2,1-*a*]isoindole-2,5(3*H*,9b*H*)-diones have been synthesized by the condensation of (L)-α-aminohydroxamic acids and 2-formylbenzoic acid under the same conditions [30]. Thus, they choose toluene as the best solvent for this reaction. To prepare the new chiral 1-(arylamino)imidazo[2,1-*a*]isoindole-2,5-diones **5a**–**m** under greener conditions, we investigated the reaction in nontoxic solvents such as water and dimethyl carbonate (DMC). Indeed, DMC is well-known as safe reagent and solvent that has been used for many green applications [31–33]. On the other hand, water is a simply and environmentally benign solvent and interest has increasingly turned to it as the greenest solvent of chemical reactions [34]. In order to find the best reaction conditions, a model reaction was chosen using (L)-alanine phenylhydrazide (**3a**, Scheme 3), and the results of these optimized studies were summarized in Table 2.

**Table 2:** Optimization of the reaction conditions for the cyclocondensation of 2-formylbenzoic acid (**4**) with (L)-alanine phenylhydrazide (**3a**).^a^

entry	additive [mol %]	time [h]	conditions^a^	yields [%]

1	–	4	DMC^b^	82
2	–	10	H_2_O	72
3	SDS [5]	10	H_2_O	77
4	SDS [10]	10	H_2_O^c^	90
5	–	10	neat^d^	89
6	–	4	toluene^e^	88

^a^General conditions for all the six entries: (L)-alanine phenylhydrazide (0.66 mmol), 2-formylbenzoic acid (0.66 mmol), 120 °C. ^b^Conditions: DMC (1.5 mL) in sealed tube. ^c^Conditions: water (1.5 mL) and sodium dodecyl sulfate (SDS) in sealed tube. ^d^Conditions: in sealed tube. ^e^Conditions: toluene (3 mL), under argon.

**Scheme 4:**
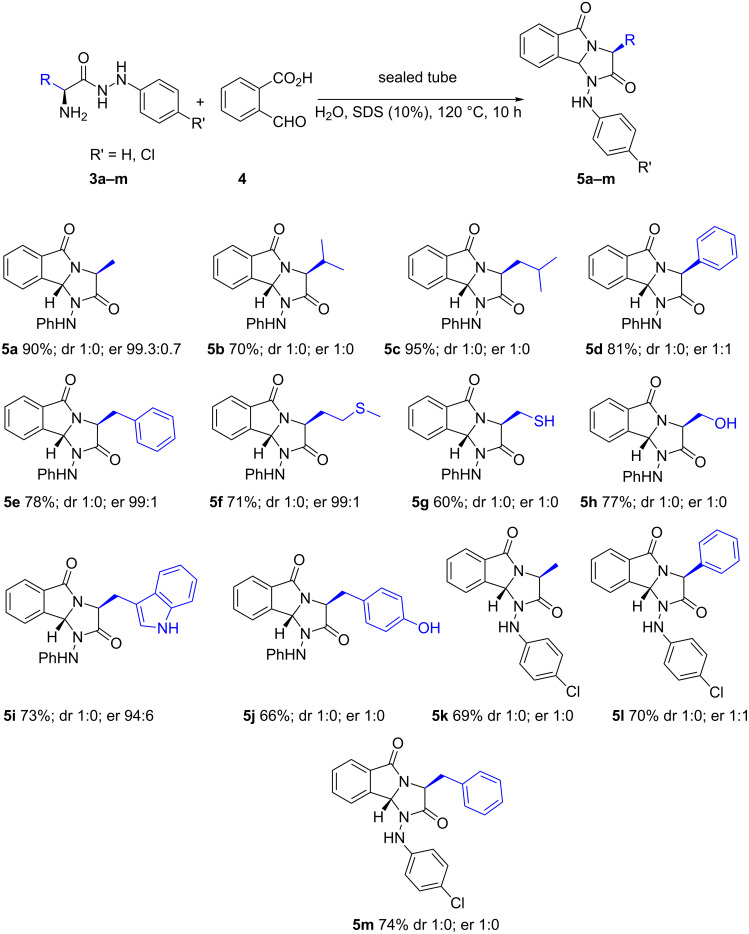
Synthesis of the nitrogenated tricyclic compounds **5a**–**m**. Diastereoisomeric (dr) and enantiomeric (er) ratio were determined by chiral HPLC (see Supporting Information File 1).

The best result was obtained when a mixture of hydrazide **3a** and formylbenzoic acid **4** was heated at 120 °C (oil bath) using water as the solvent in the presence of SDS (10%) as the surfactant for 10 h leading to a 90% yield in **5a**. To demonstrate the generality of this method, we next investigated the scope of this reaction under the optimized conditions. A variety of the 1-(arylamino)imidazo[2,1-*a*]isoindole-2,5-diones **5b**–**m** were prepared and the obtained results are summarized in Scheme 4. The mild conditions of the reaction gave access to the tricyclic compounds **5a**–**m** providing similar yields, regardless the amino acid residue is. A weak acidic function was well-tolerated yielding **5j** with 66%. Bulky groups did not roughly affect the efficiency of the reactivity (**5b**, **5c**).

The new approach described in this work for the preparation of compounds **5a**–**m** has the following advantages: (i) notably, under the reaction conditions described above, we have never observed the formation of degradation products. (ii) The reaction was performed in water, in the presence of SDS (sodium dodecyl sulfate) as the surfactant. Based on previous studies [35], the most effective method for ensuring the solubility of reactants in water is the use of surfactants that can form micelles with a hydrophobic core and a hydrophilic corona. (iii) This clean method afforded compounds **5a**–**m** without the need of additional purification such as column chromatography or recrystallization. (iv) The use of a sealed tube [36–43] obeys to four out of the twelve green chemistry principles: a cleaner and eco-friendly reaction profile with minimum of waste, solvent without negative environmental impact, shorter reaction time and safely work with a pressure tube canning prevent fires and emissions of hazardous compounds and toxic gas. (v) The reaction described in this work occurred with high atom efficiency (atom economy of 90%) and produced only stoichiometric H_2_O as waste (E-factor of 0.1).

### Stereochemistry and mechanism

**Figure 2:**
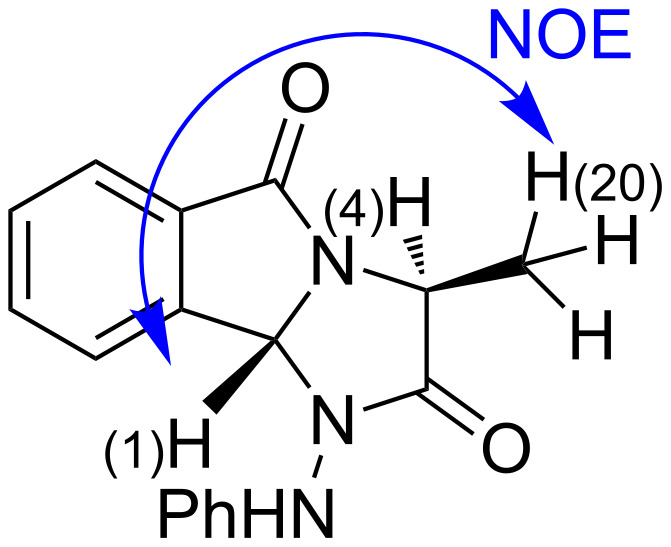
NOEs correlation showing the stereochemistry of the compound **5a**.

The stereochemistries of **5a**–**m** were ascribed by NOE NMR experiments. By example using **5a** depicted in Figure 2, the ^1^H NMR spectrum showed that H(1) appears at 5.95 ppm as a singlet and H(4) at 4.72 ppm as a quadruplet. No distinct NOE effect was observed between H(1) and H(4), when either H(1) or H(4) was irradiated. A significant positive NOE effect was observed between H(1) and H(20). Thus, NOE analysis assumed that H(1) is in a *trans*-orientation with H(4).

**Figure 3:**
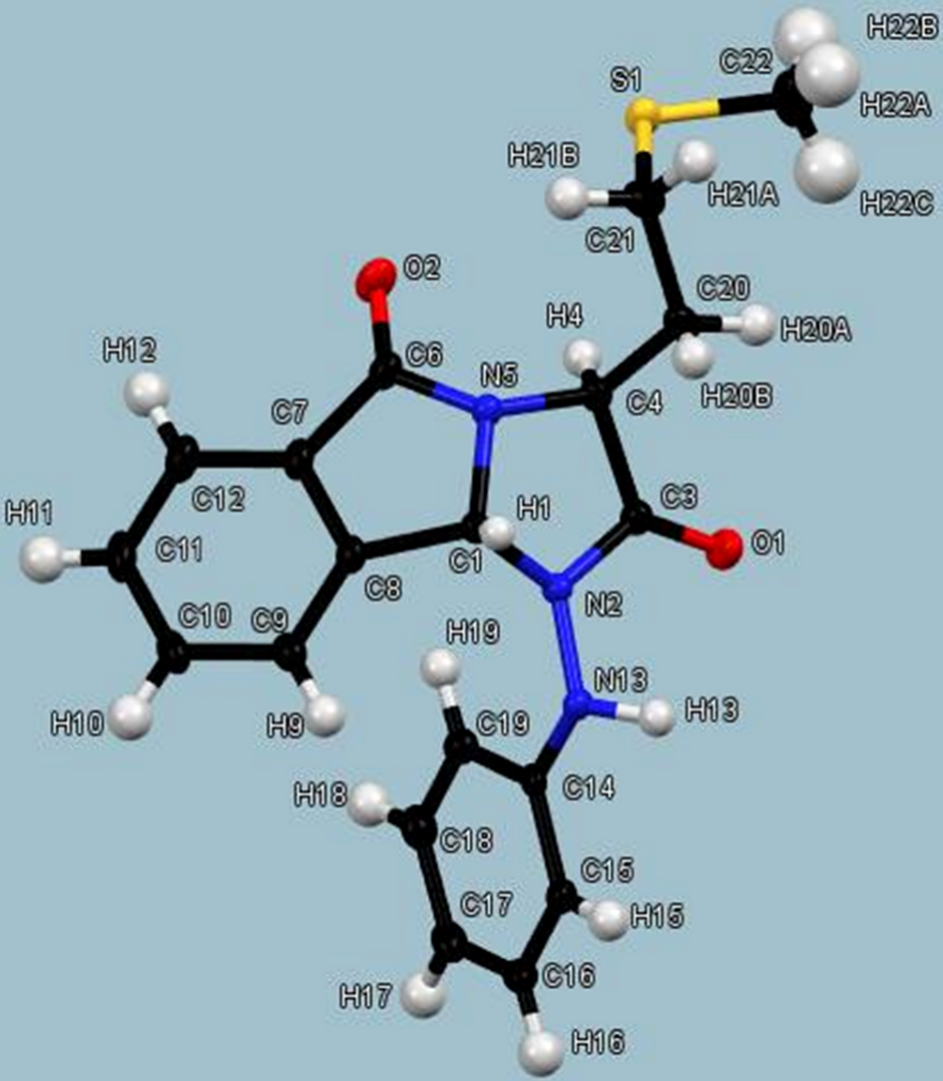
X-ray crystal structure of **5f** shown at the 30% probability level.

In addition, various crystallization tests were carried out in order to confirm the absolute stereochemistry by X-ray diffraction. The chiral 3-(2-(methylthio)ethyl)-1-(phenylamino)-1*H*-imidazo[2,1-*a*]isoindole-2,5(3*H*,9b*H*)dione (**5f**) crystallized [44–49] using a mixture of CH_2_Cl_2_/diethyl ether (3:1) as a single diastereoisomer with the (3*S*,9*R*) configuration (Figure 3) showing clearly the *trans* relative stereochemistry.

The dihedral angle value of −163.1° for H(1)–C(1)–C(4)–H(4) close to the anti-planarity corroborates the expected non correlation between H(1) and H(4) in the NOE experiment. The structure contains two carbonyl moieties C(6)–O(2) and C(3)–O(1) with similar distances of 1.222 and 1.218 Å, respectively. The intramolecular hydrogen bond O(1)–H(13) remains strong in the nitrogenated tricycle (2.579 Å) vis-à-vis of the starting hydrazide.

**Scheme 5:**
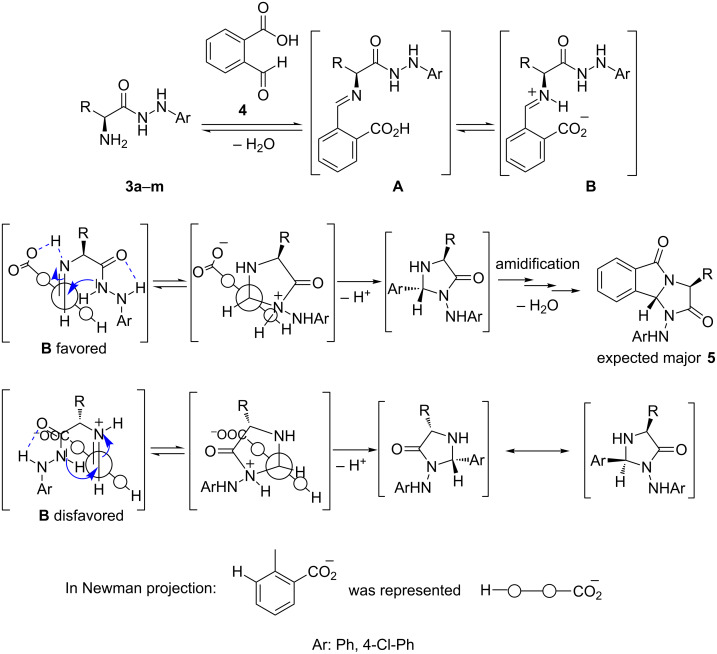
Proposed partial mechanism with a selectivity model.

Analyzing the mechanism of the reaction between the 2-formylbenzoic acid **4** and α-amino acid arylhydrazides **3**, we assumed that the first steps lead to the formation of the imine intermediate **A**, which is the result of the nucleophile addition of the α-amino group on the most electrophilic center in **4**. This imino-carboxylic acid form likely evolves to its iminium-carboxylate form **B** [50]. This last intermediate involves the existence of two possible transition states for the cyclization of the imidazolidinone core of which one of them is favored by an intramolecular hydrogen bond (Scheme 5). This control is the main difference with the known reaction [51] between the hydrazides **3** and the phthalaldehydes in which two diasteroisomers have been observed. If the amino acid residue (R on the Scheme 5) enough implicates steric hindrance with the carboxyaromatic part of **B**, the pathway with disfavored transition state becomes unlikely and then the selectivity is important. Our model is in agreement with the diasteroisomeric ratio results. In fact, a total diastereoselectivity was observed, including **5d** (R = Ph) since an epimerization of the initial asymmetric center was observed, therefore racemic *trans*-isomers were obtained in this case. Interestingly, acidic (phenolic **5j**) and basic moieties (as indolyl **5i**) on the amino acid residue did not affect the selectivity.

## Conclusion

In summary, we have developed environmentally safe conditions for the synthesis of new chiral 1-(arylamino)-1*H*-imidazo[2,1-*a*]isoindole-2,5(3*H*,9b*H*)-diones in good yields using water as the solvent in sealed tube. The aspects of greenness and good results make this methodology a practical and atom economical alternative in the whole of the processing since the syntheses of the α-amino acid arylhydrazide precursors behave this greener aspect. Once more, the processing includes this eco-friendly way up to have the compounds in hand since simple precipitation allows to isolate the nitrogenated tricyclic compounds; no purification is needed. The model for the key step of the cyclization clearly explains the stereochemistries, which are observed with a total *trans*-diastereoselectivity controlled by intramolecular hydrogen bonds.

## Supporting Information

File 1 Experimental procedures, spectroscopic and analytical data and copies of spectra of the products.

File 2 Crystallographic information for compound **5f**.

File 3 HPLC analysis of the products.
