# Supplementary material for: Green synthesis of new chiral 1-(arylamino)imidazo[2,1-a]isoindole-2,5-diones from the corresponding α-amino acid arylhydrazides in aqueous medium
Source: Beilstein J Org Chem. 2018 Nov 26;14:2923–30. doi: 10.3762/bjoc.14.271 (PMC6278760; doi:10.3762/bjoc.14.271)
Supplement: File 1 — Experimental procedures, spectroscopic and analytical data and copies of spectra of the products. [file Beilstein_J_Org_Chem-14-2923-s001.pdf]

BEILSTEIN JOURNAL OF ORGANIC CHEMISTRY

Supporting Information

for

Green synthesis of new chiral 1-(arylamino)imidazo[2,1-*a*]isoindole-2,5-diones from the corresponding α-amino acid arylhydrazides in aqueous medium

Nadia Bouzayani, Jamil Kraïem, Sylvain Marque, Yakdhane Kacem, Abel Carlin-Sinclair, Jérôme Marrot and Béchir Ben Hassine

Beilstein J. Org. Chem. **2018,** *14,* 2923–2930. doi:10.3762/bjoc.14.271

Experimental procedures, spectroscopic and analytical data and copies of spectra of the products

License and Terms: This is a supporting information file under the terms of the Creative Commons Attribution License (http://creativecommons.org/licenses/by/4.0). Please note that the reuse, redistribution and reproduction in particular requires that the authors and source are credited.
The license is subject to the *Beilstein Journal of Organic Chemistry* terms and conditions: (https://www.beilstein-journals.org/bjoc)

Table of contents

1. General methods……………………………………………………..……..………..…S2

2. General procedures…………………………………………..………..………S2

2.1. Synthesis of α-amino acid arylhydrazides **3a–m**……..…………………...……S2

2.2. General procedure for the synthesis of the 1-(arylamino)-1*H*-imidazo[2,1-*a*]isoindole-2,5(3*H*,9b*H*)-diones **5a–m** in water……..……………………..…....S4

3. ^{1}H NMR, ^{13}C NMR, DEPT 135, COSY NMR, HSQC NMR, HMBC NMR, NOESY NMR and FT-IR spectra of the products **5a–m**……..……………..…………..…………..S9

1. General methods

All reagents and chemicals were purchased from Sigma-Aldrich chemical company and Acros Organic. Solvents used in reactions were dried and distilled before use. Toluene was distilled over sodium metal. Reactions were monitored by thin layer chromatography (TLC) of aliquots using Merck 60 F-254 silica gel plates (0.25 mm layered thickness). Melting points were determined on a Büchi 510 capillary apparatus. NMR spectra were recorded on a Bruker AC-300 spectrometer [300 MHz (^{1}H) and 75 MHz (^{13}C)]. NMR spectra were calibrated on the non-fully deuteried residual solvent signal (ppm): in $CDCl_3$ at 7.26 (proton) and 77.16 (carbon), in C_6D_6 at 7.16 (proton) and 128.06 (carbon). IR spectra were recorded on a Nicolet 6700 FT-IR, ATR, to support with Diamond accuracy 1 cm^{-1}. Electrospray ionisation (ESI) mass spectrometry data were recorded on an UPLC Waters device (in positive mode); for the voltages of the mass spectrometries, the following abbreviations are used: C Capillary (kV), SC Sampling Cone, EC Extraction Cone. Calibration was performed with sodium formate (range from 100 to 1000 $g \cdot mol^{-1}$) and the lockspray (lockmass on the leucine encephaline 556.2771 $g \cdot mol^{-1}$) was used without collision energy; the relative intensity of peaks is given in brackets. Optical rotations were measured by using a Perkin Elmer Polarimeter (Model 341) using a mercury lamp (578 nm).

2. General procedures

2.1. Synthesis of α-amino acid arylhydrazides 3a–m

Freshly distilled arylhydrazine (25 mmol, 2.5 equiv) and (L)-α-amino acid methyl ester hydrochloride (10 mmol, 1 equiv) were mixed in sealed tube well closed in the presence of Et_3N (1 equiv). The mixture was heated at 70 °C for 17 h and then diluted

with EtOAc (10 mL), washed with water (3 mL), and dried over $MgSO_4$. After the evaporation of the solvent in vacuo, Et_2O (10 mL) was added to precipitate the products **3a–m** which were obtained, after filtration, as solid compounds.

(L)-Phenylglycine phenylhydrazide (3d): Yield 83%. white solid; mp 138–140 °C. FT-IR (neat, cm^{-1}): 3351, 3227, 3057, 1660, 1590; ^1H NMR (300 MHz, $CDCl_3$): δ= 1.91 (s, 1H), 4.63 (s, 1H), 6.08 (s, 2H), 6.70–7.19 (m, 5H), 7.34–7.44 (m, 5H), 8.45 (s, 1H); ^{13}C NMR (75 MHz, $CDCl_3$): δ= 60.11, 113.21, 122.43, 127.23, 128.98, 129.15, 129.78, 133.36, 149.65, 171.09 ppm; ESI(+)-MS CH_3CN [C= 2, SC= 20, EC= 2]: HRMS ES+ for $C_{14}H_{15}N_3O$ m/z: $[M+H]^+$ Calc. 242.1244, found: 242.1234.

(L)-Cysteine phenylhydrazide (3g): Yield 71%. white solid; mp 98–100 °C; $[\alpha]_{578}^{25}$= +52.0 ± 2.22 (MeOH, C= 0.59). FT-IR (neat, cm^{-1}): 3214, 3027, 1655, 1601, 1495; ^1H NMR (300 MHz, $CDCl_3$): δ= 1.72 (s, 1H), 2.75 (dd, 1H, ^2J= 13.8 Hz, ^3J= 3.9 Hz), 3.14 (dd, 1H, ^2J= 13.8 Hz, ^3J= 5.4 Hz), 3.67 (dd, 1H, ^3J= 3.9 Hz, ^3J= 5.4 Hz), 5.18 (s, 1H), 6.10 (s, 2H), 7.18–7.27 (m, 5H), 9.01 (s, 1H); ^{13}C NMR (75 MHz, $CDCl_3$): δ= 30.11, 59.23, 113.01, 121.45, 129.32, 149.14, 171.02 ppm; ESI(+)-MS CH_3CN [C= 2, SC= 20, EC= 2]: HRMS ES+ for $C_9H_{13}N_3OS$ m/z: $[M+H]^+$ Calc. 212.0858, found: 212.0859.

(L)-Tyrosine phenylhydrazide (3j): Yield 77%. white solid; mp 134–136 °C; $[\alpha]_{578}^{25}$= +15.3 ± 0.3 (MeOH, C= 0.94). FT-IR (neat, cm^{-1}): 3372, 3351, 2903, 1692, 1599, 1253; ^1H NMR (300 MHz, $CDCl_3$): δ= 3.09 (dd, 1H, ^2J= 13.2 Hz, ^3J= 6.8 Hz), 3.10 (dd, 1H, ^2J= 13.2 Hz, ^3J= 6.1 Hz), 4.11 (dd, 1H, ^3J= 6.1 Hz, ^3J= 6.8 Hz), 5.09 (s, 1H), 6.11 (s, 1H), 6.70 (d, 2H, ^3J= 7.4 Hz), 6.88–6.95 (m, 5H), 7.13 (d, 2H, ^3J= 7.4 Hz), 8.02 (s, 1H); ^{13}C NMR (75 MHz, $CDCl_3$): δ= 40.12, 57.01, 113.22, 115.43, 121.76, 128.98, 129.43, 130.76, 150.01, 156.32, 172.34 ppm; ESI(+)-MS CH_3CN [C= 2, SC= 20, EC= 2]: HRMS ES+ for $C_{15}H_{17}N_3O_2$ m/z: $[M+H]^+$ Calc. 272.1337, found: 272.1332.

(L)-alanine 4-chlorophenylhydrazide (3k): Yield 69%. white solid; mp 128–130 °C; $[\alpha]_{578}^{25}$= +23.91 ± 0.69 (MeOH, C= 0.322 ± 0.004). FT-IR (neat, cm^{-1}): 3100, 2900, 1699, 1450; ^1H NMR (300 MHz, $CDCl_3$): δ= 1.42 (d, 3H, J= 6.7 Hz), 1.63 (s, 2H), 3.65 (q, 1H, J= 6.7 Hz), 6.24 (s, 1H), 6.76 (d, 2H, J= 8.5 Hz), 7.20 (d, 2H, J= 8.5 Hz), 8.97 (s, 1H); ^{13}C NMR (75 MHz, $CDCl_3$): δ= 21.76, 50.28, 114.78, 125.84, 129.09,

146.74, 175.39 ppm; ESI(+)-MS CH_3CN [C= 2, SC= 20, EC= 2]: HRMS ES+ for $C_9H_{12}ClN_3O$ m/z: $[M+H]^+$ Calc. 214.0673, found: 214.0688.

(L)-phenylglycine 4-chlorophenylhydrazide (3l): Yield 70%. white solid; mp 135–137 °C; $[\alpha]_{578}^{25}$= +43.51 ± 0.60 (MeOH, C= 0.562 ± 0.004). FT-IR (neat, cm^{-1}): 3261, 3190, 2997, 1689; ^1H NMR (300 MHz, $CDCl_3$): δ= 1.71 (s, 2H), 4.71 (s, 1H), 5.32 (s, 1H), 6.68 (d, 2H, *J*= 8.4 Hz), 7.16 (d, 2H, *J*= 8.4 Hz), 7.30–7.47 (m, 5H), 8.62 (s, 1H); ^{13}C NMR (75 MHz, $CDCl_3$): δ= 61.29, 113.33, 127.56, 128.21, 129.23, 129.36, 130.89, 133.87, 148.48, 172.15 ppm; ESI(+)-MS CH_3CN [C= 2, SC= 20, EC= 2]: HRMS ES+ for $C_{14}H_{15}ClN_3O$ m/z: $[M+Na]^+$ Calc. 298.0723, found: 298.0723.

(L)-phenylalanine 4-chlorophenylhydrazide (3m): Yield 74%. white solid; mp 140–142 °C; $[\alpha]_{578}^{25}$= +24.74 ± 0.30 (MeOH, C= 0.768 ± 0.004). FT-IR (neat, cm^{-1}): 3276, 3030, 2926, 1706; ^1H NMR (300 MHz, $CDCl_3$): δ= 1.60 (s, 2H), 2.92 (dd, 1H, *J*= 13.0 Hz, *J*= 7.3 Hz), 3.24–3.28 (m, 1H), 3.76–3.79 (m, 1H), 6.16 (s, 1H), 6.70 (d, 2H, *J*= 8.4 Hz), 7.20 (d, 2H, *J*= 8.4 Hz), 7.25 (d, 2H, *J*= 6.6 Hz), 7.33–7.40 (m, 3H), 8.86 (s, 1H); ^{13}C NMR (75 MHz, $CDCl_3$): δ= 33.76, 55.65, 114.82, 126.04, 127.13, 128.90, 129.07, 129.56, 137.18, 146.55, 173.83 ppm; ESI(+)-MS CH_3CN [C= 2, SC= 20, EC= 2]: HRMS ES+ for $C_{15}H_{16}ClN_3O$ m/z: $[M+H]^+$ Calc. 290.0923, found: 290.0911.

2.2. General procedure for the synthesis of the 1-(arylamino)-1*H*-imidazo[2,1-*a*]isoindole-2,5(3*H*,9b*H*)-diones 5a–m in water

A mixture of 2-formylbenzoic acid (100 mg, 0.66 mmol, 1 equiv) and α-amino acid arylhydrazide (0.66 mmol, 1 equiv) with catalytic amount of SDS (10%) in water (2 mL) in a closed sealed tube was heated at 120 °C (oil bath) for 10 h. The mixture was cooled at room temperature and extracted with EtOAc (3 × 5 mL). The organic phase was dried over $MgSO_4$ and concentrated in vacuo affording the compounds **5a–m** with good yields.

3-Methyl-1-(phenylamino)-1*H*-imidazo[2,1-*a*]isoindole-2,5(3*H*,9*bH*)-dione (5a): Yield 90%. white solid; mp 200–202 °C; R_f 0.23 (EtOAc/*c*-C_6H_{12} 40:60); $[\alpha]_{578}^{25}$= -17.1 ± 0.4 (MeOH, C= 0.532 ± 0.005). FT-IR (neat, cm^{-1}): 3242, 2938, 1702, 1600, 1489, 1403, 1367, 1222; ^1H NMR (300 MHz, $CDCl_3$): δ= 1.63 (d, 3H, *J*= 7.2 Hz), 4.72 (q, 1H, *J*= 7.2 Hz), 5.95 (s, 1H), 6.52 (d, 2H, *J*= 8.4 Hz), 6.88 (t, 1H, *J*= 7.2 Hz), 7.08–7.13 (m, 2H), 7.54–7.64 (m, 3H), 7.95 (d, 1H, *J*= 7.5 Hz); ^{13}C NMR (75 MHz, $CDCl_3$):

δ= 17.42, 53.68, 73.86, 113.91, 122.03, 124.76, 124.98, 129.19, 130.85, 132.22, 133.16, 142.36, 145.85, 173.31, 174.44 ppm; ESI(+)-MS CH_3CN [C= 2, SC= 20, EC= 2]: HRMS ES^+ for $C_{17}H_{16}N_3O_2$ *m/z*: $[M+H]^+$ Calc. 294.1237, found: 294.1230.

3-Isopropyl-1-(phenylamino)-1*H*-imidazo[2,1-*a*]isoindole-2,5(3*H*,9b*H*)-dione (5b): Yield 70%. white solid; mp 212–214 °C; R_f 0.64 (EtOAc/c-C_6H_{12} 40:60); $[\alpha]^{25}_{578}$= + 30.5 ± 0.3 ($CHCl_3$, C= 0.878 ± 0.004). FT-IR (neat, cm^{-1}): 3228, 2967, 1219, 1709, 1602, 1495, 1398, 1367; ^1H NMR (300 MHz, $CDCl_3$): δ= 1.11 (d, 3H, *J*= 6.6 Hz), 1.27 (d, 3H, *J*= 6.9 Hz), 1.68 (sbr, 1H), 2.40-2.50 (m, 1H), 4.48 (d, 1H, J= 4.2 Hz), 5.92 (s, 1H), 6.36 (s, 1H), 6.54 (d, 2H, *J*= 7.5 Hz), 6.88 (t, 1H, *J*= 7.5 Hz), 7.10 (t, 2H, *J*= 7.5 Hz), 7.52–7.56 (m, 2H), 7.58–7.65 (m, 1H), 7.98 (d, 1H, *J*= 7.2 Hz); ^{13}C NMR (75 MHz, $CDCl_3$): δ= 18.15, 19.75, 31.32, 63.91, 75.89, 114.24, 122.22, 124.82, 125.07, 129.27, 130.91, 132.39, 133.23, 142.99, 146.29, 173.82, 174.16 ppm; ESI(+)-MS CH_3CN [C= 2, SC= 20, EC= 2]: HRMS ES^+ for $C_{19}H_{20}N_3O_2$ *m/z*: $[M+H]^+$ Calc. 322.1555, found: 322.1546.

3-Isobutyl-1-(phenylamino)-1*H*-imidazo[2,1-*a*]isoindole-2,5(3*H*,9b*H*)-dione (5c): Yield 95%. white solid; mp 230–232 °C; R_f 0.6 (EtOAc/c-C_6H_{12} 40:60); $[\alpha]^{25}_{578}$= -2.0 ± 0.2 ($CHCl_3$,C= 1.016 ± 0.005). FT-IR (neat, cm^{-1}): 3212, 3024, 2956, 1702, 1601, 1494, 1386, 1368, 1213; ^1H NMR (300 MHz, $CDCl_3$): δ= 1.06 (d, 3H, *J*= 6.6 Hz), 1.17 (d, 3H, *J*= 6.6 Hz), 1.64–1.74 (m, 1H), 1.79–1.88 (m, 1H), 1.97–2.06 (m, 1H), 4.66 (dd, 1H, J_1= 11.4 Hz, J_2= 3.6 Hz), 5.92 (s, 1H), 6.53 (d, 2H, *J*= 8.7 Hz), 6.87 (t, 1H, *J*= 7.5 Hz), 7.07–7.13 (m, 2H), 7.50–7.63 (m, 3H), 7.94 (d, 1H, *J*= 7.5 Hz); ^{13}C NMR (75 MHz, $CDCl_3$): δ= 20.88, 22.75, 24.91, 39.49, 56.56, 73.49, 113.41, 121.50, 124.24, 124.40, 128.67, 130.31, 131.81, 132.56, 141.87, 145.38, 172.96, 173.78 ppm; ESI(+)-MS CH_3CN [C= 2, SC= 20, EC= 2]: HRMS ES+ for $C_{20}H_{21}N_3O_2$ m/z: $[M+H]^+$ Calc. 336.1707, found: 336.1699.

3-Phenyl-1-(phenylamino)-1*H*-imidazo[2,1-*a*]isoindole-2,5(3*H*,9b*H*)-dione (5d): Yield 79%. white solid; mp 217–219 °C; R_f 0.51 (EtOAc/c-C_6H_{12} 40:60). FT-IR (neat, cm^{-1}): 3286, 3062, 1708, 1602, 1497, 1391, 1361, 1302, 1215; ^1H NMR (300 MHz, $CDCl_3$): δ= 1.64 (s, 1H), 5.72 (s, 1H), 6.07 (s, 1H), 6.21 (s, 1H), 6.47 (d, 2H, *J*= 8.1 Hz), 6.86 (t, 1H, *J*= 14.7 Hz), 7.08 (t, 2H, *J*= 8.4 Hz), 7.34–7.45 (m, 3H), 7.56–7.68 (m, 5H), 8.02 (d, 1H, *J*= 7.5 Hz); ^{13}C NMR (75 MHz, $CDCl_3$): δ= 59.52, 73.75, 113.27, 121.47, 124.38, 124.69, 125.87, 127.98, 128.41, 128.70, 130.49, 131.47,

132.91, 134.16, 142.05, 145.16, 170.99, 172.91 ppm; ESI(+)-MS CH_3CN [C= 2, SC= 20, EC= 2]: HRMS ES+ for $C_{22}H_{17}N_3O_2$ m/z: $[M+H]^+$ Calc. 356.1394, found: 356.1386.

3-Benzyl-1-(phenylamino)-1*H*-imidazo[2,1-*a*]isoindole-2,5(3*H*,9b*H*)-dione (5e): Yield 78%. white solid; mp 210–212 °C; R_f 0.35 (EtOAc/c-C_6H_{12} 40:60); $[\alpha]^{25}_{578}$= -8.1 ± 0.3 ($CHCl_3$, C= 0.836 ± 0.004). FT-IR (neat, cm^{-1}): 3238, 3030, 1707, 1600, 1492, 1401, 1368, 1221; ^1H NMR (300 MHz, $CDCl_3$): δ= 1.62 (sbr, 1H), 3.32 (dd, 1H, *J*= 14.1 Hz, *J*= 4.8 Hz), 3.39 (dd, 1H, *J*= 14.1 Hz, *J*= 4.2 Hz), 4.94 (t, 1H, *J*= 4.2 Hz), 4.98 (s, 1H), 5.86 (s, 1H), 6.28 (d, 2H, *J*= 8.1 Hz), 6.83 (t, 1H, *J*= 7.2 Hz), 7.03 (t, 2H, *J*= 7.5 Hz), 7.31–7.35 (m, 6H), 7.47 (t, 1H, *J*= 7.5 Hz), 7.56 (t, 1H, *J*= 7.5 Hz), 7.93 (d, 1H, *J*= 7.5 Hz); ^{13}C NMR (75 MHz, $CDCl_3$): δ= 37.40, 59.36, 74.66, 113.76, 121.88, 124.62, 125.00, 127.56, 128.80, 129.15, 130.29, 130.76, 132.08, 133.15, 135.70, 142.63, 145.49, 172.94, 173.76 ppm; ESI(+)-MS CH_3CN [C= 2, SC= 20, EC= 2]: HRMS ES+ for $C_{23}H_{19}N_3O_2$ m/z: $[M+H]^+$ Calc. 370.1550, found: 370.1544.

3-(2-(Methylthio)ethyl)-1-(phenylamino)-1*H*-imidazo[2,1-*a*]isoindole-2,5(3*H*,9b*H*)-dione (5f): Yield 71%. white solid; mp 191–193 °C; R_f 0.34 (EtOAc/c-C_6H_{12} 40:60); $[\alpha]^{25}_{578}$= +33.6 ± 0.4 ($CHCl_3$, C= 0.724 ± 0.004). FT-IR (neat, cm^{-1}): 3238, 3017, 1711, 1602, 1494, 1394, 1363, 1214; ^1H NMR (300 MHz, $CDCl_3$): δ= 2.19 (s, 3H), 2.20-2.28 (m, 1H), 2.39–2.45 (m, 1H), 2.81–2.86 (m, 2H), 4.73 (dd, 1H, *J*= 7.2 Hz, *J*= 5.1 Hz), 5.96 (s, 1H), 6.56 (d, 2H, *J*= 7.8 Hz), 6.89 (t, 1H, *J*= 7.2 Hz), 7.12 (t, 2H, *J*= 7.5 Hz), 7.57–7.67 (m, 3H), 7.98 (d, 1H, *J*= 7.2 Hz); ^{13}C NMR (75 MHz, $CDCl_3$): δ= 15.21, 30.11, 30.43, 57.17, 75.21, 113.92, 121.91, 124.80, 124.95, 129.18, 130.86, 132.01, 132.35, 142.91, 146.13, 173.97, 174.24 ppm; ESI(+)-MS CH_3CN [C= 2, SC= 20, EC= 2]: HRMS ES+ for $C_{19}H_{20}N_3O_2S$ m/z: $[M+H]^+$ Calc. 354.1271, found: 354.1265.

3-(Mercaptomethyl)-1-(phenylamino)-1*H*-imidazo[2,1-*a*]isoindole-2,5(3*H*,9b*H*)-dione (5g): Yield 60%. white solid; mp 202–204 °C; R_f 0.27 (EtOAc/c-C_6H_{12} 40:60); $[\alpha]^{25}_{578}$= -348 ± 3 ($CHCl_3$, C= 0.664 ± 0.004). FT-IR (neat, cm^{-1}): 3318, 3270, 3055, 1703, 1666, 1602, 1479, 1345, 1323; ^1H NMR (300 MHz, $CDCl_3$): δ= 3.69 (dd, 1H, *J*= 12.00 Hz, *J*= 7.5 Hz), 3.80 (s, 1H), 4.02 (dd, 1H, *J*= 12.3 Hz, *J*= 7.5 Hz), 4.89 (t, 1H, *J*= 7.5 Hz), 6.00 (s, 1H), 6.87–6.95 (m, 3H), 7.21–7.30 (m, 2H), 7.50–7.60 (m, 2H), 7.64–7.69 (m, 1H), 7.64–7.69 (d, 1H, *J*= 7,5 Hz), 8.88 (s, 1H); ^{13}C NMR (75 MHz, $CDCl_3$): δ= 38.24, 57.65, 66.85, 113.12, 120.96, 123.10, 124.36, 128.78, 129.37,

130.06, 132.98, 143.47, 146.95, 168.57, 171.81 ppm; ESI(+)-MS CH_3CN [C= 2, SC= 20, EC= 2]: HRMS ES+ for $C_{17}H_{16}N_3O_2S$ m/z: $[M+H]^+$ Calc. 326.0958, found: 326.0950.

3-(Hydroxymethyl)-1-(phenylamino)-1*H*-imidazo[2,1-*a*]isoindole-2,5(3*H*,9b*H*)-dione (5h): Yield 77%. white solid; mp 201–203 °C; R_f 0.13 (EtOAc/c-C_6H_{12} 40:60); $[\alpha]_{578}^{25}$= -28 ± 2 ($CHCl_3$, C= 0.380 ± 0.022). FT-IR (neat, cm^{-1}): 3508, 3283, 2923, 1695, 1598, 1498, 1411, 1362; ^1H NMR(300 MHz, $CDCl_3$): δ= 1.67 (s, 1H), 4.20 (dd, 1H, *J*= 11.7 Hz, *J*= 5.4 Hz), 4.36 (dd, 1H, *J*= 11.7 Hz, *J*= 4.5 Hz), 4.70–4.72 (m, 1H), 6.11 (s, 1H), 6.44 (d, 2H, *J*= 8.1 Hz), 6.90 (t, 1H, *J*= 8.1 Hz), 7.14 (t, 2H, *J*= 7.8 Hz), 7.58–7.66 (m, 3H), 8.00 (d, 1H, *J*= 7.2 Hz); ^{13}C NMR (75 MHz, $CDCl_3$): δ= 60.24, 63.46, 75.80, 113.95, 121.92, 124.86, 125.15, 129.34, 130.94, 132.02, 133.49, 143.18, 145.78, 172.83, 174.10 ppm; ESI(+)-MS CH_3CN [C= 2, SC= 20, EC= 2]: HRMS ES+ for $C_{17}H_{16}N_3O_3$ m/z: $[M+H]^+$ Calc. 310.1186, found: 310.1182.

3-((1*H*-indol-3-yl)methyl)-1-(phenylamino)-1*H*-imidazo[2,1-*a*]isoindole-2,5(3*H*,9b*H*)dione (5i): Yield 73%. white solid; mp 185–187 °C; R_f 0.18 (EtOAc/c-C_6H_{12} 40:60); $[\alpha]_{578}^{25}$= +99 ± 1 (MeOH, C= 0.494 ± 0.005). FT-IR (neat, cm^{-1}): 3449, 3261, 3057, 1602, 1491, 1460, 1401, 1302, 1221; ^1H NMR (300 MHz, $CDCl_3$): δ= 1.72 (sbr, 1H), 3.51 (dd, 1H, *J*= 15.6 Hz, *J*= 4.5 Hz), 3.59 (dd, 1H, *J*= 14.7 Hz, *J*= 3.9 Hz), 4.97 (t, 1H, *J*= 4.2 Hz), 6.18 (d, 2H, *J*= 7.8 Hz), 6.79 (t, 1H, *J*= 7.2 Hz), 6.95–7.00 (m, 2H), 7.08–7.13 (m, 1H), 7.18–7.23 (m, 3H), 7.40–7.46 (m, 2H), 7.55 (t, 1H, *J*= 7.5 Hz), 7.72 (d, 1H, *J*= 7.8 Hz), 7.93 (d, 1H, *J*=7.5 Hz), 8.24 (s, 1H); ^{13}C NMR (75 MHz, $CDCl_3$): δ= 27.63, 59.29, 74.66, 110.12, 111.25, 113.68, 119.32, 120.04, 121.73, 122.45, 124.05, 124.53, 124.92, 127.52, 129.04, 130.61, 132.16, 132.96, 136.25, 142.77, 145.47, 173.59, 173.71 ppm; ESI(+)-MS CH_3CN [C= 2, SC= 20, EC= 2]: HRMS ES+ for $C_{25}H_{21}N_4O_2$ m/z: $[M+H]^+$ Calc. 409.1659, found: 409.1654.

3-(4-Hydroxybenzyl)-1-(phenylamino)-1*H*-imidazo[2,1-*a*]isoindole-2,5(3*H*,9b*H*)-dione (5j): Yield 66%. white solid; mp 222–224 °C; R_f 0.32 (EtOAc/c-C_6H_{12} 40:60); $[\alpha]_{578}^{25}$= -58.7 ± 0.6 (MeCN, C= 0.552 ± 0.004). FT-IR (neat, cm^{-1}): 3274, 3203, 2985, 1706, 1673, 1598, 1517, 1493, 1412, 1220; ^1H NMR (300 MHz, $CDCl_3$): δ= 3.25–3.37 (m, 2H), 4.94 (t, 1H, *J*= 4.2 Hz), 5.11 (s, 1H), 5.82 (sbr, 1H), 5.93 (s, 1H), 6.31 (d, 2H, *J*= 7.8 Hz), 6.82–6.88 (m, 3H), 7.08 (t, 2H, *J*= 8.1 Hz), 7.22 (d, 2H, *J*= 8.1 Hz), 7.37 (d, 1H, *J*= 7.5 Hz), 7.48–7.53 (m, 1H), 7.60 (t, 1H, *J*= 7.5 Hz), 7.97 (d, 1H, *J*= 7.5 Hz);

^{13}C NMR (75 MHz, $CDCl_3$ + some drops of CD_3CN): δ= 36.35, 59.42, 74.20, 113.39, 115.52, 116.48, 121.32, 124.56, 126.60, 128.91, 130.50, 131.09, 131.88, 132.94, 142.53, 145.44, 155.92, 172.65, 173.67 ppm; ESI(+)-MS CH_3CN [C= 2, SC= 20, EC= 2]: HRMS ES+ for $C_{23}H_{19}N_3O_3$ m/z: $[M+H]^+$ Calc. 386.1499, found: 386.1492.

1-(4-chlorophenylamino)-3-methyl-1*H*-imidazo[2,1-*a*]isoindole-2,5(3*H*,9b*H*)-dione (5k): Yield 69%. white solid; mp 214–216 °C; R_f 0.34 (EtOAc/*c*-C_6H_{12} 40:60); $[\alpha]^{25}_{578}$= -54.67 ± 0.28 (CH_3CN, C= 0.765 ± 0.004). FT-IR (neat, cm^{-1}): 3324, 3221, 2932, 1716, 1611, 1400, 1392, 1233; ^{1}H NMR (300 MHz, $CDCl_3$): δ= 1.52 (d, 3H, *J*= 7.2 Hz), 4.65 (q, 1H, *J*= 7.2 Hz), 5.84 (s, 1H), 6.30 (d, 2H, *J*= 8.7 Hz), 6.93 (d, 2H, *J*= 8.7 Hz), 7.44–7.57 (m, 3H), 7.88 (d, 1H, *J*= 7.4 Hz); ^{13}C NMR (75 MHz, $CDCl_3$): δ= 17.41, 53.67, 74.08, 115.07, 124.70, 125.08, 126.83, 129.09, 131.03, 132.12, 133.34, 142.19, 144.67, 173.29, 174.85 ppm; ESI(+)-MS CH_3CN [C= 2, SC= 20, EC= 2]: HRMS ES^+ for $C_{17}H_{15}ClN_3O_2$ *m/z*: $[M+H]^+$ Calc. 328.0853, found: 328.0850.

1-(4-chlorophenylamino)-3-phenyl-1*H*-imidazo[2,1-*a*]isoindole-2,5(3*H*,9b*H*)-dione (5l): Yield 70%. white solid; mp 221–223 °C; R_f 0.52 (EtOAc/*c*-C_6H_{12} 40:60); $[\alpha]^{25}_{578}$= +14.19 ± 2.27 (MeOH, C= 0.155 ± 0.020). FT-IR (neat, cm^{-1}): 3274, 2985, 1706, 1598, 1220; ^{1}H NMR (300 MHz, $CDCl_3$): δ= 5.78 (s, 1H), 6.08 (s, 1H), 6.42 (d, 2H, *J*= 8.7 Hz), 7.05 (d, 2H, *J*= 8.7 Hz), 7.41–7.49 (m, 3H), 7.63–7.74 (m, 5H), 8.07 (d, 1H, *J*= 7.3 Hz); ^{13}C NMR (75 MHz, $CDCl_3$): δ= 65.89, 74.35, 115.06, 124.56, 125.33, 126.35, 127.28, 128.98, 129.23, 129.98, 131.16, 133.55, 134.99, 142.37, 144.41, 171.63, 173.12 ppm; ESI(+)-MS CH_3CN [C= 2, SC= 20, EC= 2]: HRMS ES^+ for $C_{22}H_{17}ClN_3O_2$ *m/z*: $[M+H]^+$ Calc. 390.1139, found: 390.1132.

3-benzyl-1-(4-chlorophenylamino)-1H-imidazo[2,1-*a*]isoindole-2,5(3H,9bH)-dione (5m): Yield 68%. white solid; mp 225–227 °C; R_f 0.32 (EtOAc/*c*-C_6H_{12} 40:60); $[\alpha]^{25}_{578}$= -45.18 ± 1.59 (CH_3CN, C= 0.830 ± 0.028). FT-IR (neat, cm^{-1}): 3203, 2985, 1706, 1598, 1220; ^{1}H NMR (300 MHz, $CDCl_3$): δ= 3.25–3.37 (m, 2H), 4.94 (t, 1H, *J*= 4.2 Hz), 5.11 (s, 1H), 5.82 (s, 1H), 5.93 (s, 1H), 6.31 (d, 2H, *J*= 7.8 Hz), 6.82–6.88 (m, 3H), 7.08 (t, 2H, *J*= 8.1 Hz), 7.22 (d, 2H, *J*= 8.1 Hz), 7.37 (d, 1H, *J*= 7.5 Hz), 7.48–7.53 (m, 1H), 7.60 (t, 1H, *J*= 7.5 Hz), 7.97 (d, 1H, *J*= 7.5 Hz); ^{13}C NMR (75 MHz, $CDCl_3$): δ= 36.35, 59.42, 74.20, 113.39, 115.52, 116.48, 121.32, 124.56, 126.60, 128.91, 130.50, 131.09, 131.88, 132.94, 142.53, 145.44, 155.92, 172.65, 173.67 ppm;

ESI(+)-MS CH_3CN [C= 2, SC= 20, EC= 2]: HRMS ES^+ for $C_{23}H_{19}ClN_3O_2$ *m/z*: $[M+H]^+$ Calc. 404.2354, found: 404.2335.

3. ^{1}H NMR, ^{13}C -NMR, DEPT 135, COSY NMR, HSQC NMR, HMBC NMR, NOESY NMR and FT-IR spectra of the products 5

Methyl-1-(phenylamino)-1*H*-imidazo[2,1-*a*]isoindole-2,5(3*H*,9*bH*)-dione (5a)

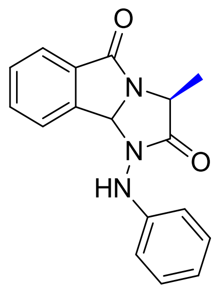

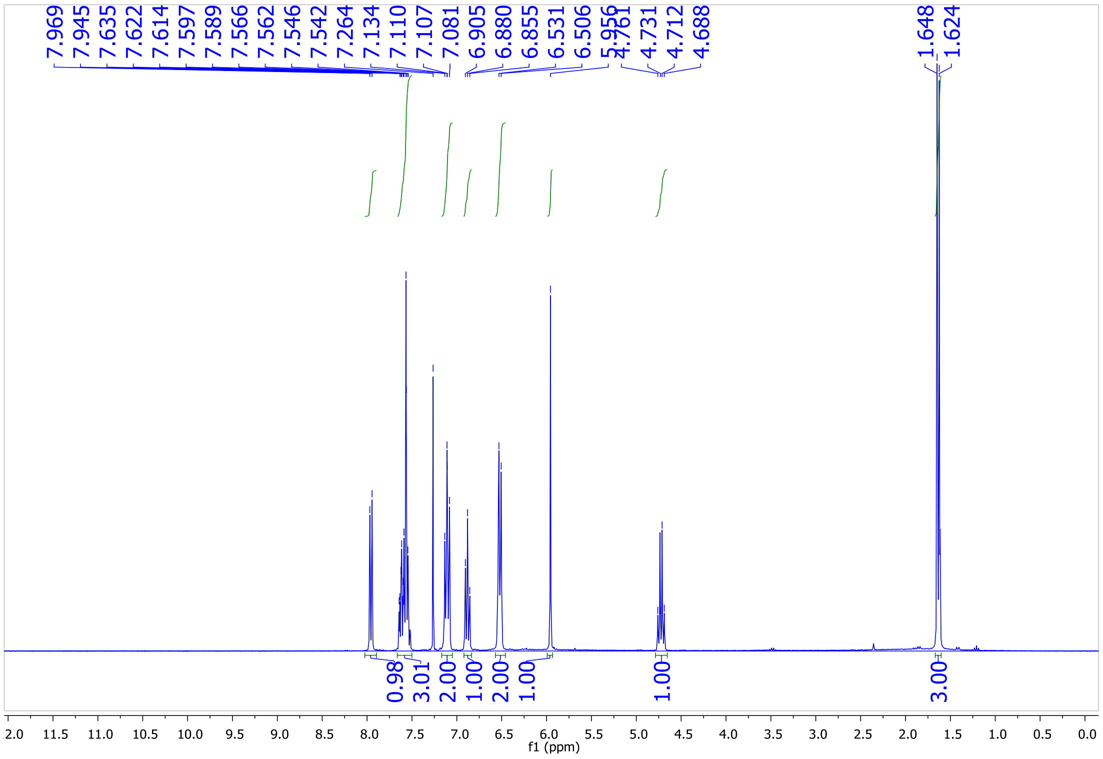

^{1}H NMR spectrum of the compound **5a** in $CDCl_3$ at 300 MHz

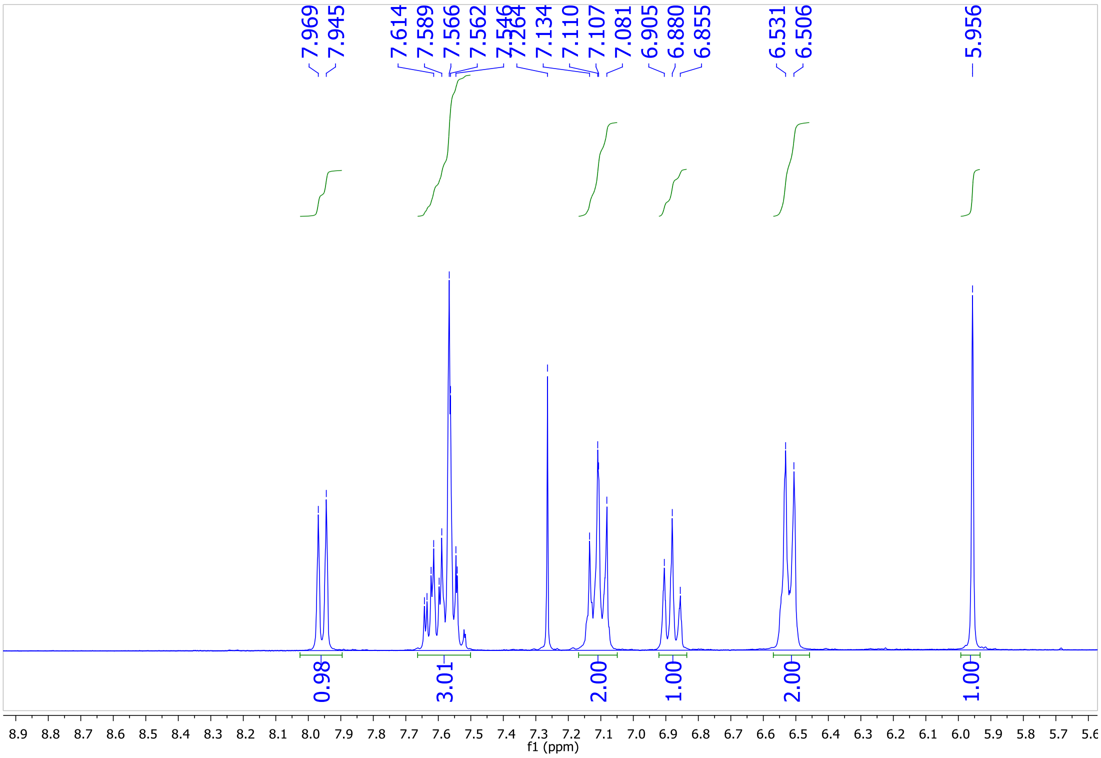

^{1}H NMRspectrum of the compound **5a** in $CDCl_3$ at 300 MHz

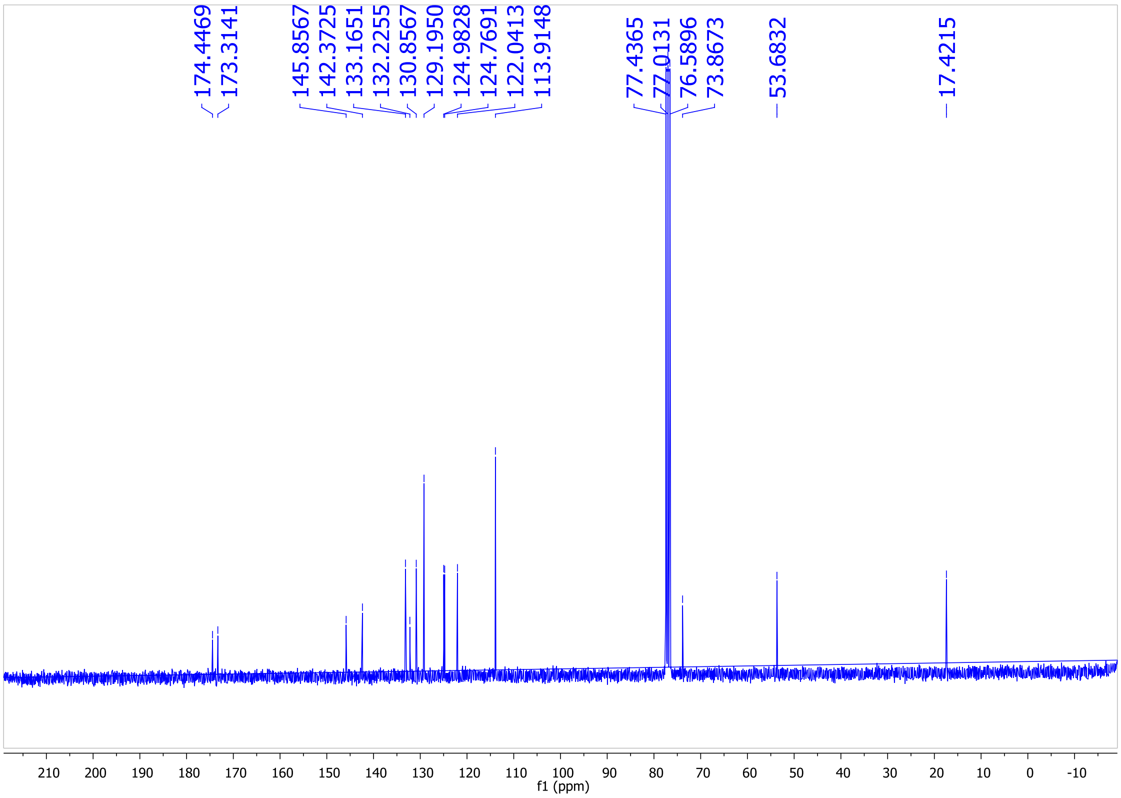

^{13}C NMRspectrum of the compound **5a** in $CDCl_3$ at 75 MHz

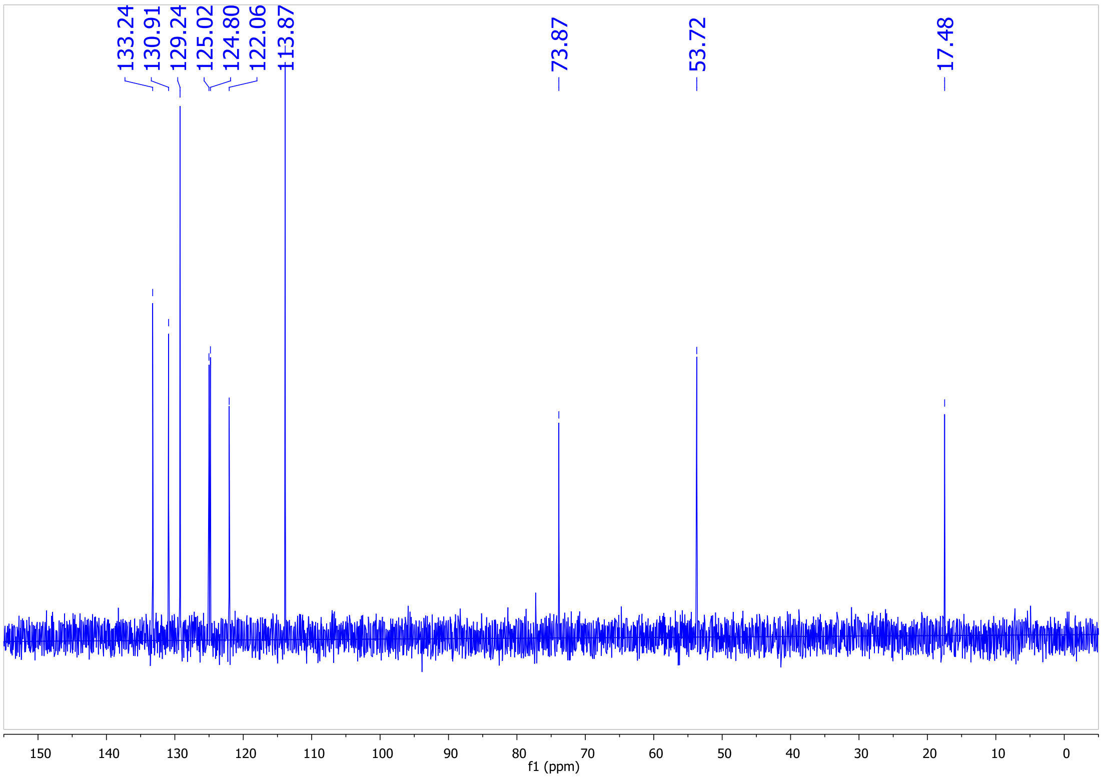

DEPT 135 NMR spectrum of the compound **5a** in $CDCl_3$ at 75 MHz

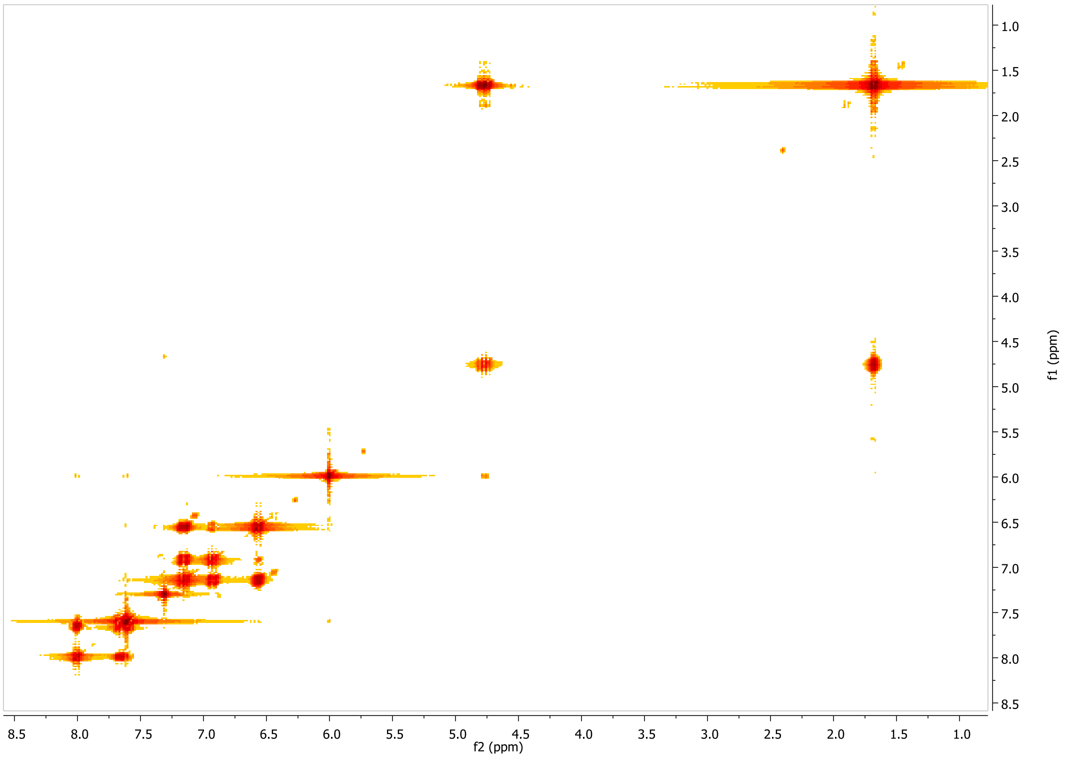

COSY NMR spectrum of the compound **5a** in $CDCl_3$

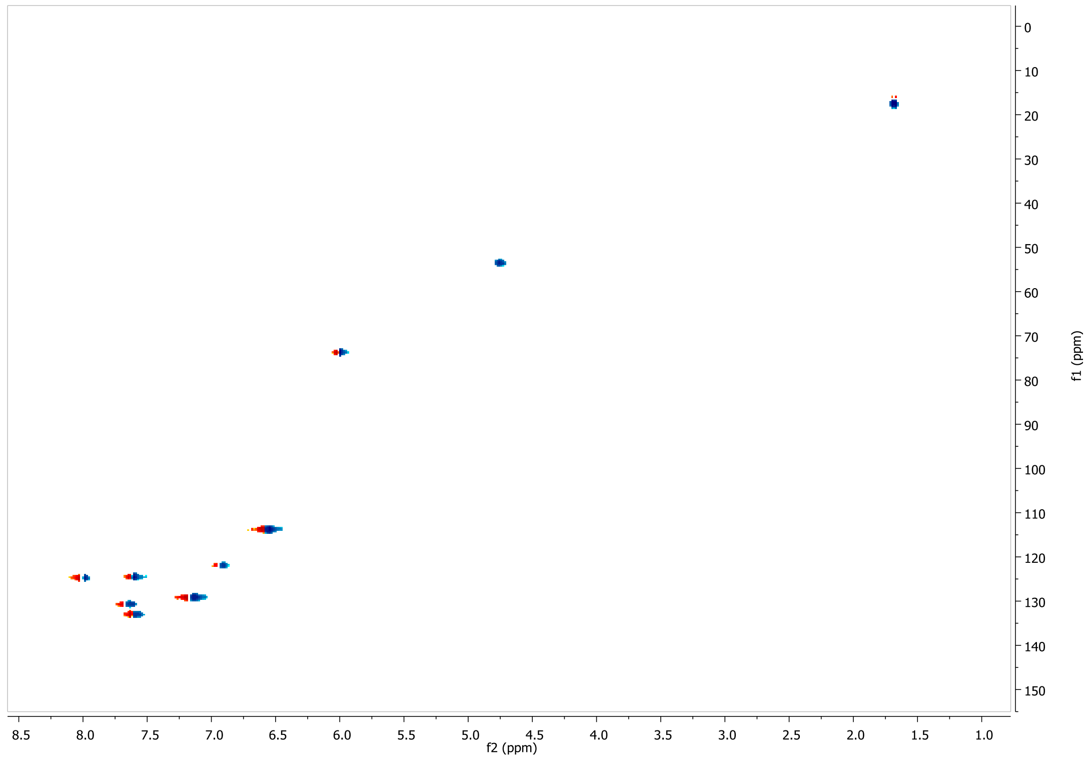

HSQC NMR spectrum of the compound **5a** in $CDCl_3$

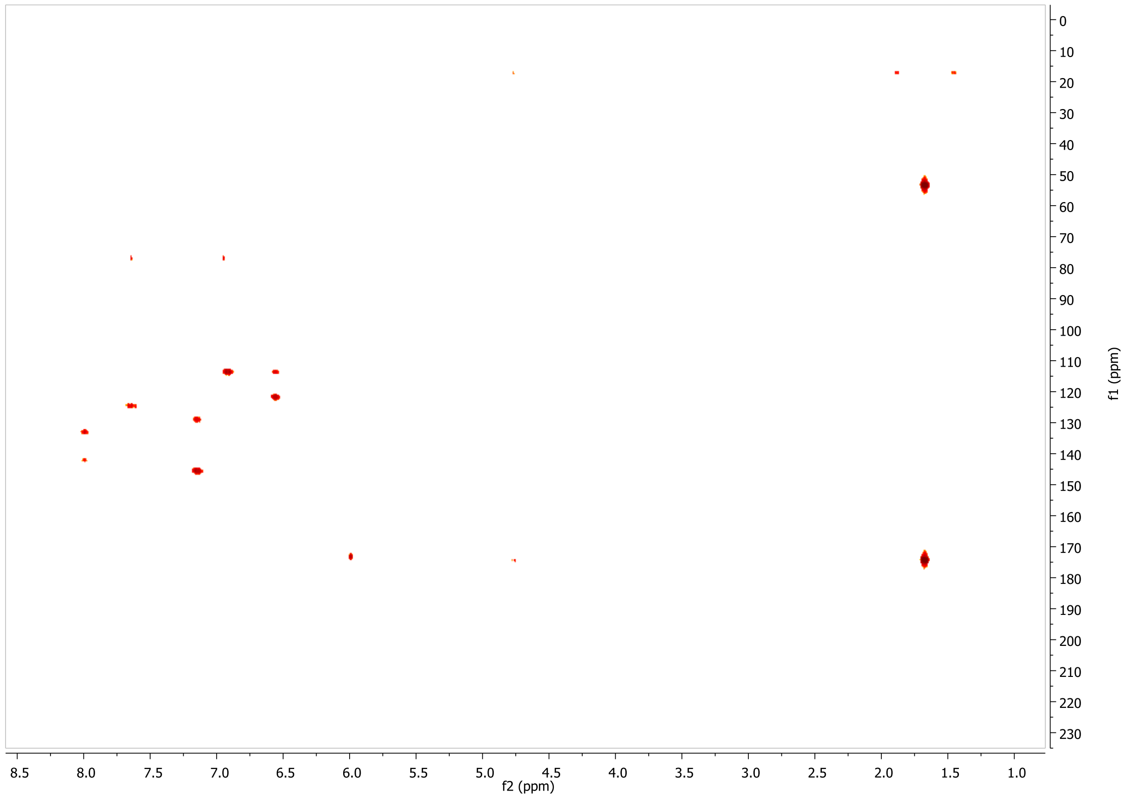

HMBC NMR spectrum of the compound **5a** in $CDCl_3$

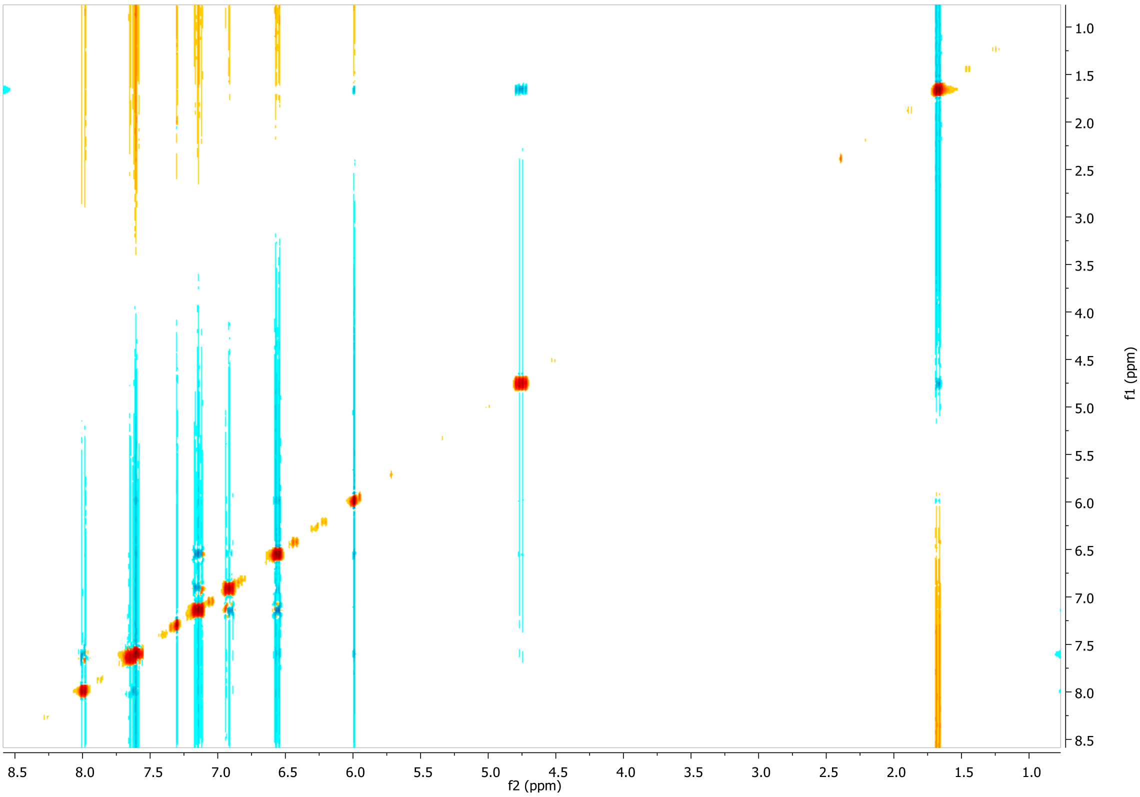

NOESY NMR spectrum of the compound **5a** in $CDCl_3$

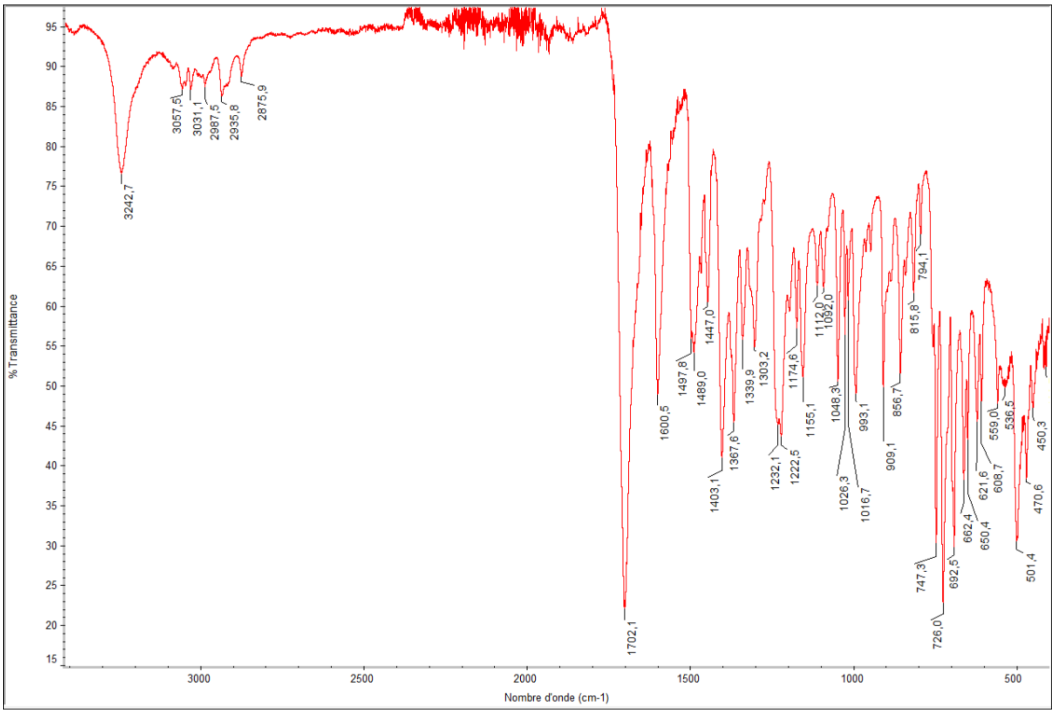

FT-IR spectrum of the compound **5a**

3-Isopropyl-1-(phenylamino)-1*H*-imidazo[2,1-*a*]isoindole-2,5(3*H*,9b*H*)-dione (5b)

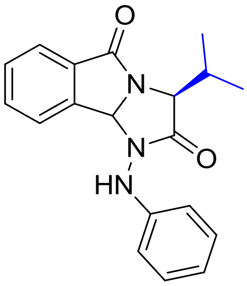

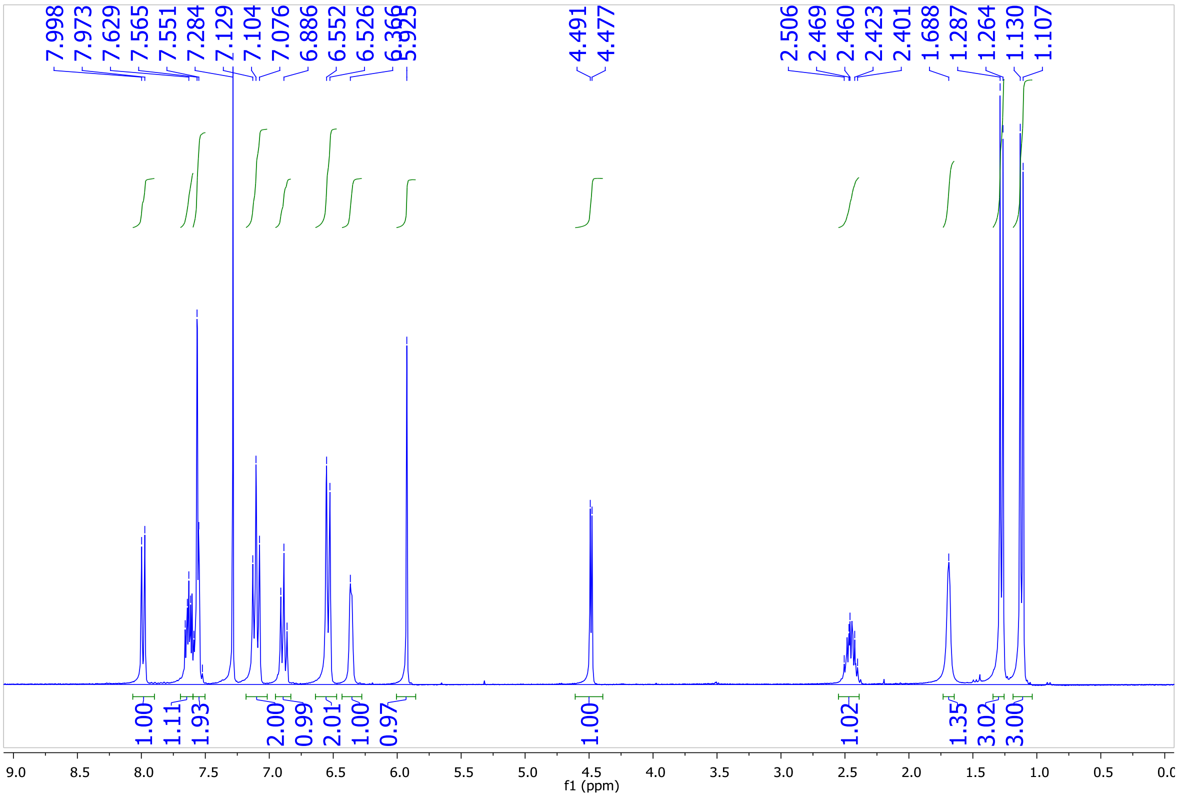

^{1}H NMRspectrum of the compound **5b** in $CDCl_3$ at 300 MHz

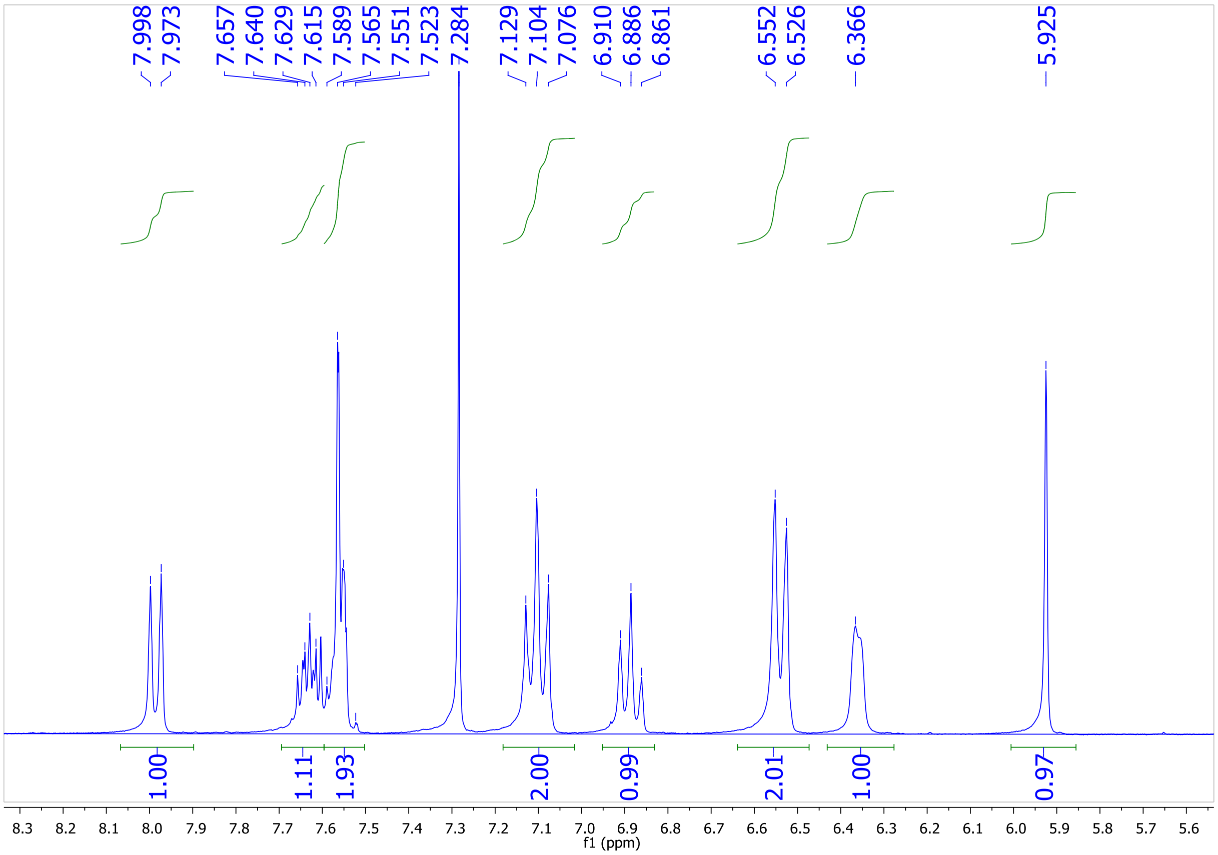

^{1}H NMRspectrum of the compound **5b** in $CDCl_3$ at 300 MHz

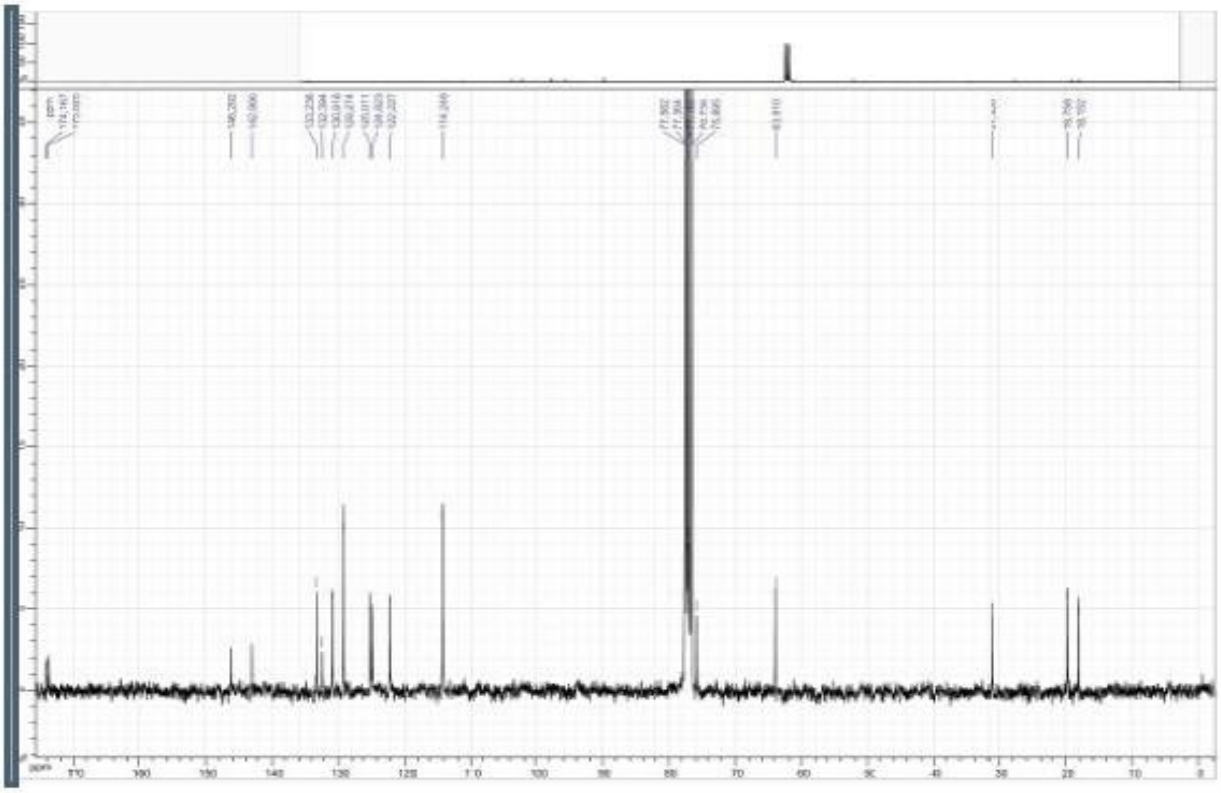

^{13}C NMRspectrum of the compound **5b** in $CDCl_3$ at 75 MHz

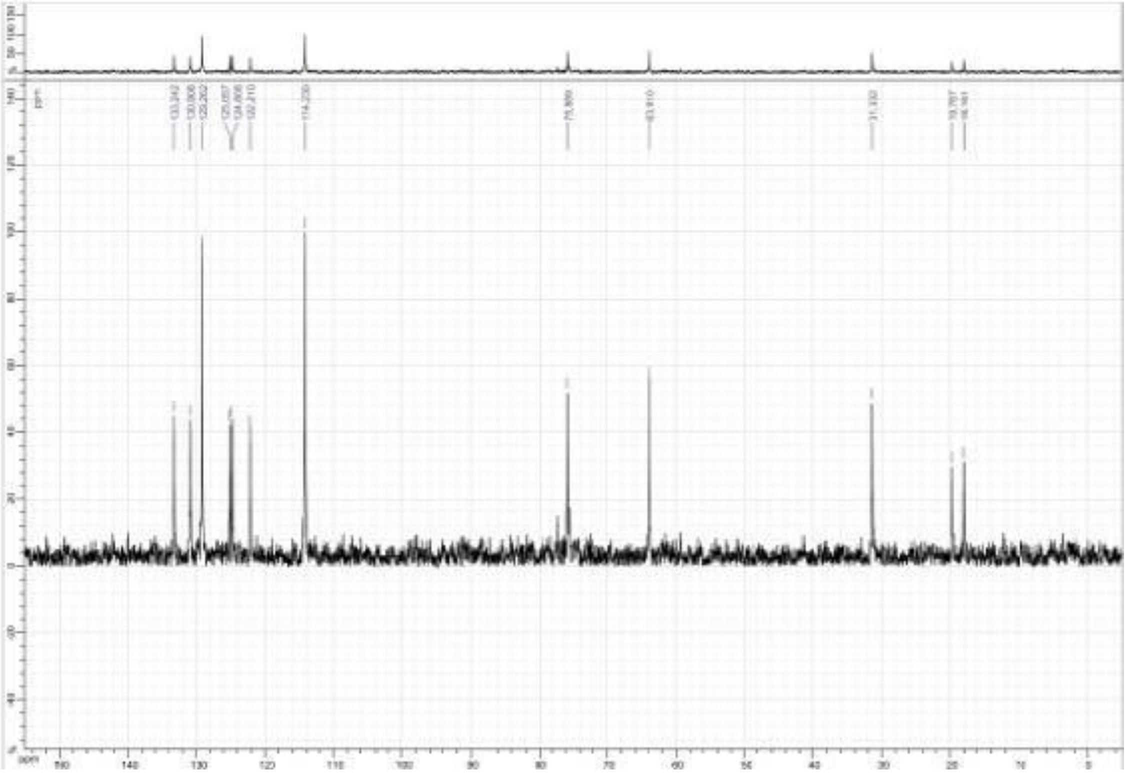

DEPT 135 NMR spectrum of the compound **5b** in $CDCl_3$ at 75 MHz

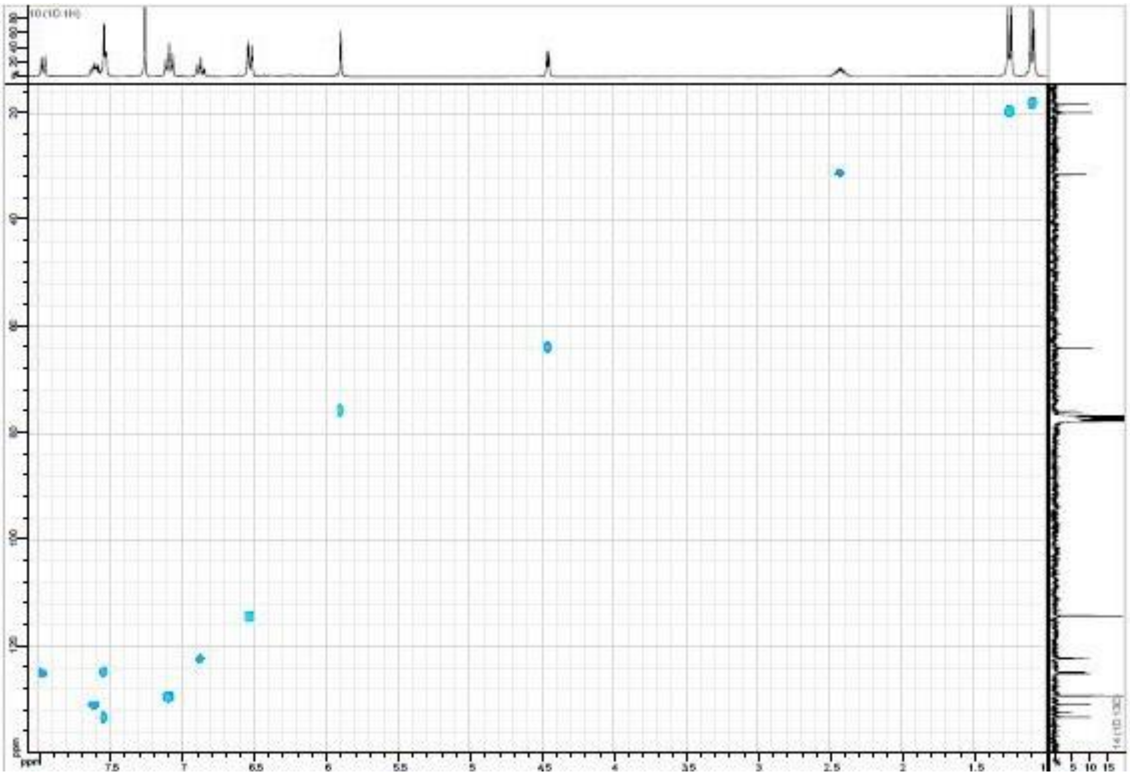

HSQC NMR spectrum of the compound **5b** in $CDCl_3$

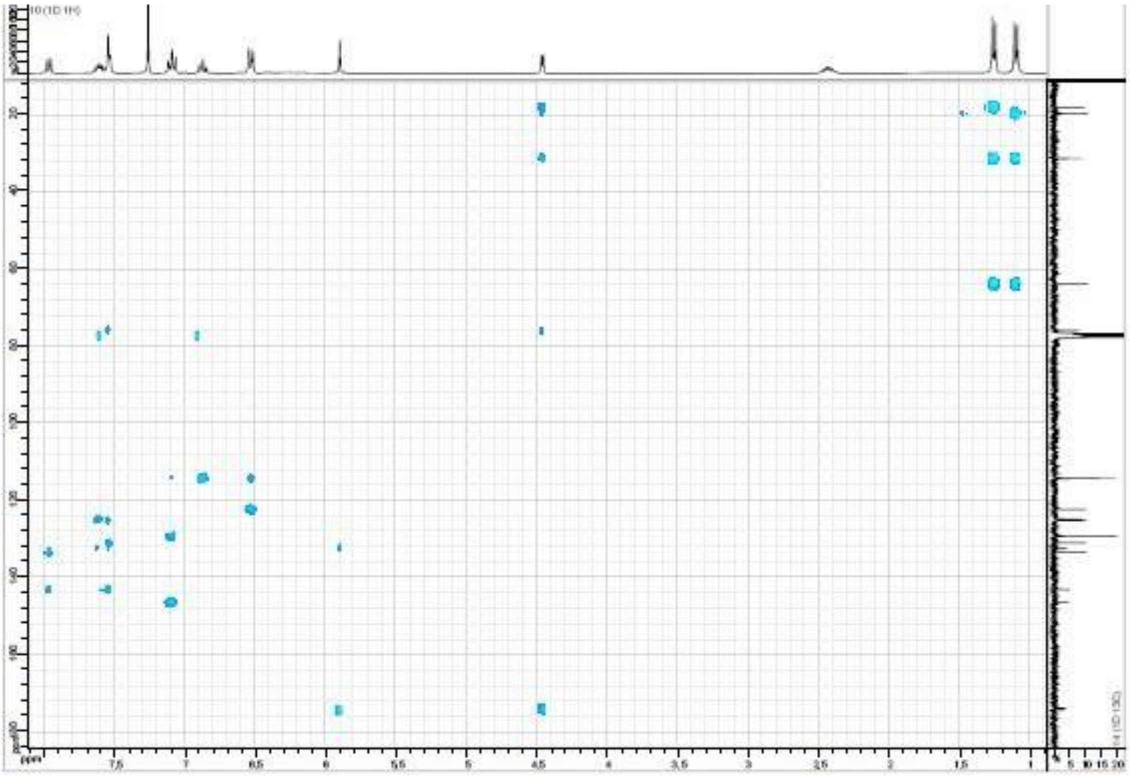

HMBC NMR spectrum of the compound **5b** in $CDCl_3$

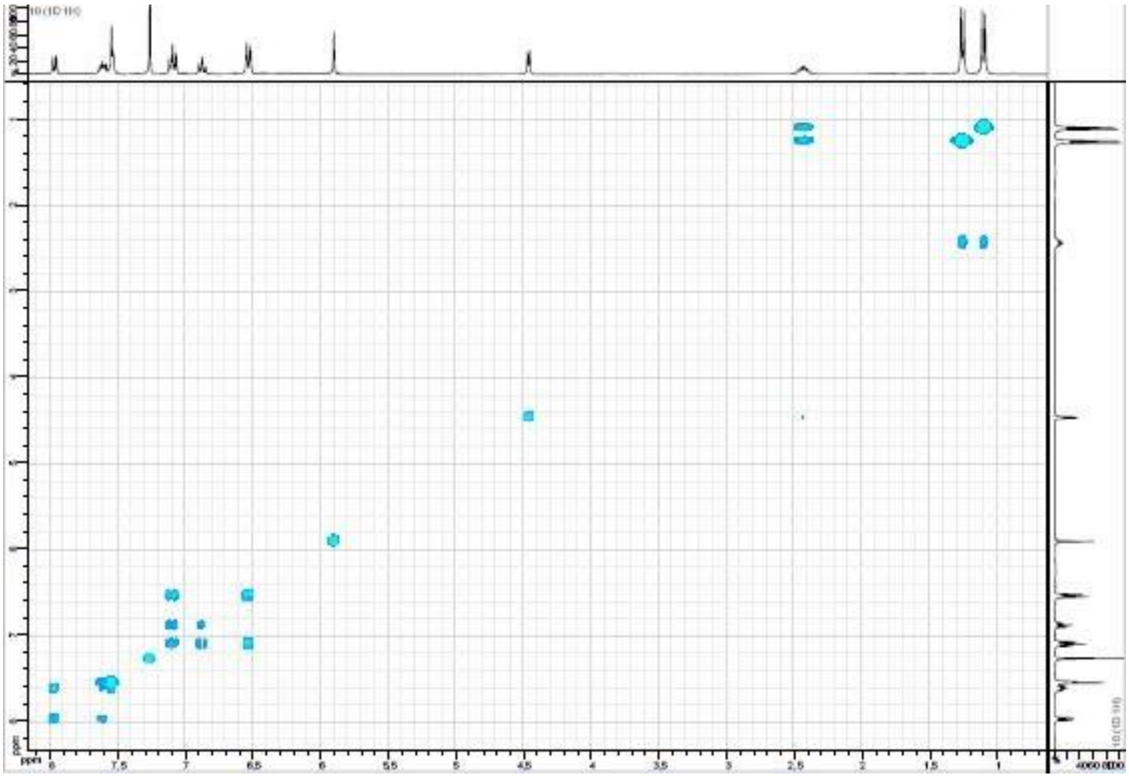

COSY NMR spectrum of the compound **5b** in $CDCl_3$

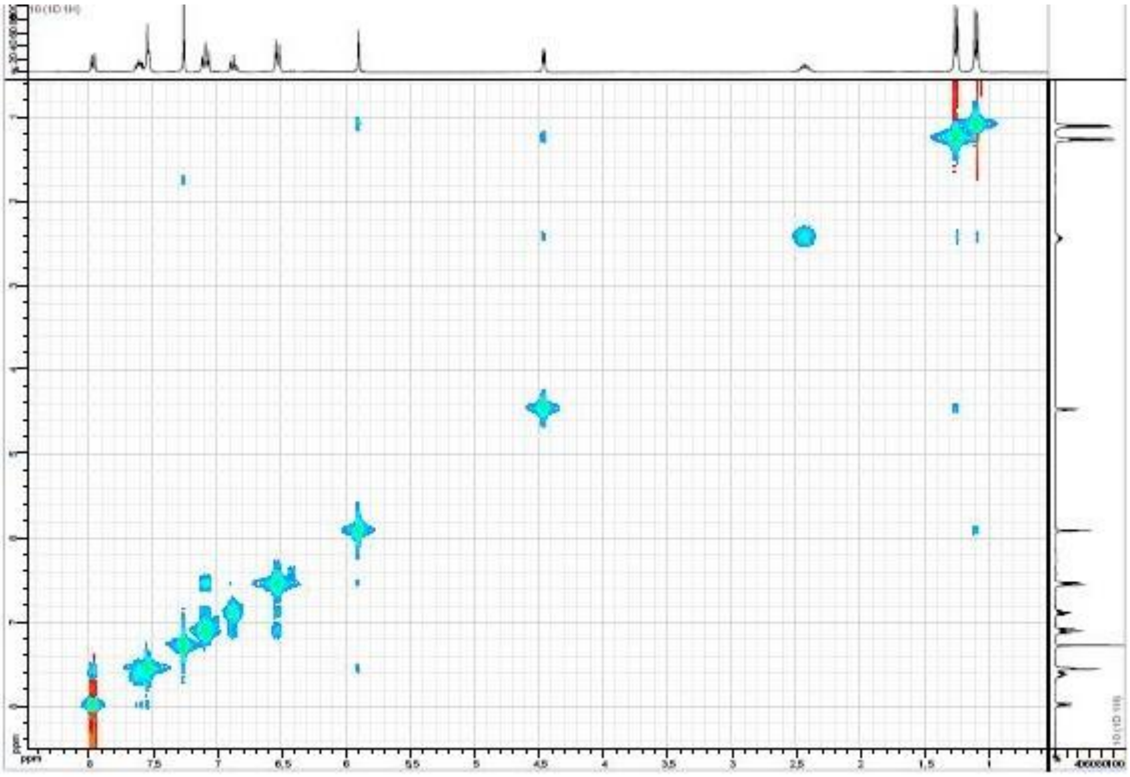

NOESY NMR spectrum of the compound **5b** in $CDCl_3$

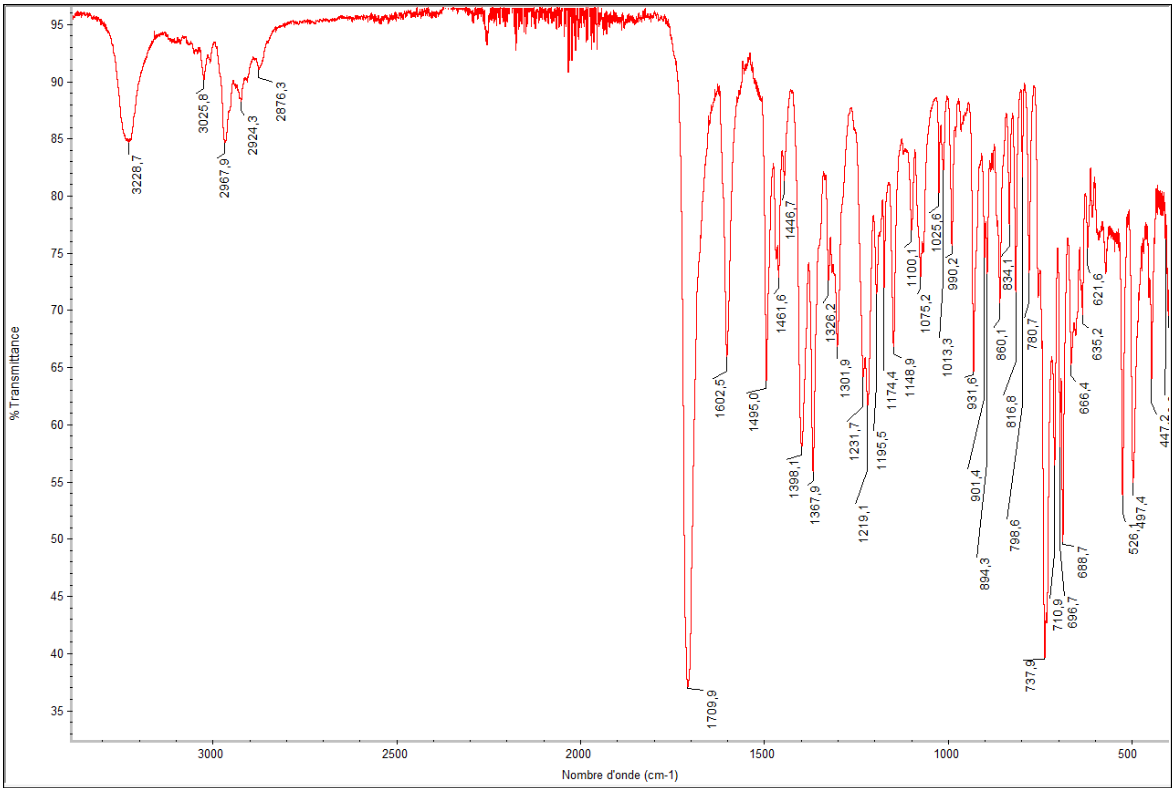

FT-IR spectrum of the compound **5b**

3-Isobutyl-1-(phenylamino)-1*H*-imidazo[2,1-*a*]isoindole-2,5(3*H*,9b*H*)-dione (5c)

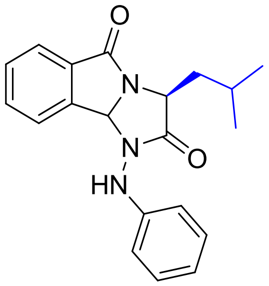

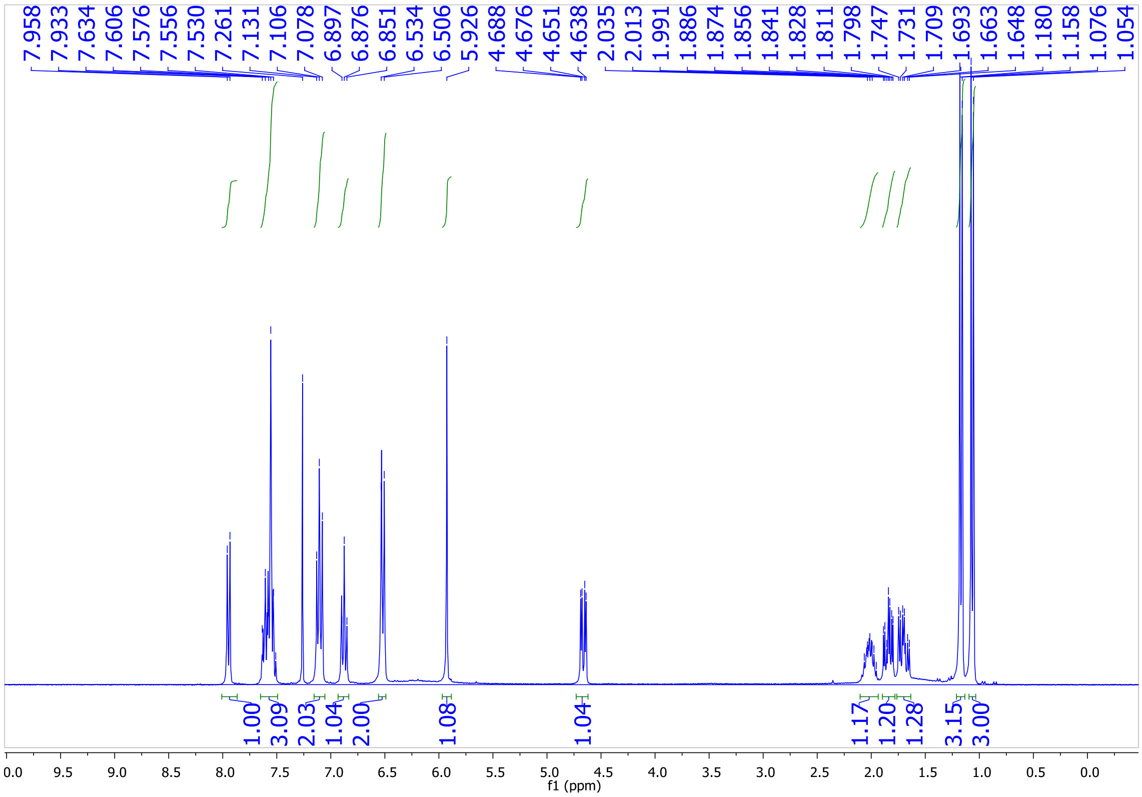

^{1}H NMRspectrum of the compound **5c** in $CDCl_3$ at 300 MHz

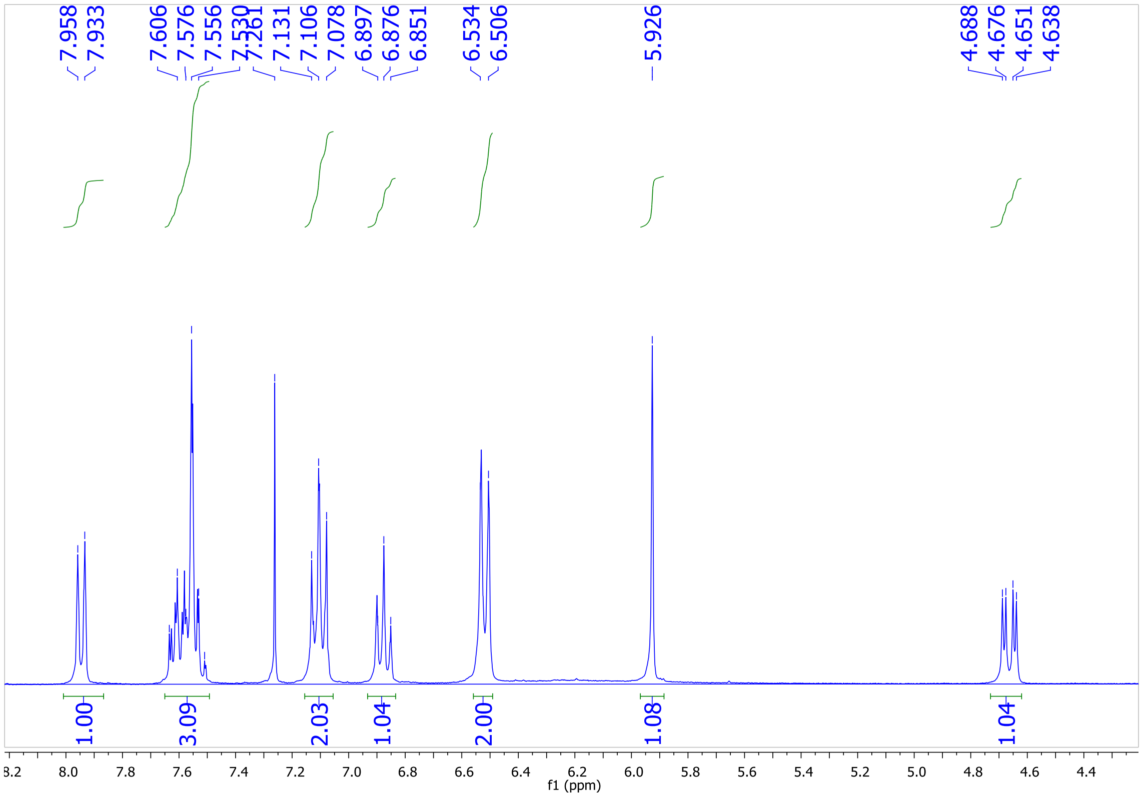

^{1}H NMRspectrum of the compound **5c** in $CDCl_3$ at 300 MHz

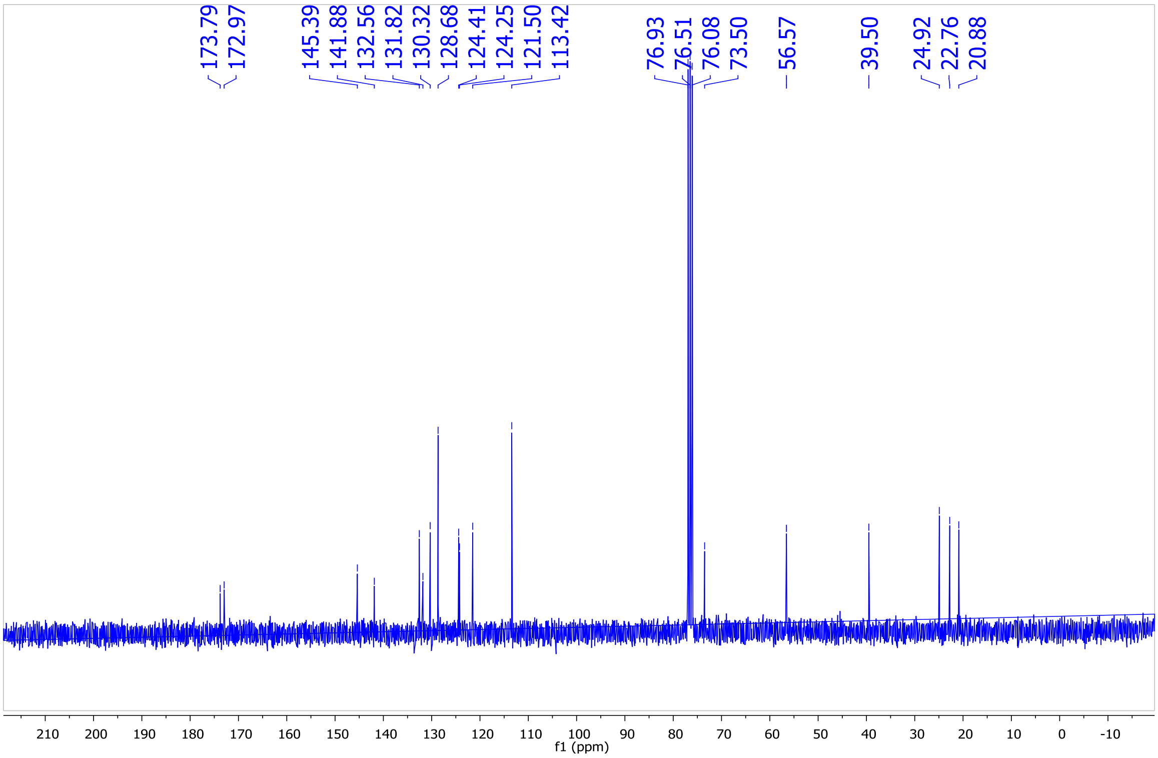

^{13}C NMRspectrum of the compound **5c** in $CDCl_3$ at 75 MHz

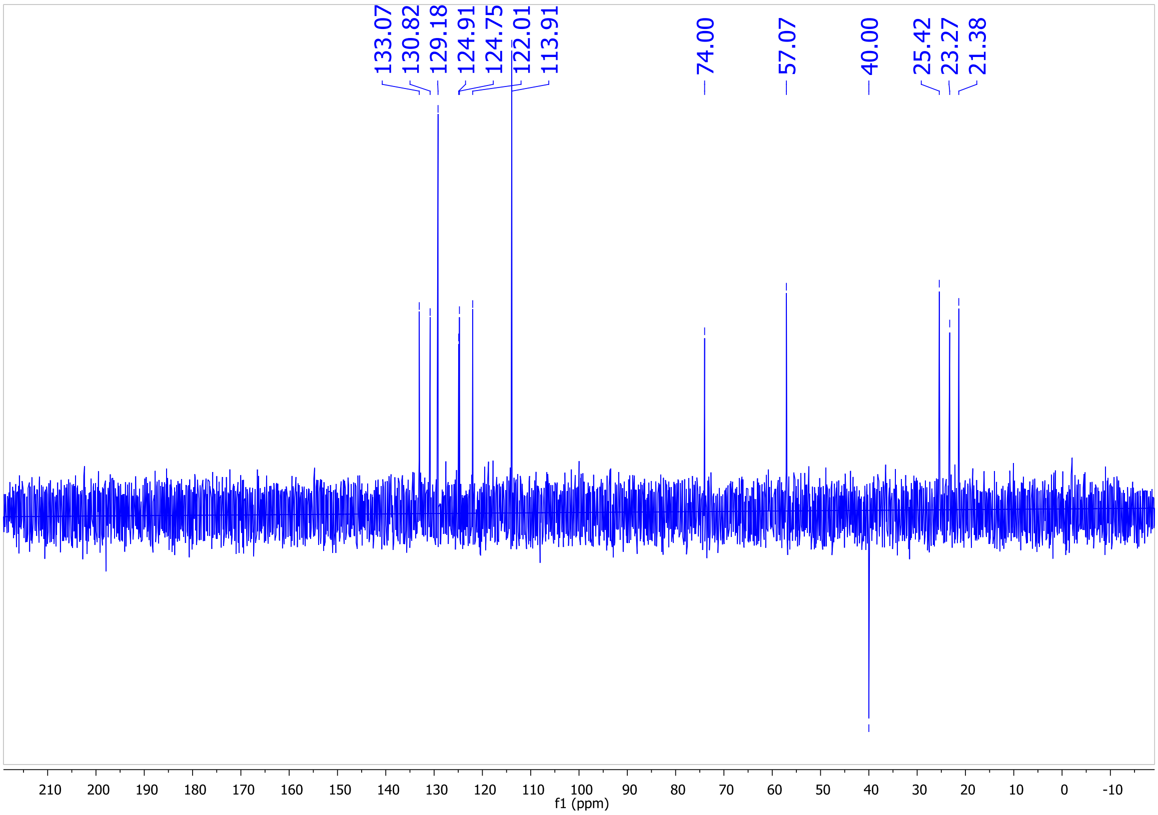

DEPT 135 NMR spectrum of the compound **5c** in $CDCl_3$ at 75 MHz

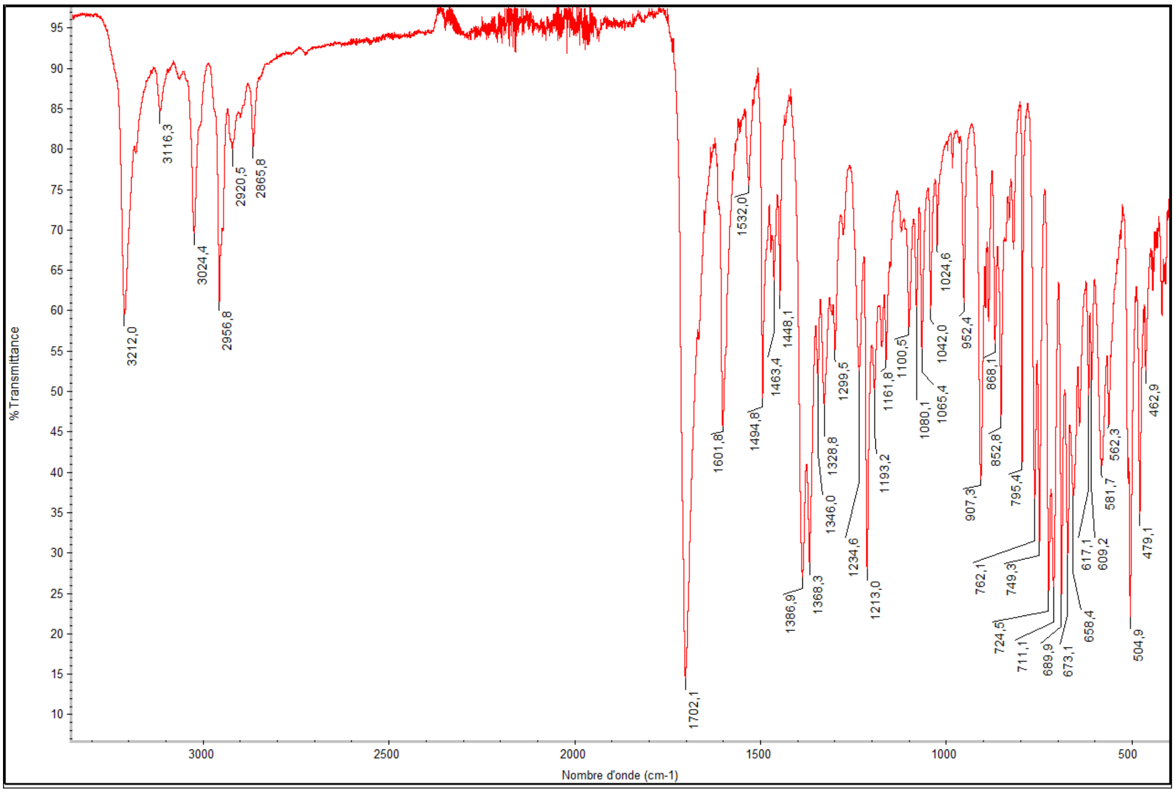

FT-IR spectrum of the compound **5c**

3-Phenyl-1-(phenylamino)-1*H*-imidazo[2,1-*a*]isoindole-2,5(3*H*,9b*H*)-dione (5d)

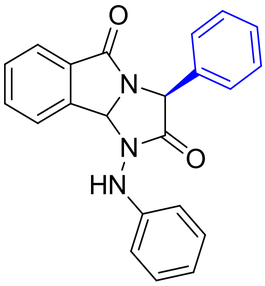

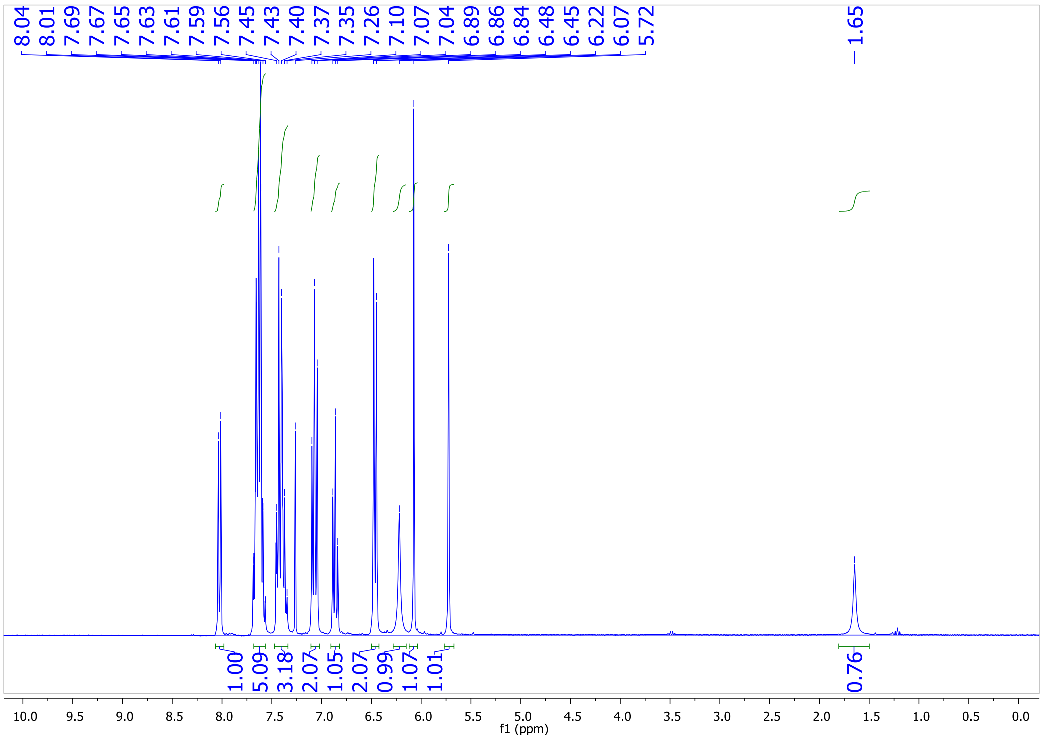

^{1}H NMRspectrum of the compound **5d** in $CDCl_3$ at 300 MHz

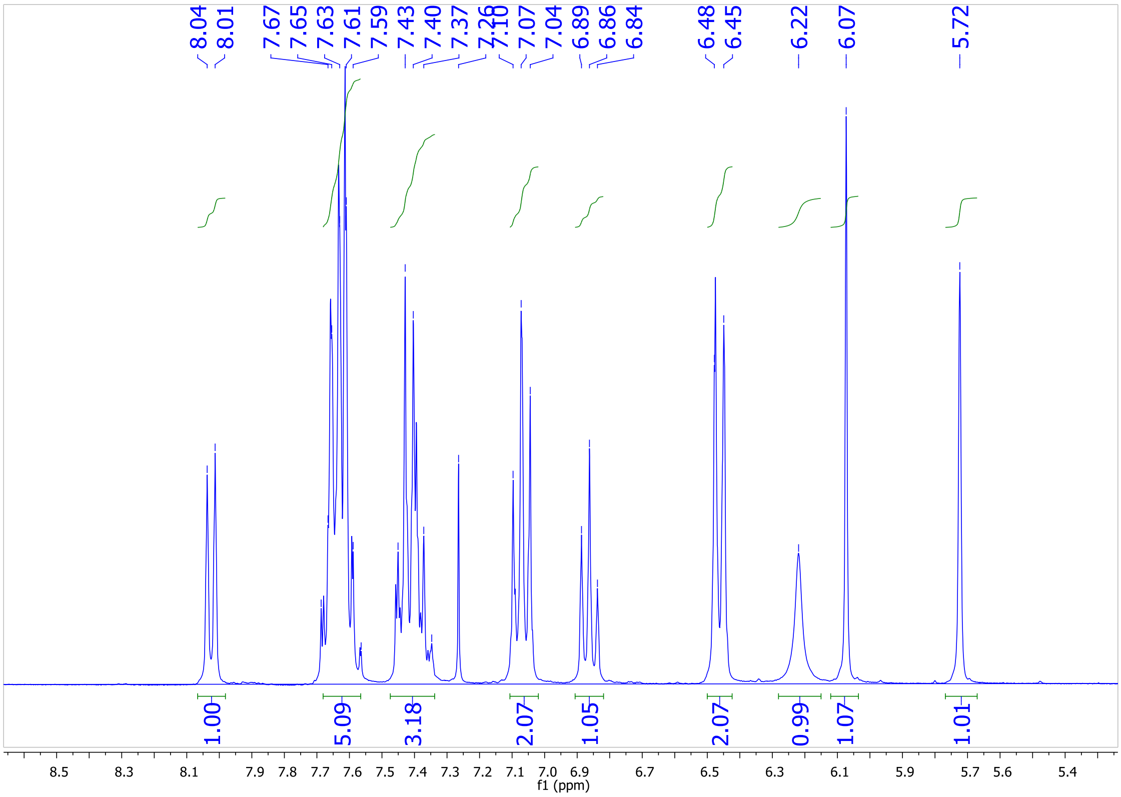

^{1}H NMRspectrum of the compound **5d** in $CDCl_3$ at 300 MHz

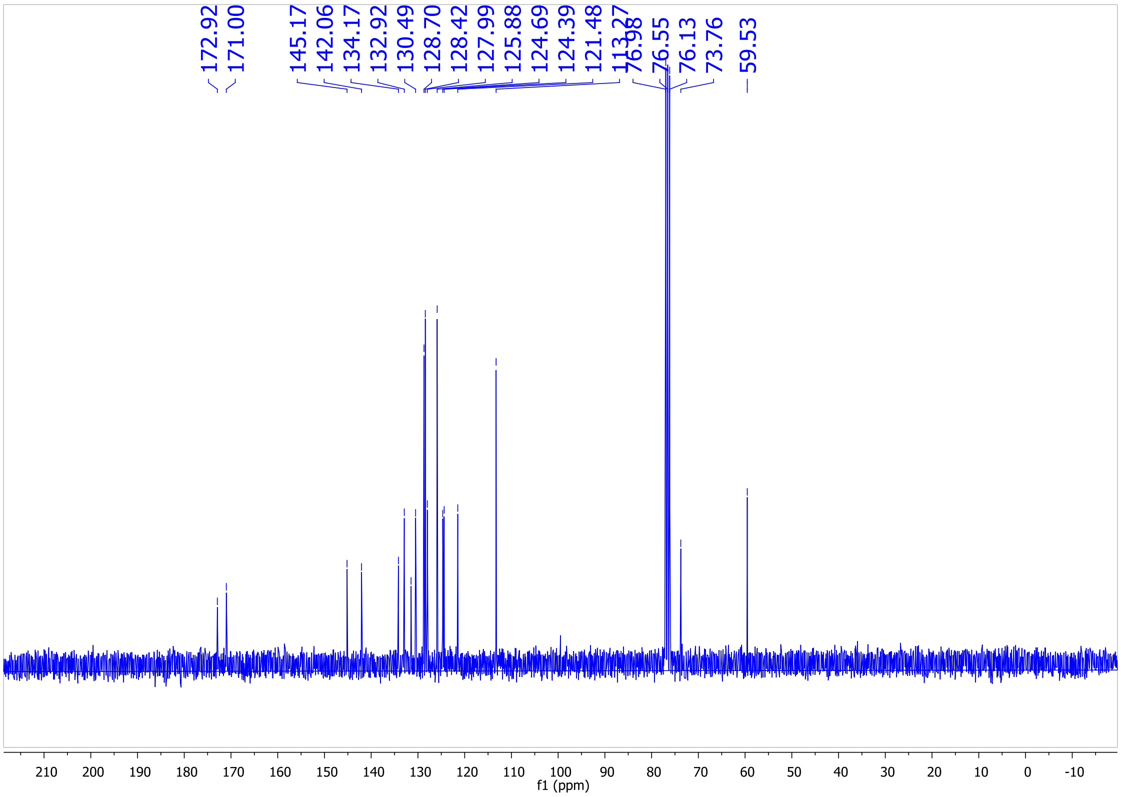

^{13}C NMRspectrum of the compound **5d** in $CDCl_3$ at 75 MHz

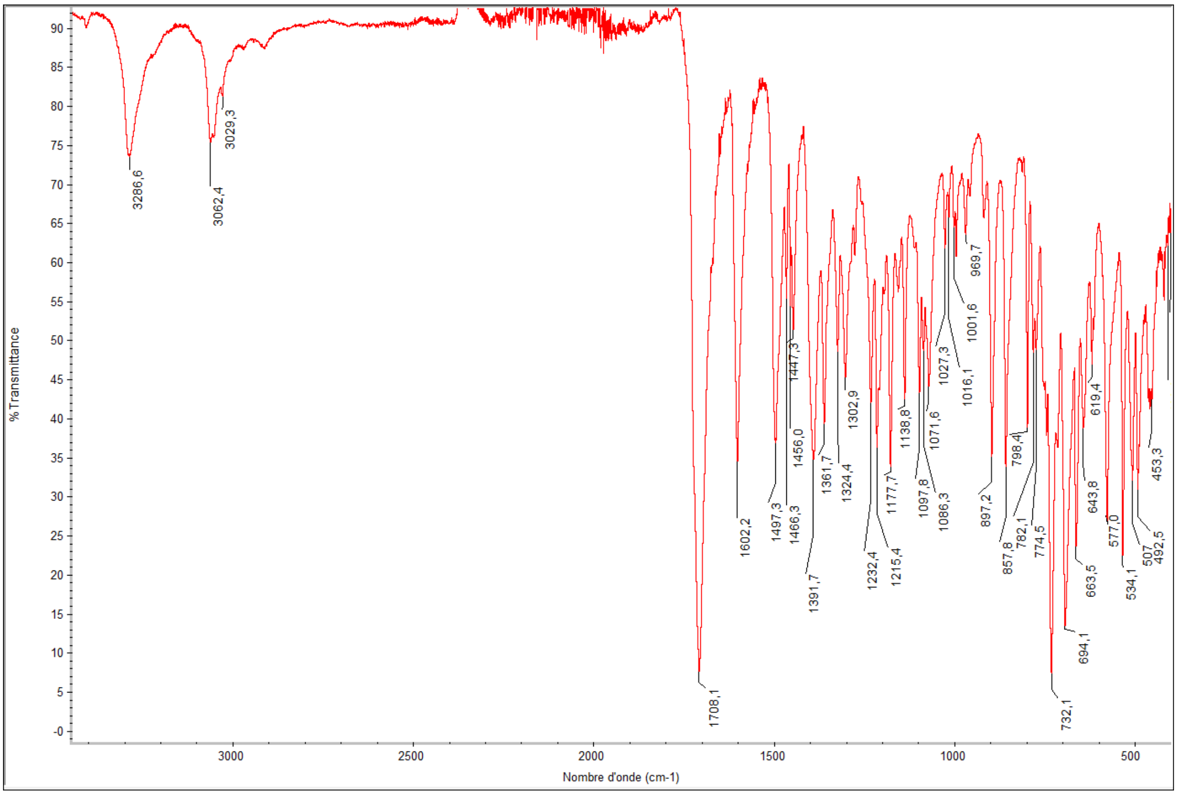

FT-IR spectrum of the compound **5d**

3-Benzyl-1-(phenylamino)-1*H*-imidazo[2,1-*a*]isoindole-2,5(3*H*,9b*H*)-dione (5e)

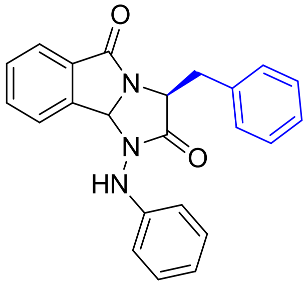

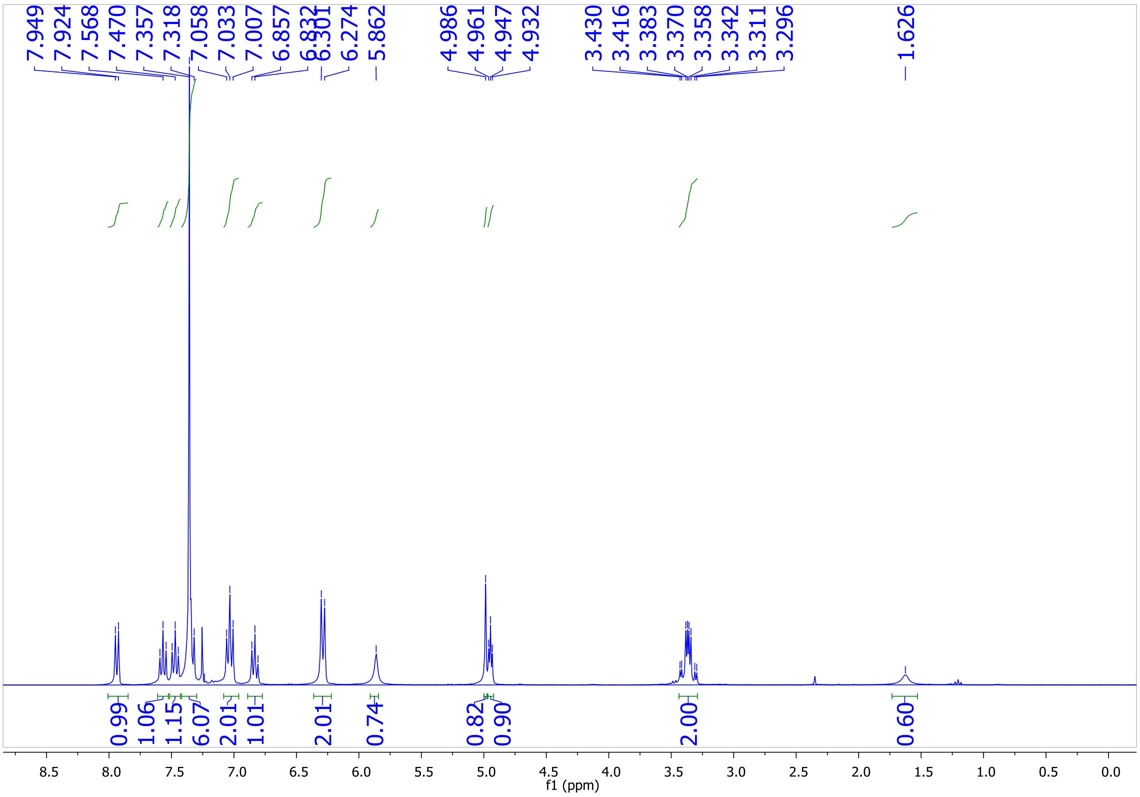

^{1}H NMRspectrum of the compound **5e** in $CDCl_3$ at 300 MHz

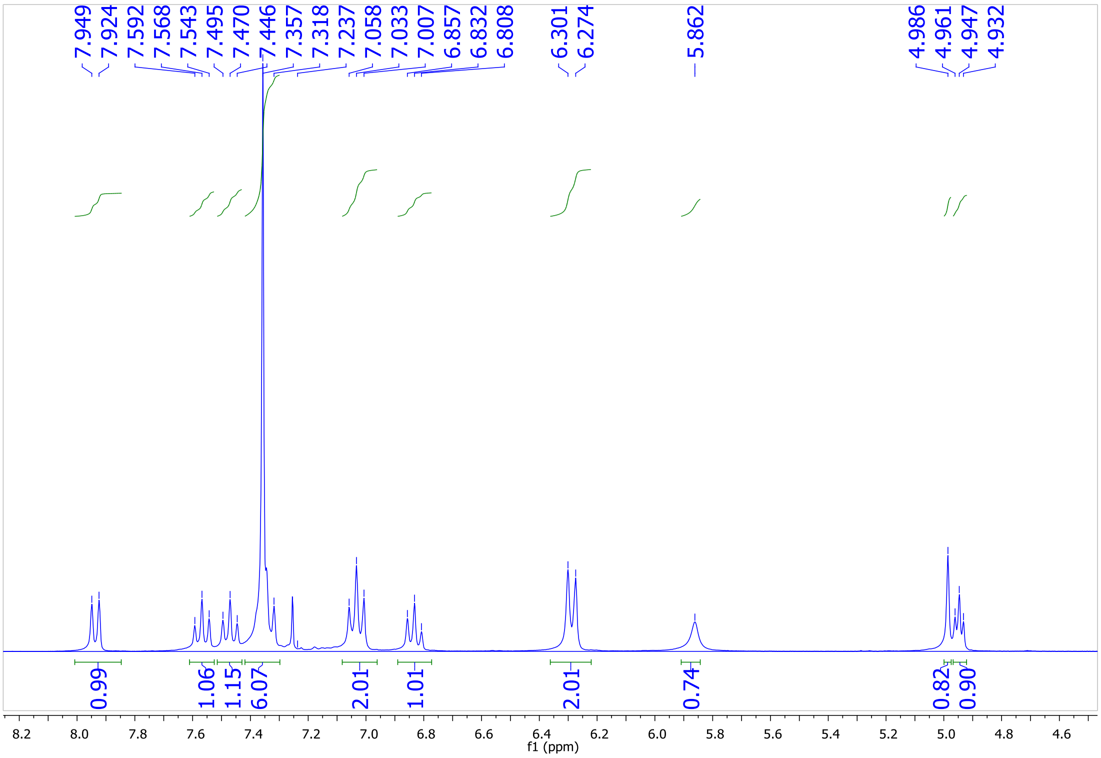

^{1}H NMRspectrum of the compound **5e** in $CDCl_3$ at 300 MHz

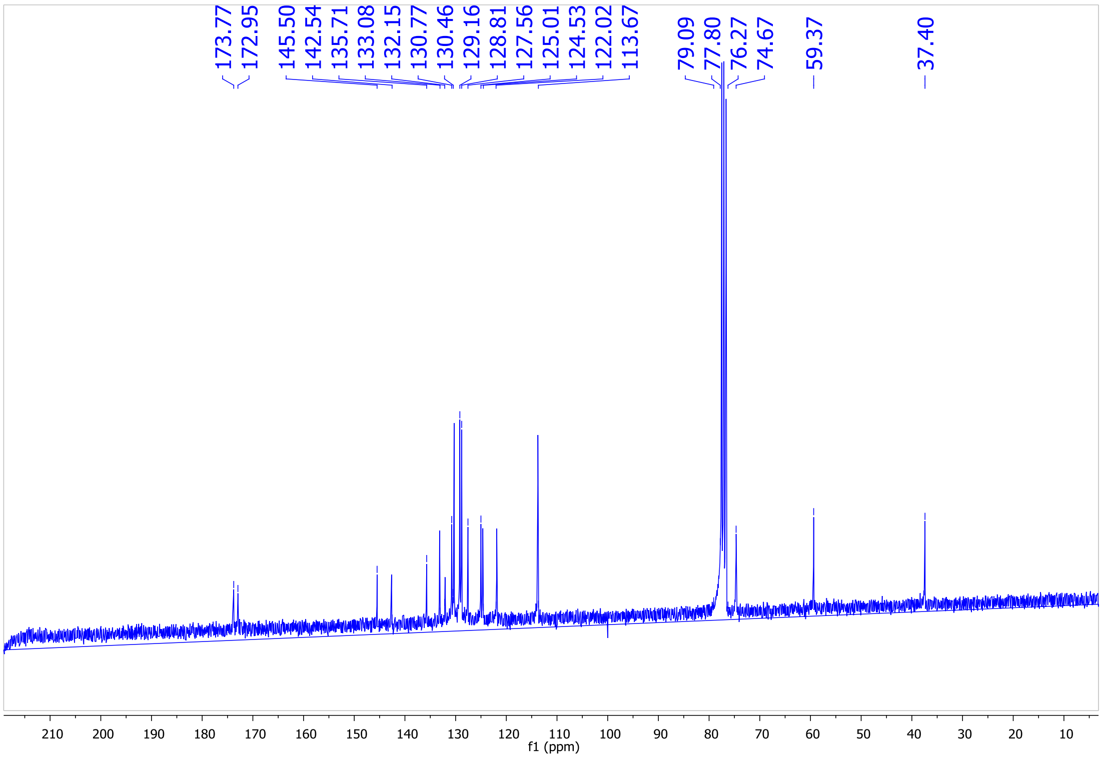

^{13}C NMRspectrum of the compound **5e** in $CDCl_3$ at 75 MHz

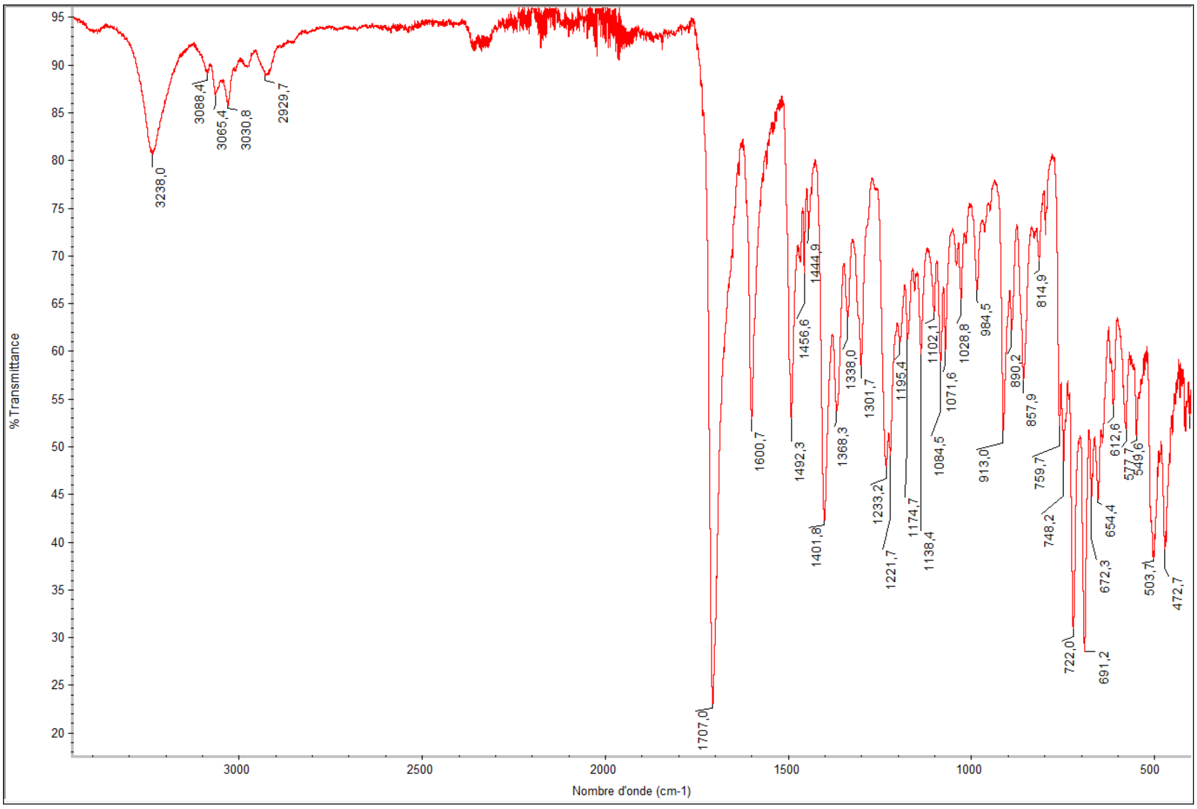

FT-IR spectrum of the compound **5e**

3-(2-(Methylthio)ethyl)-1-(phenylamino)-1*H*-imidazo[2,1-*a*]isoindole-2,5(3*H*,9b*H*)-dione (5f)

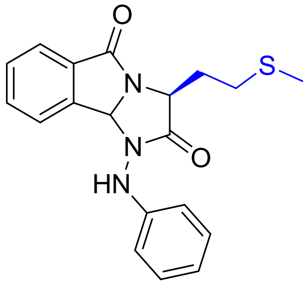

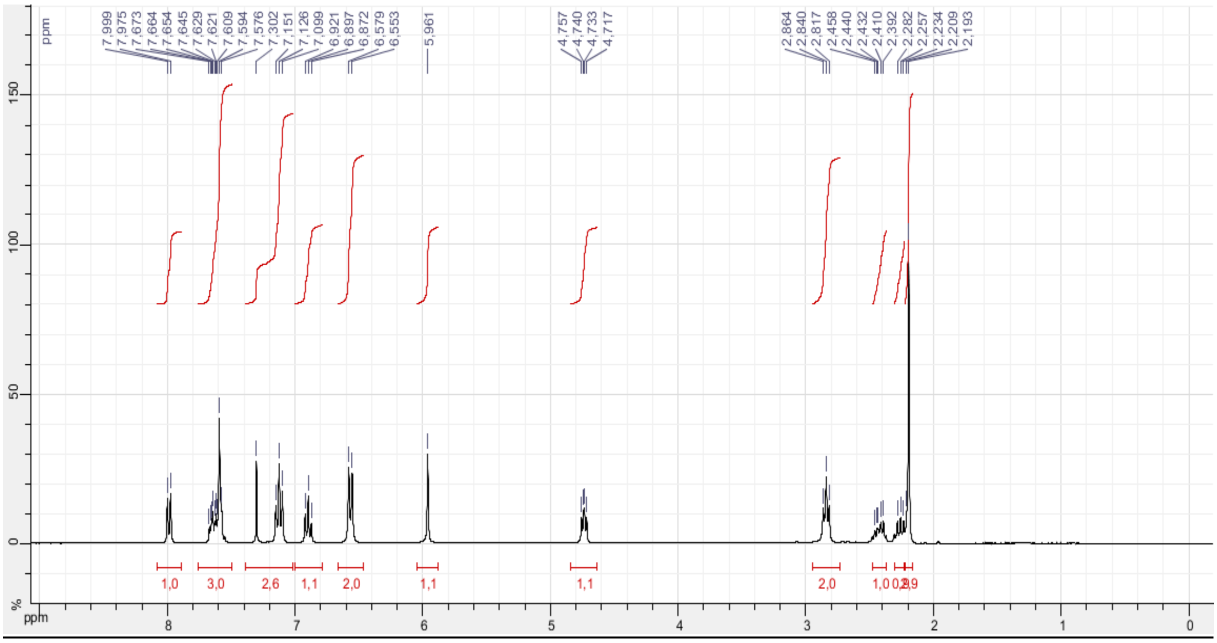

^{1}H NMRspectrum of the compound **5f** in $CDCl_3$ at 300 MHz

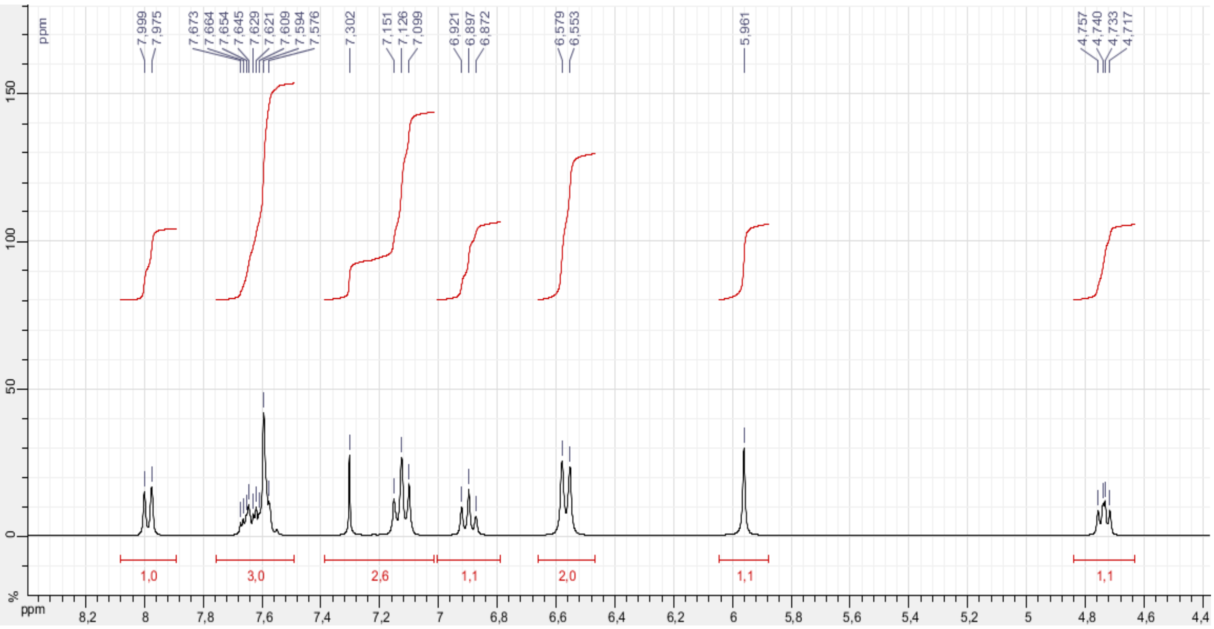

^{1}H NMRspectrum of the compound **5f** in $CDCl_3$ at 300 MHz

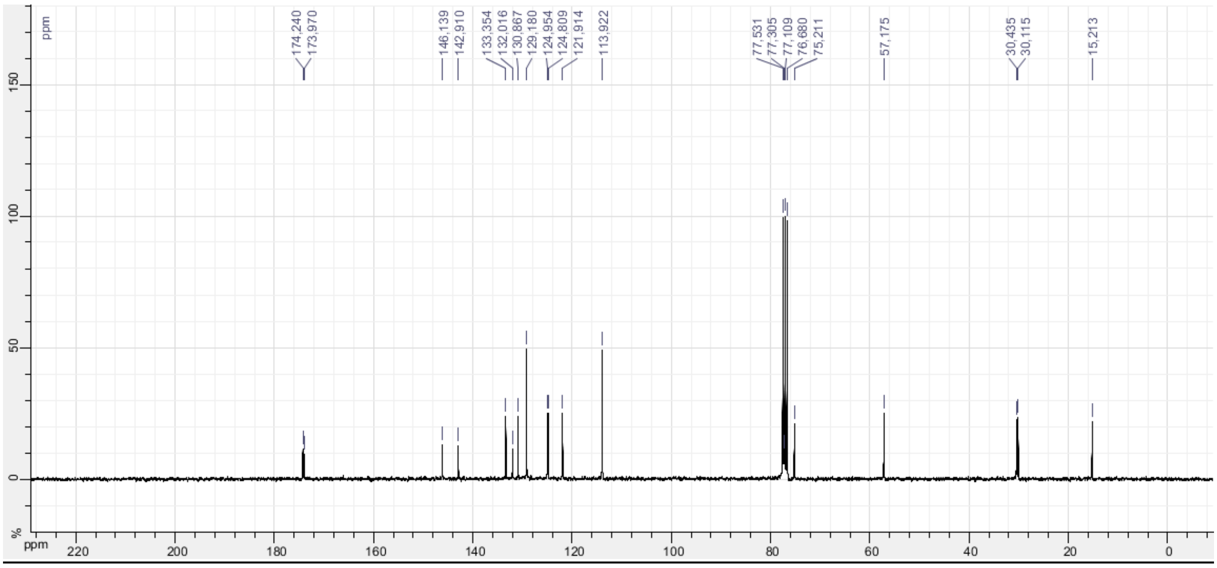

^{13}C NMRspectrum of the compound **5f** in $CDCl_3$ at 75 MHz

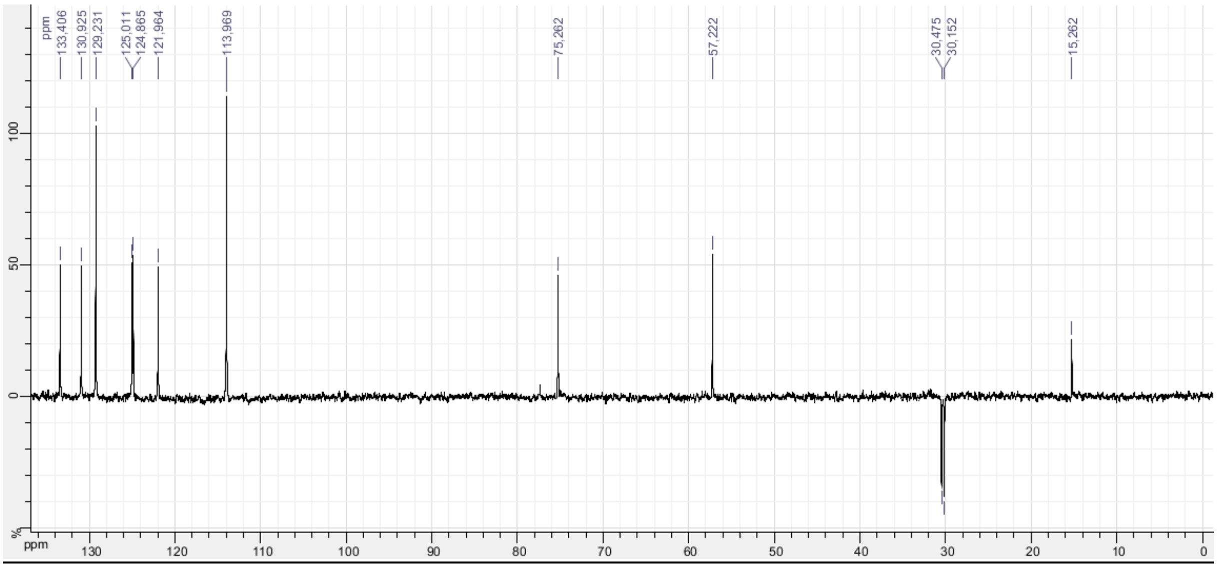

DEPT 135 NMR spectrum of the compound **5f** in $CDCl_3$ at 75 MHz

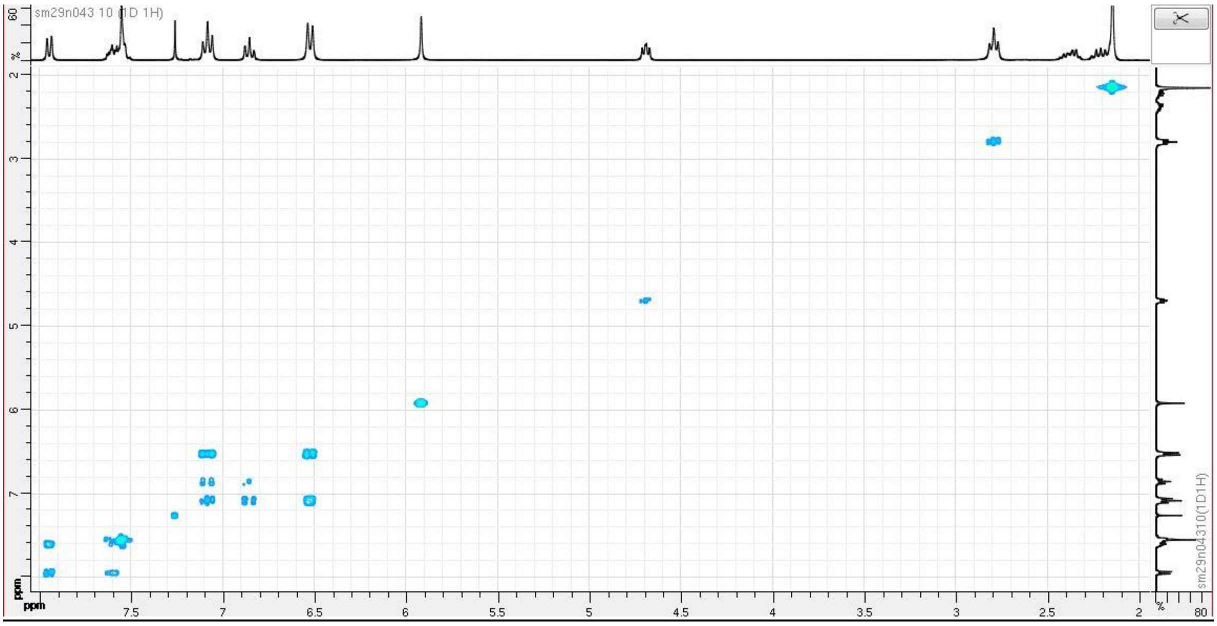

COSY NMR spectrum of the compound **5f** in $CDCl_3$ at 300 MHz

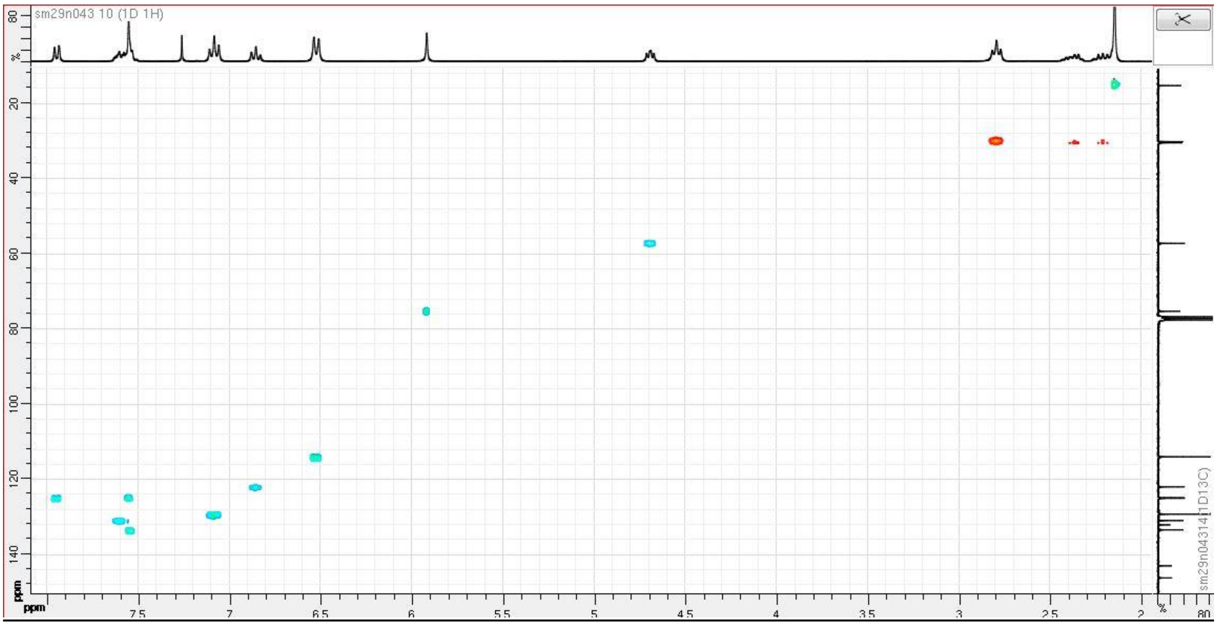

HSQC NMR spectrum of the compound **5f** in $CDCl_3$ at 300 MHz

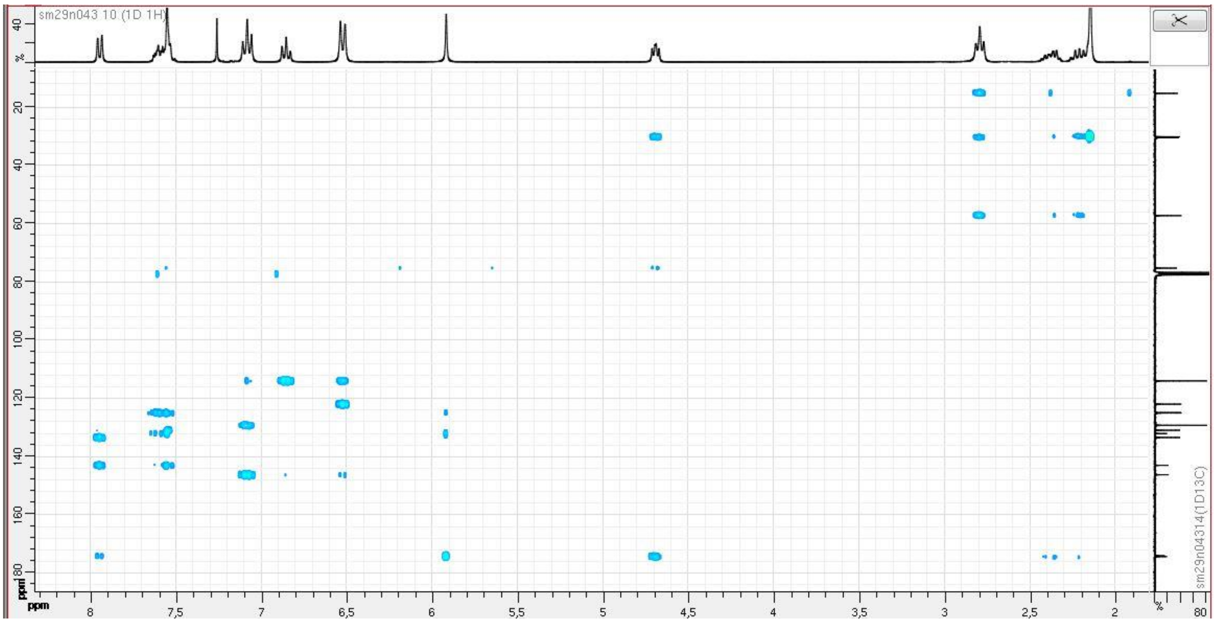

HMBC NMR spectrum of the compound **5f** in $CDCl_3$ at 300 MHz

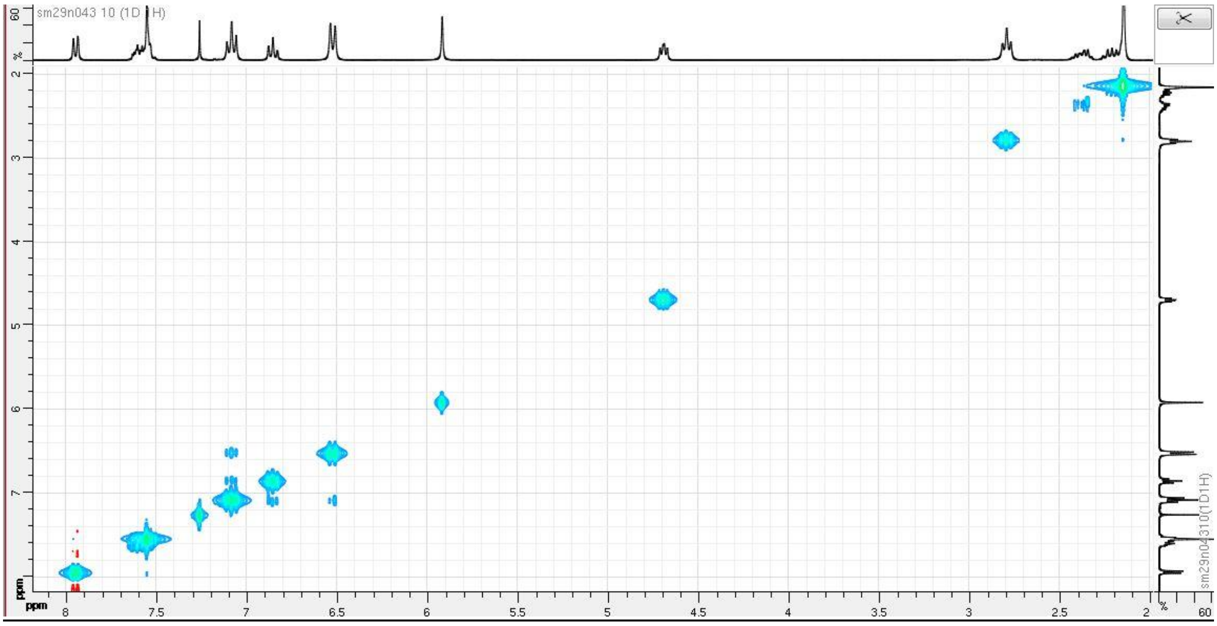

NOESY NMR spectrum of the compound **5f** in $CDCl_3$ at 300 MHz

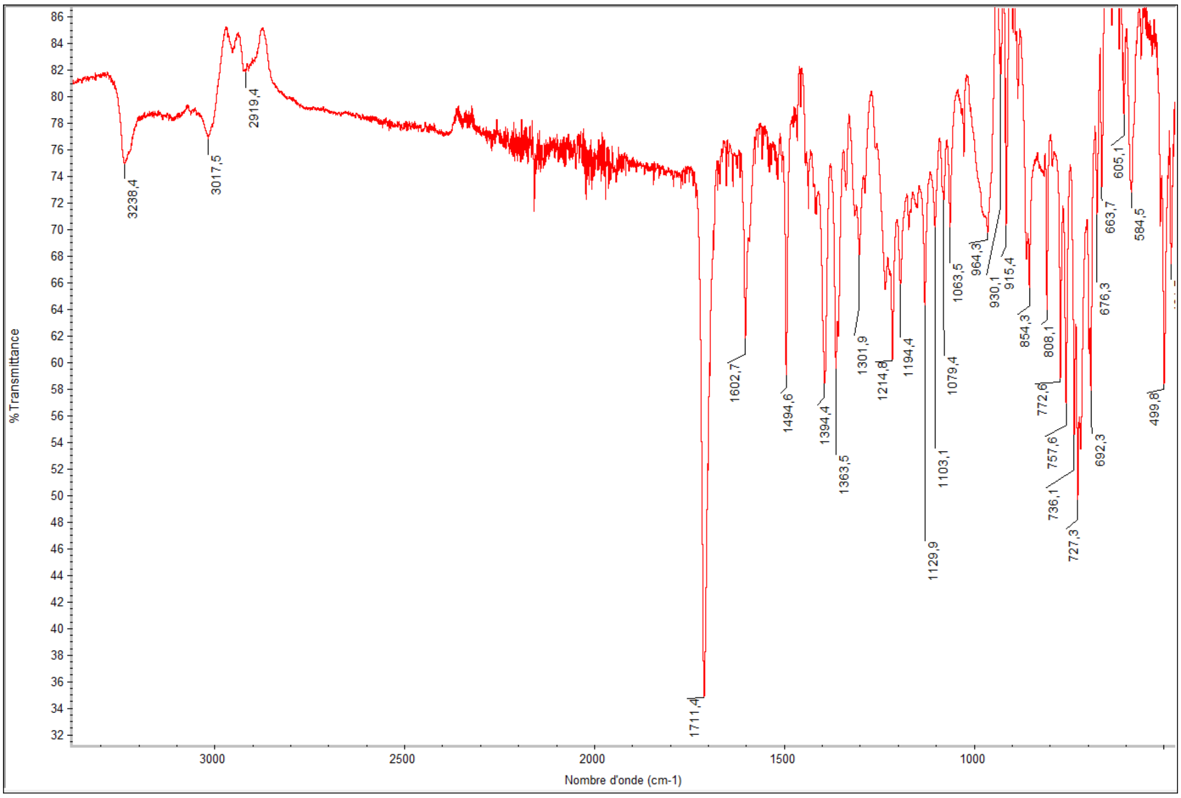

FT-IR spectrum of the compound **5f**

3-(Mercaptomethyl)-1-(phenylamino)-1*H*-imidazo[2,1-*a*]isoindole-2,5(3*H*,9b*H*)-dione (5g)

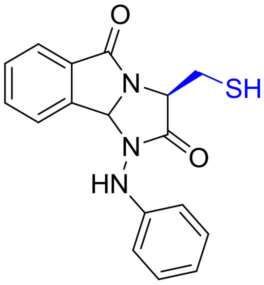

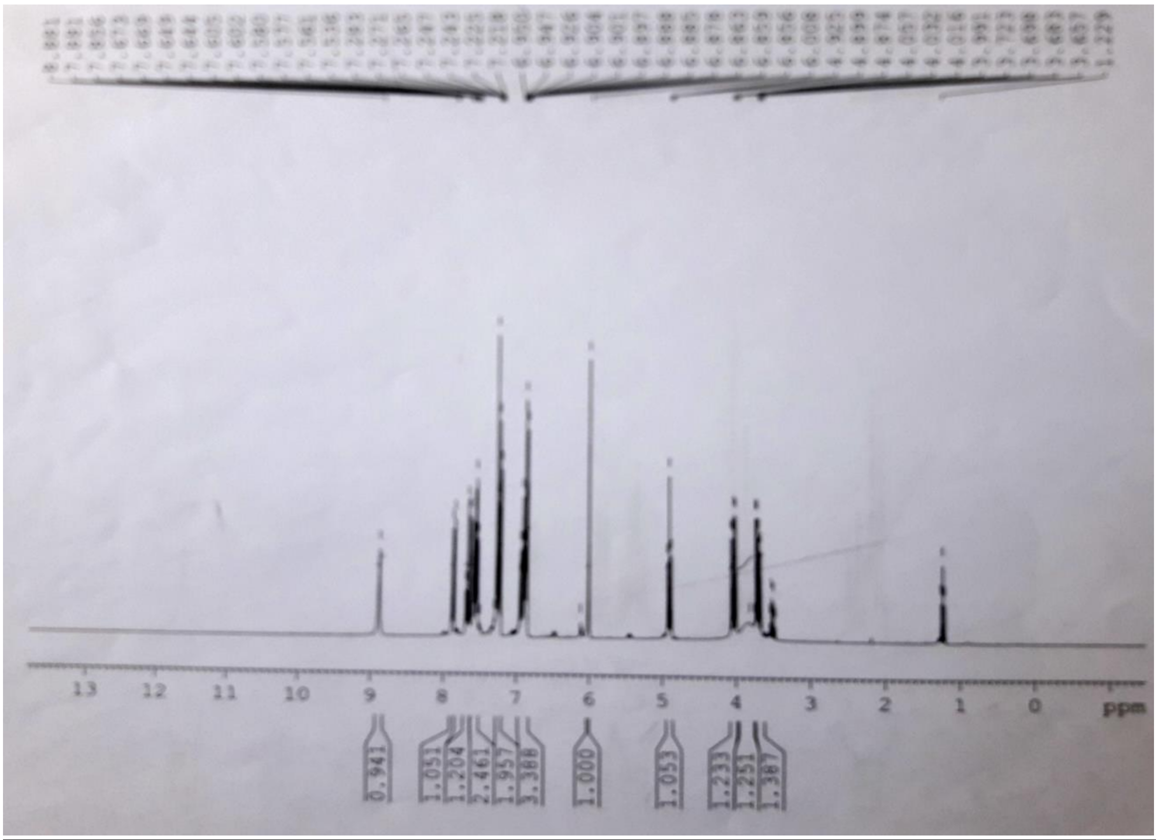

^{1}H NMRspectrum of the compound **5g** in $CDCl_3$ at 300 MHz

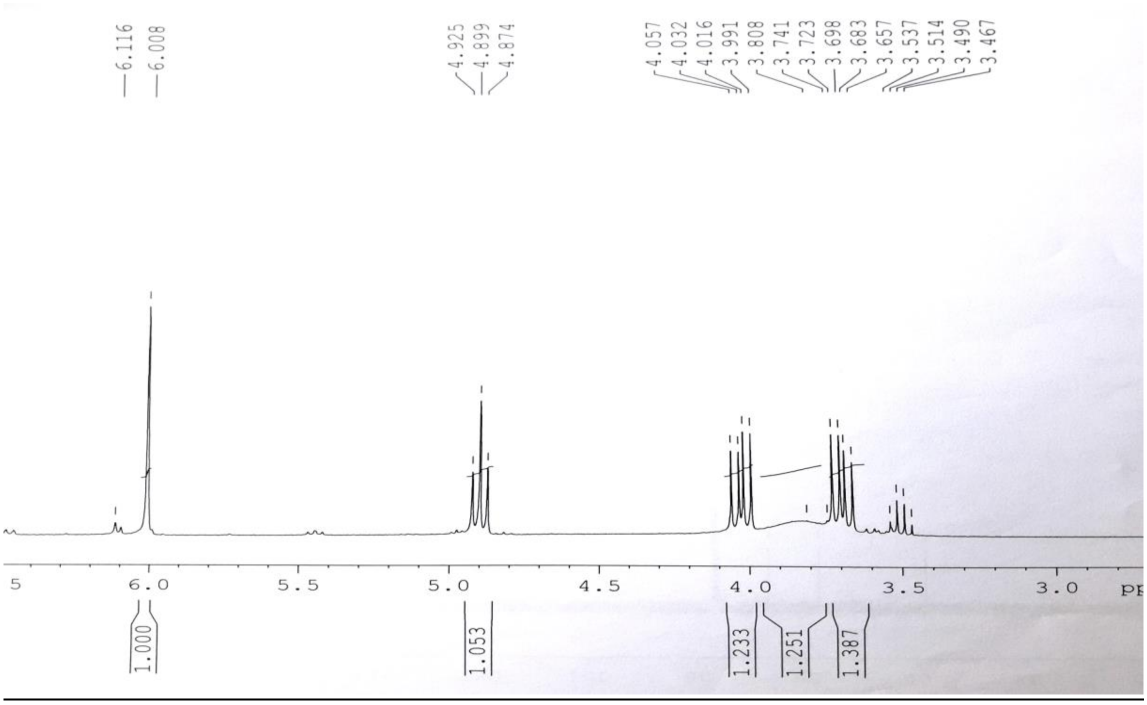

^{1}H NMRspectrum of the compound **5g** in $CDCl_3$ at 300 MH

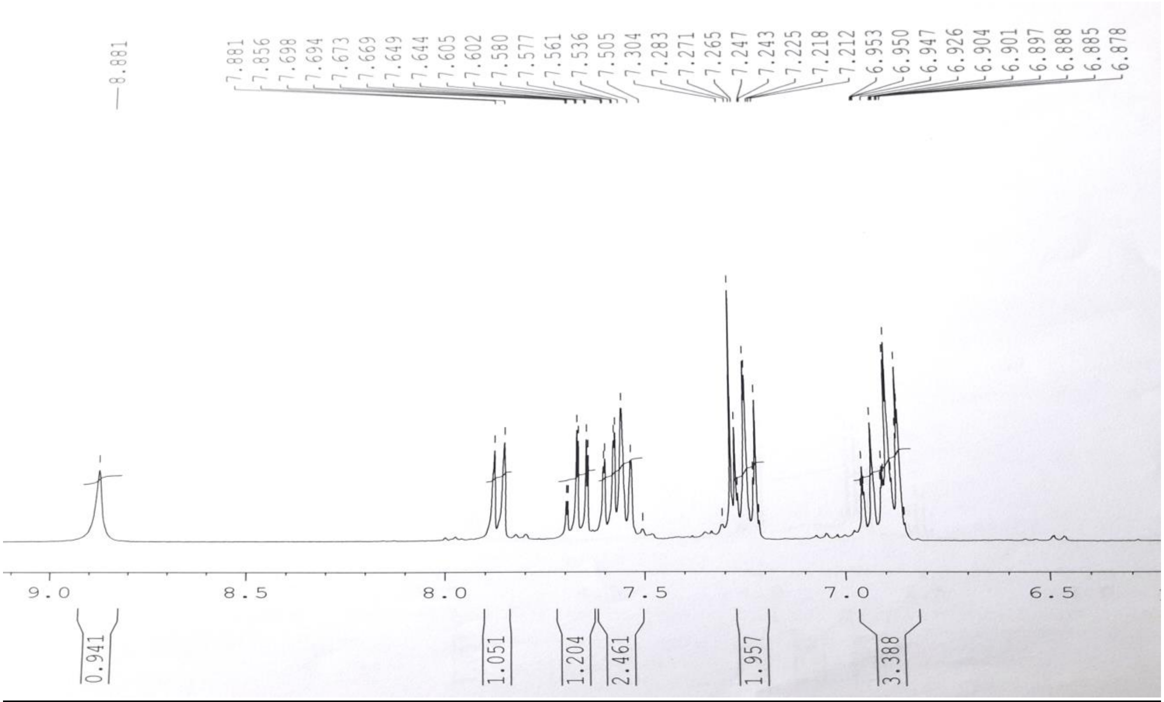

^{1}H NMRspectrum of the compound **5g** in $CDCl_3$ at 300 MHz

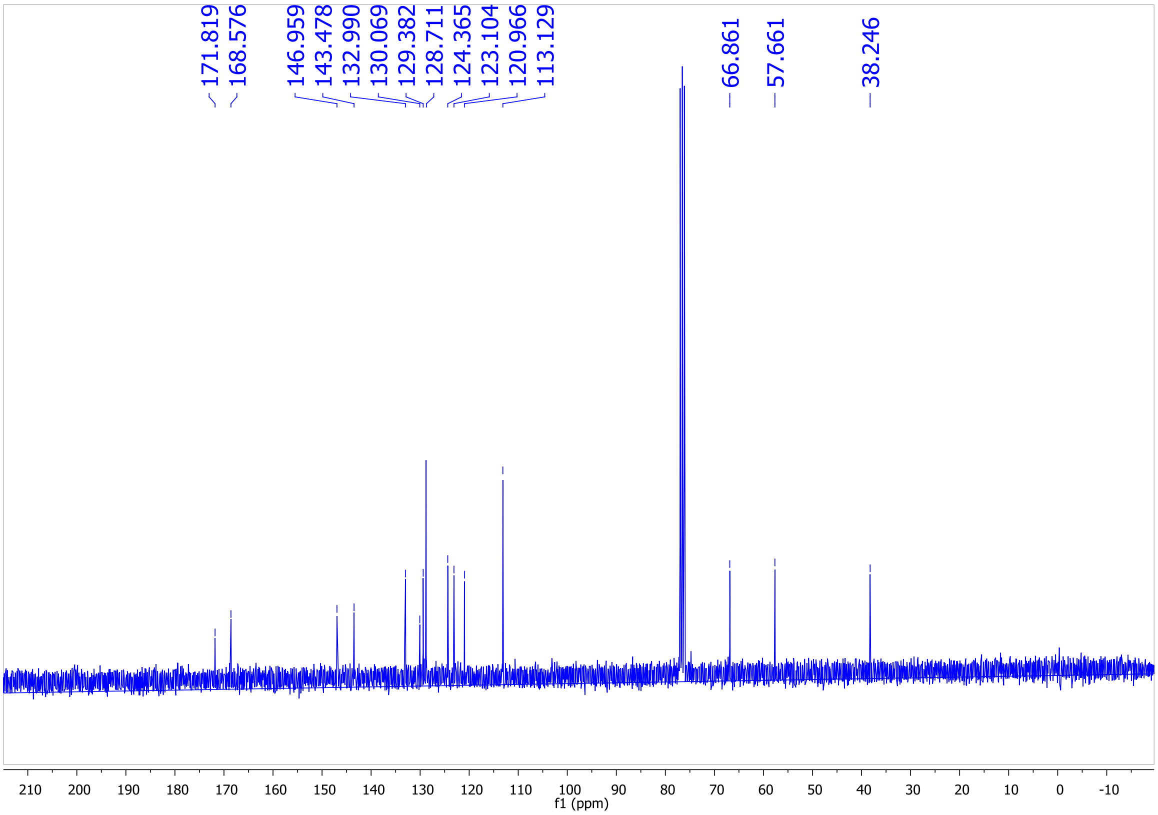

^{13}C NMRspectrum of the compound **5g** in $CDCl_3$ at 75 MHz

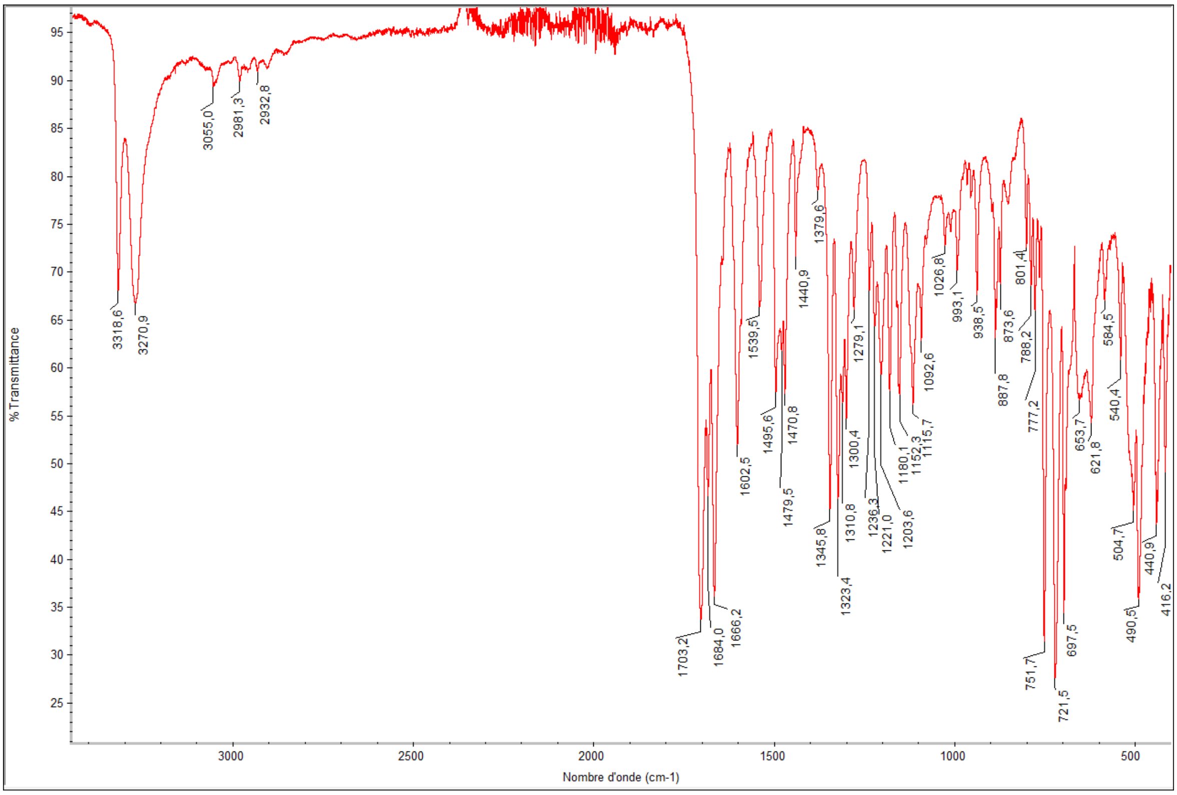

FT-IR spectrum of the compound **5g**

3-(Hydroxymethyl)-1-(phenylamino)-1*H*-imidazo[2,1-*a*]isoindole-2,5(3*H*,9b*H*)-dione (5h)

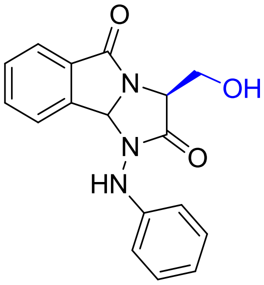

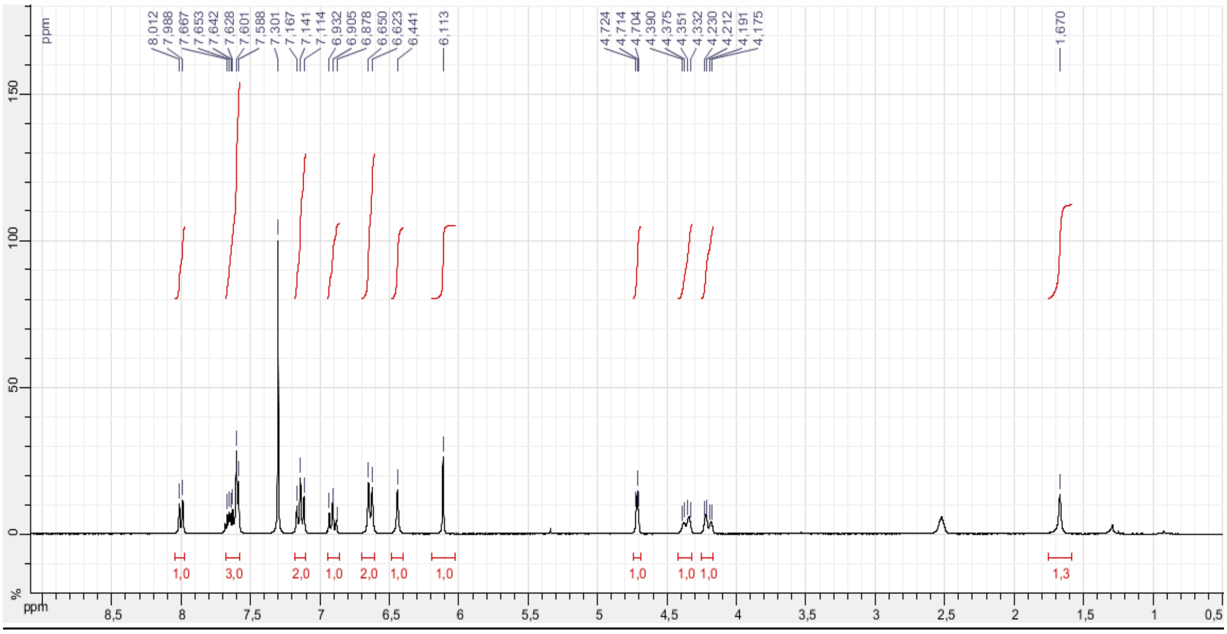

^{1}H NMRspectrum of the compound **5h** in $CDCl_3$ at 300 MHz

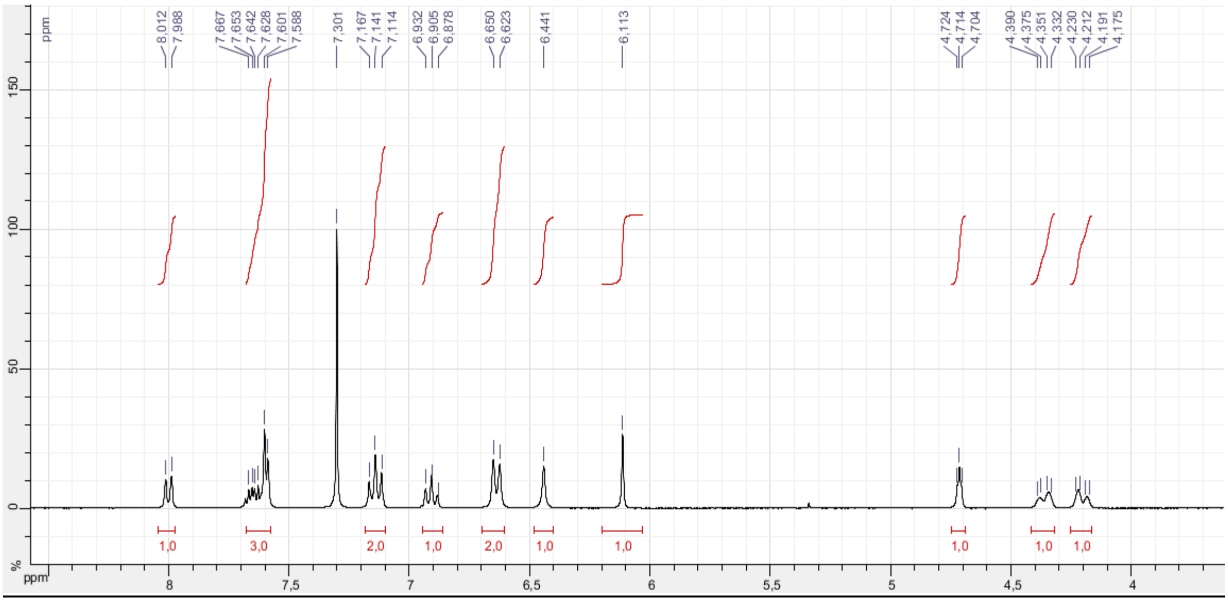

^{1}H NMRspectrum of the compound **5h** in $CDCl_3$ at 300 MHz

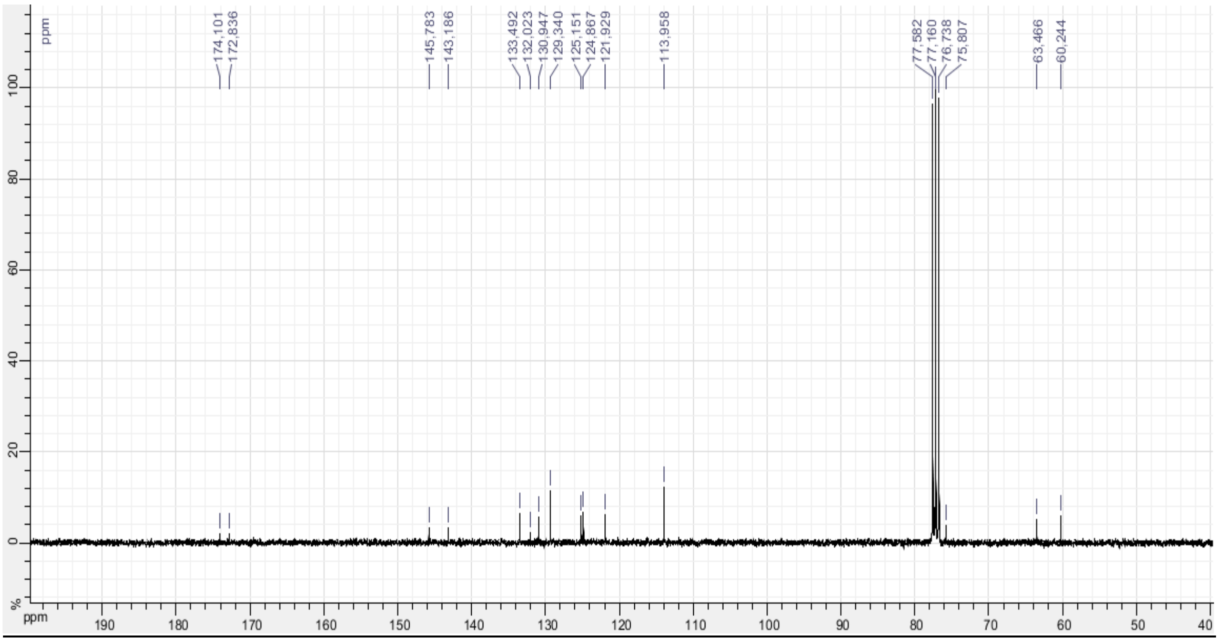

^{13}C NMRspectrum of the compound **5h** in $CDCl_3$ at 75 MHz

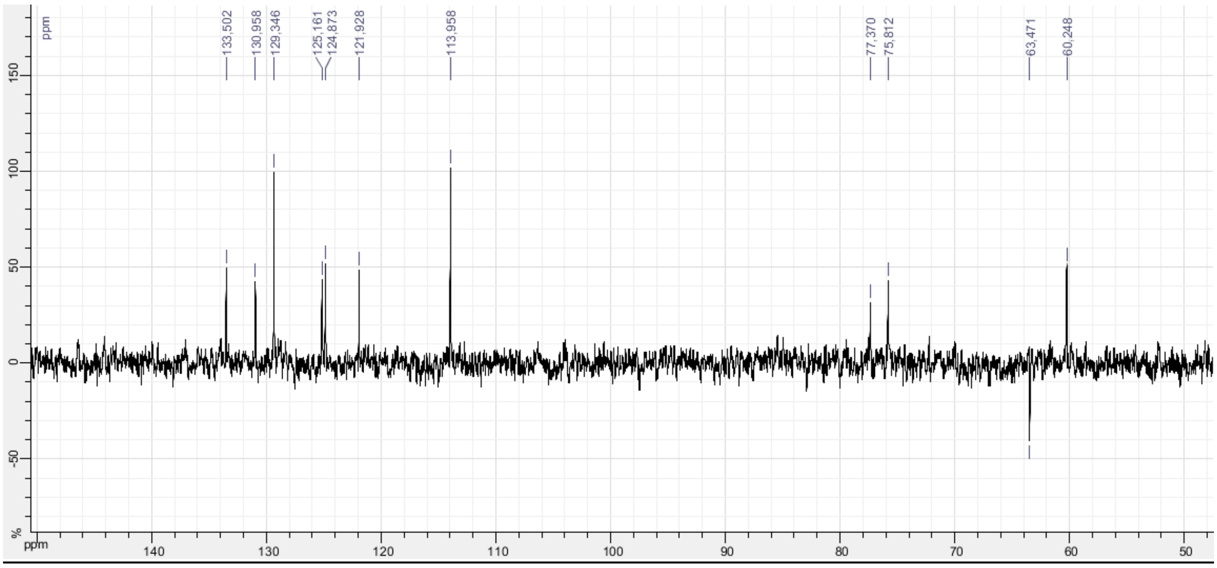

DEPT 135 NMR spectrum of the compound **5h** in $CDCl_3$ at 75 MHz

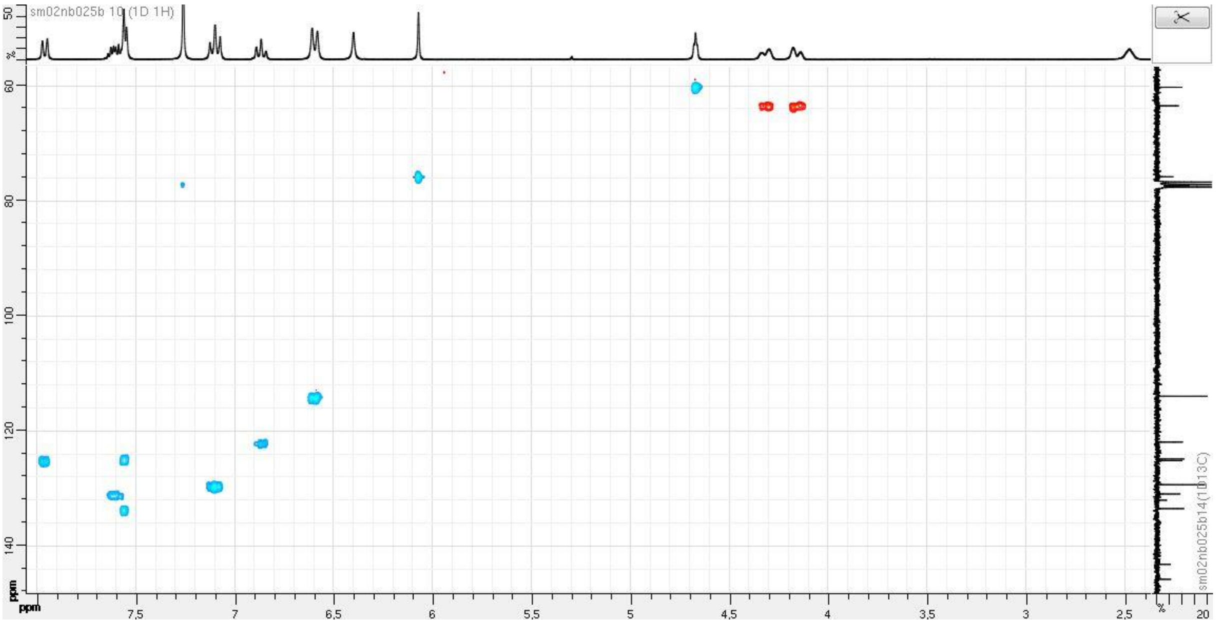

HSQC NMR spectrum of the compound **5h** in $CDCl_3$

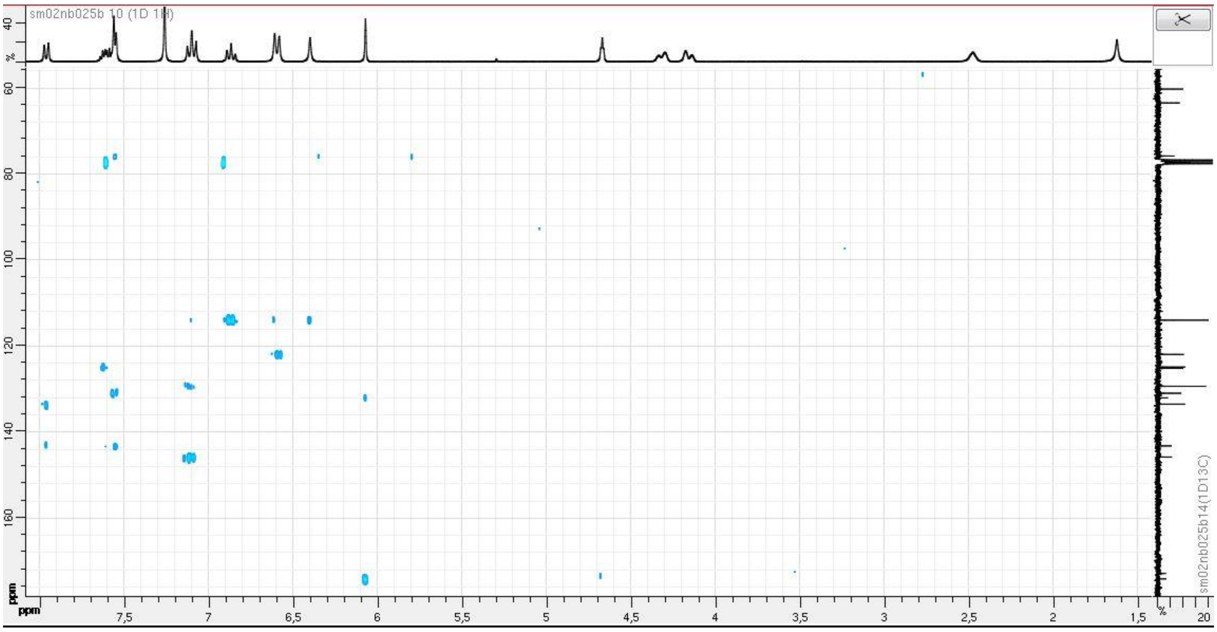

HMBC NMR spectrum of the compound **5h** in $CDCl_3$

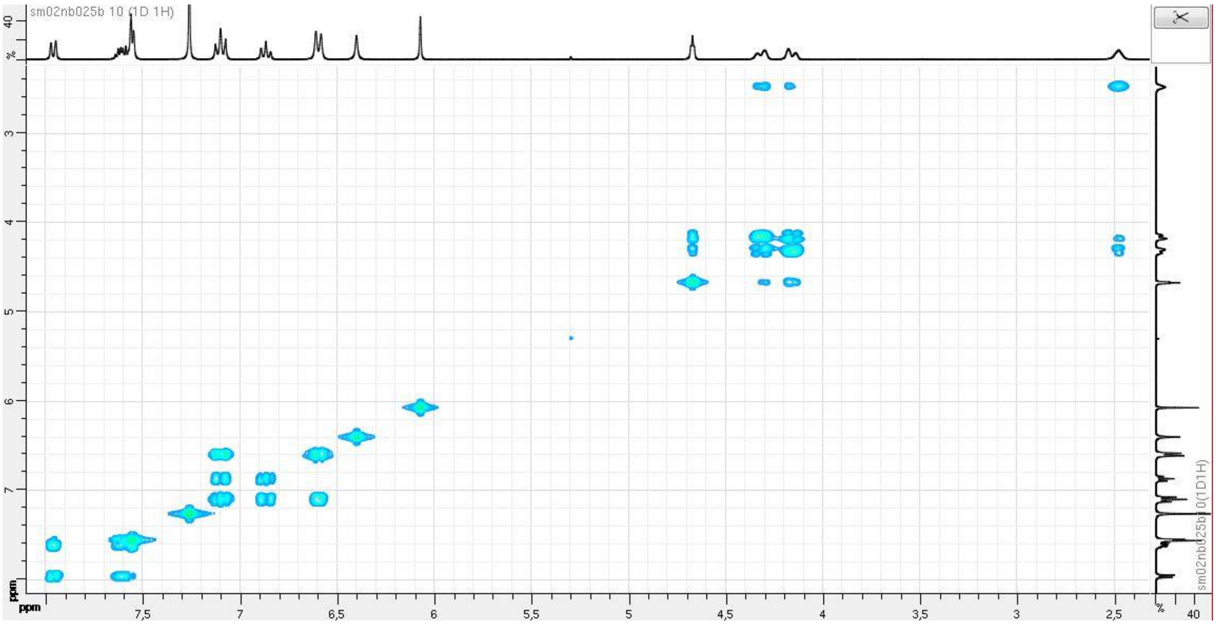

COSY NMR spectrum of the compound **5h** in $CDCl_3$

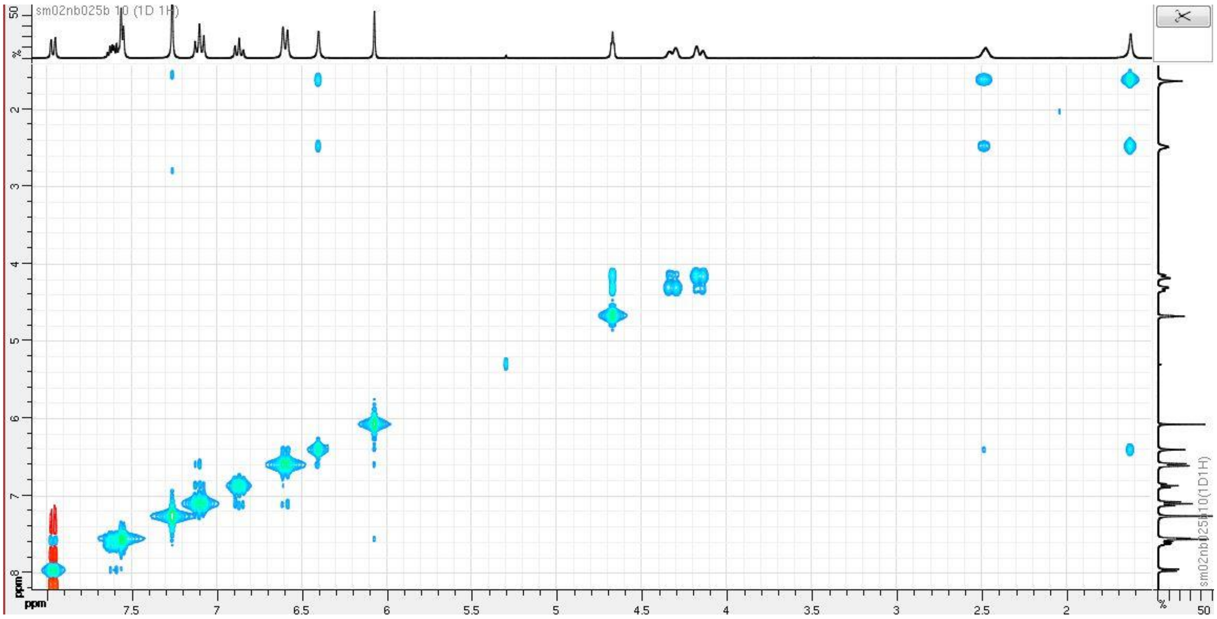

NOESY NMR spectrum of the compound **5h** in $CDCl_3$

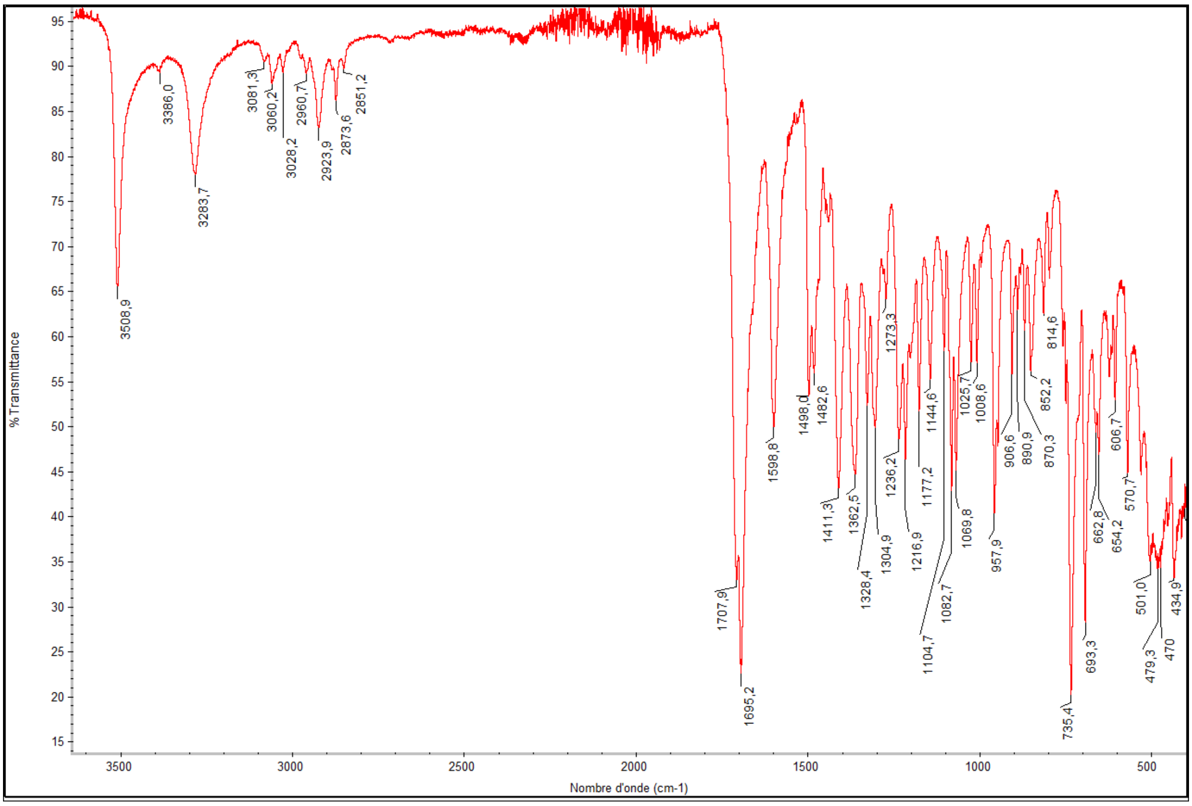

FT-IR spectrum of the compound **5h**

3-((1*H*-Indol-3-yl)methyl)-1-(phenylamino)-1*H*-imidazo[2,1-*a*]isoindole-2,5(3*H*,9b*H*)dione (5i)

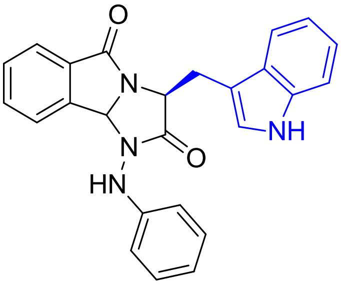

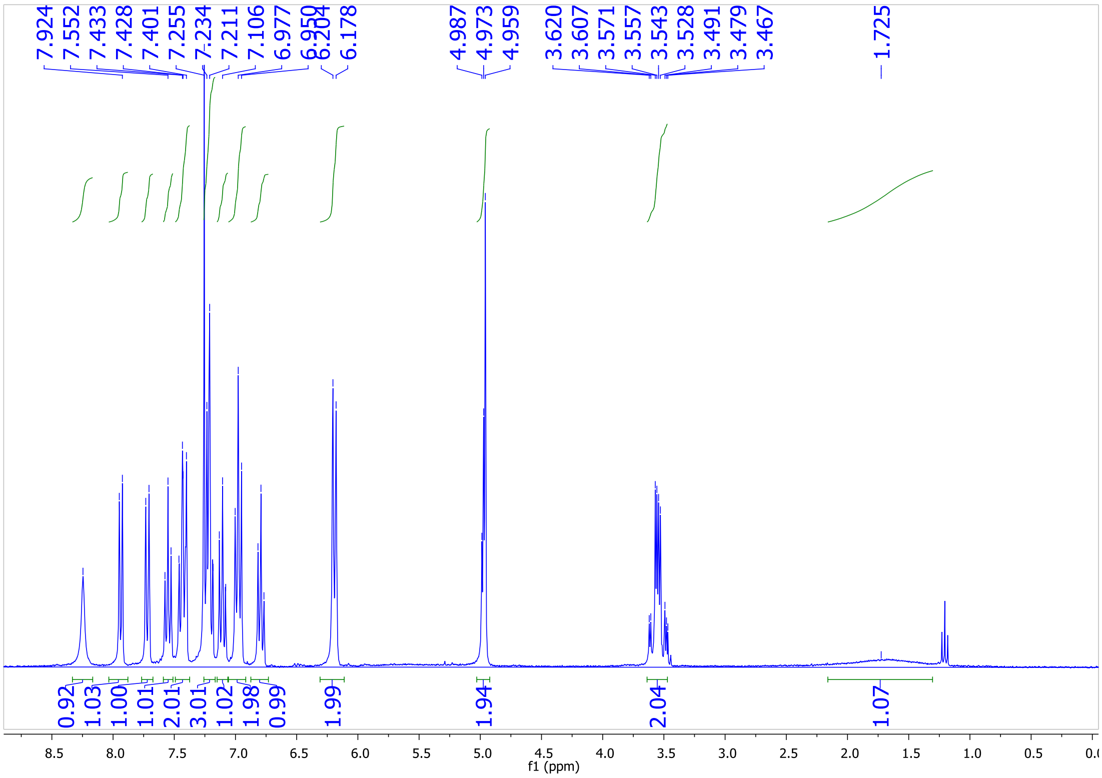

^{1}H NMRspectrum of the compound **5i** in $CDCl_3$ at 300 MHz

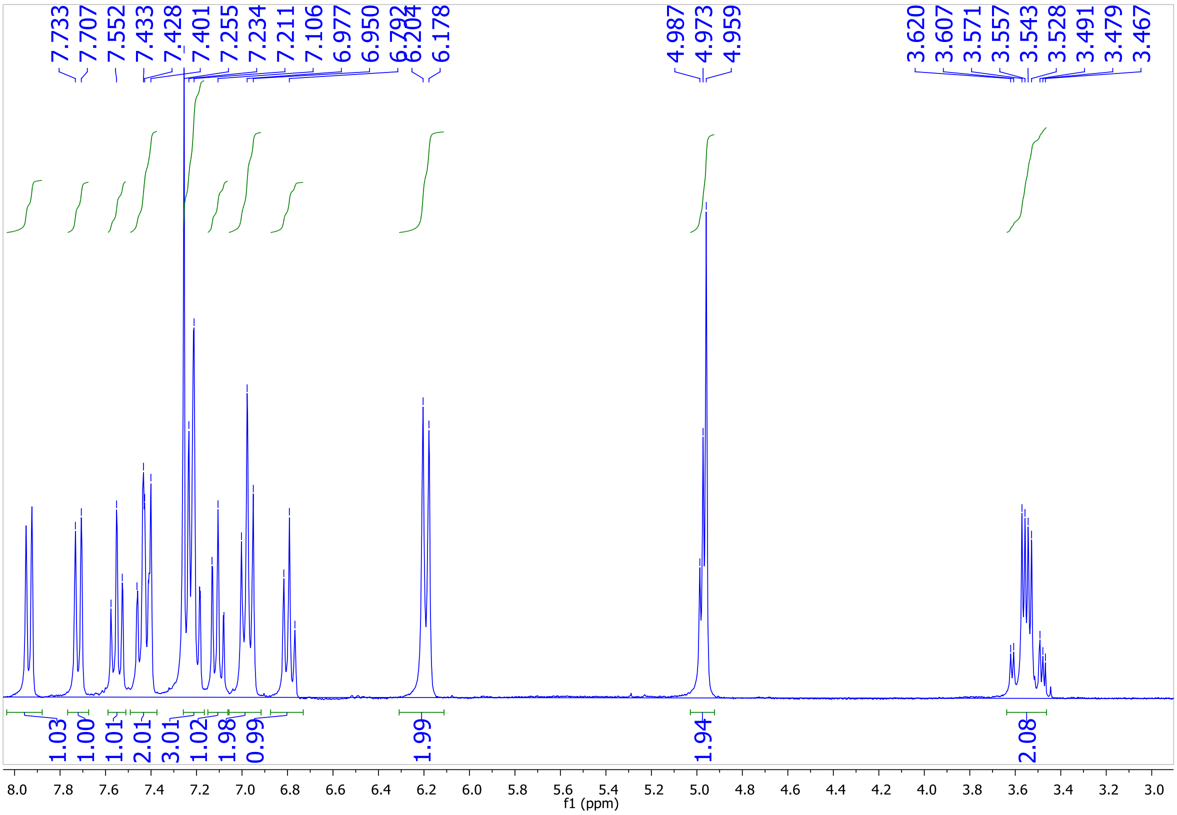

^{1}H NMRspectrum of the compound **5i** in $CDCl_3$ at 300 MHz

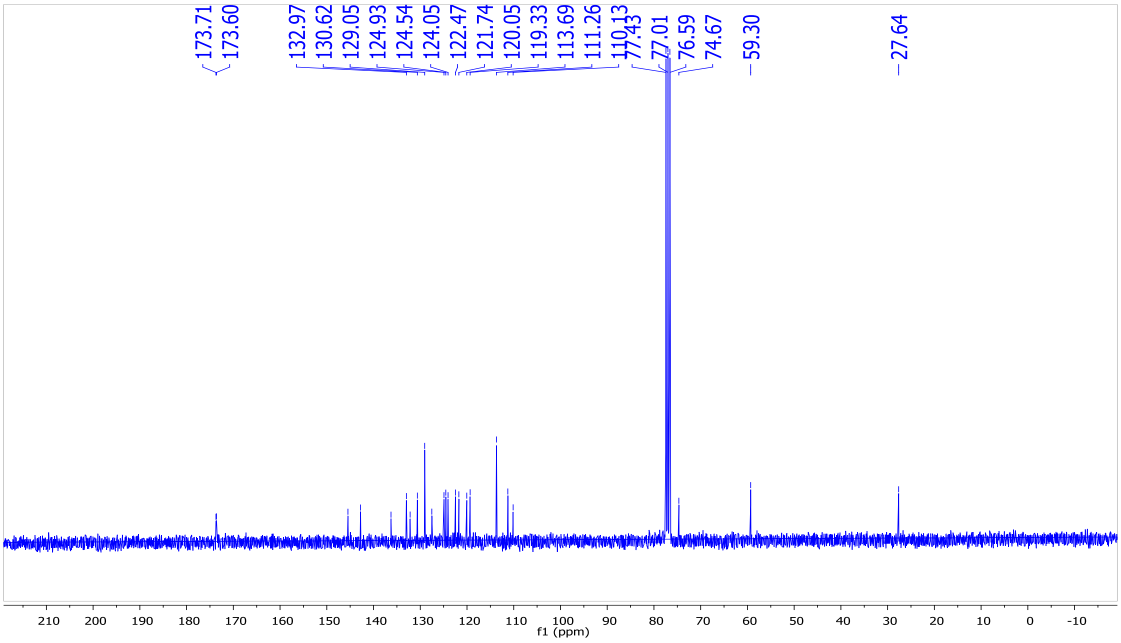

^{13}C NMRspectrum of the compound **5i** in $CDCl_3$ at 75 MHz

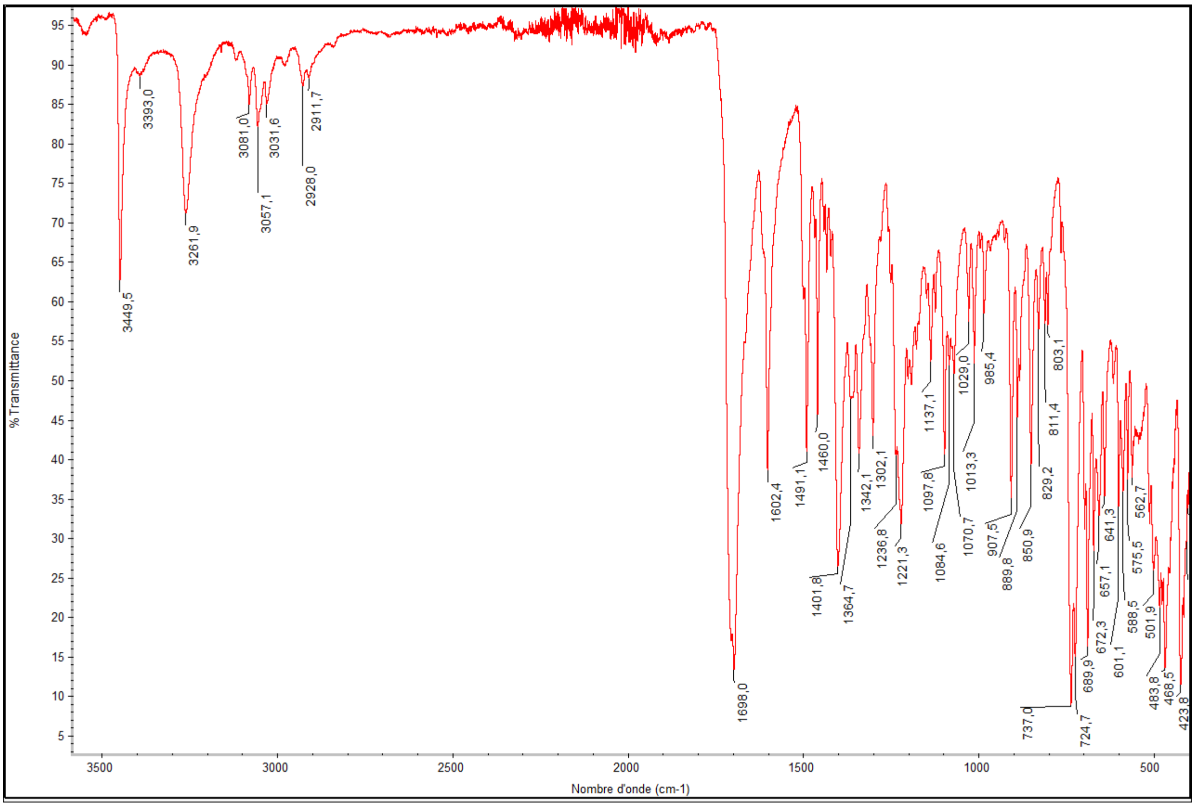

FT-IR spectrum of the compound **5i**

3-(4-Hydroxybenzyl)-1-(phenylamino)-1*H*-imidazo[2,1-*a*]isoindole-2,5(3*H*,9b*H*)-dione (5j)

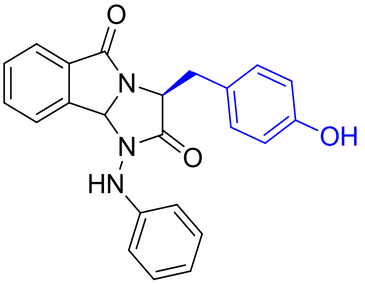

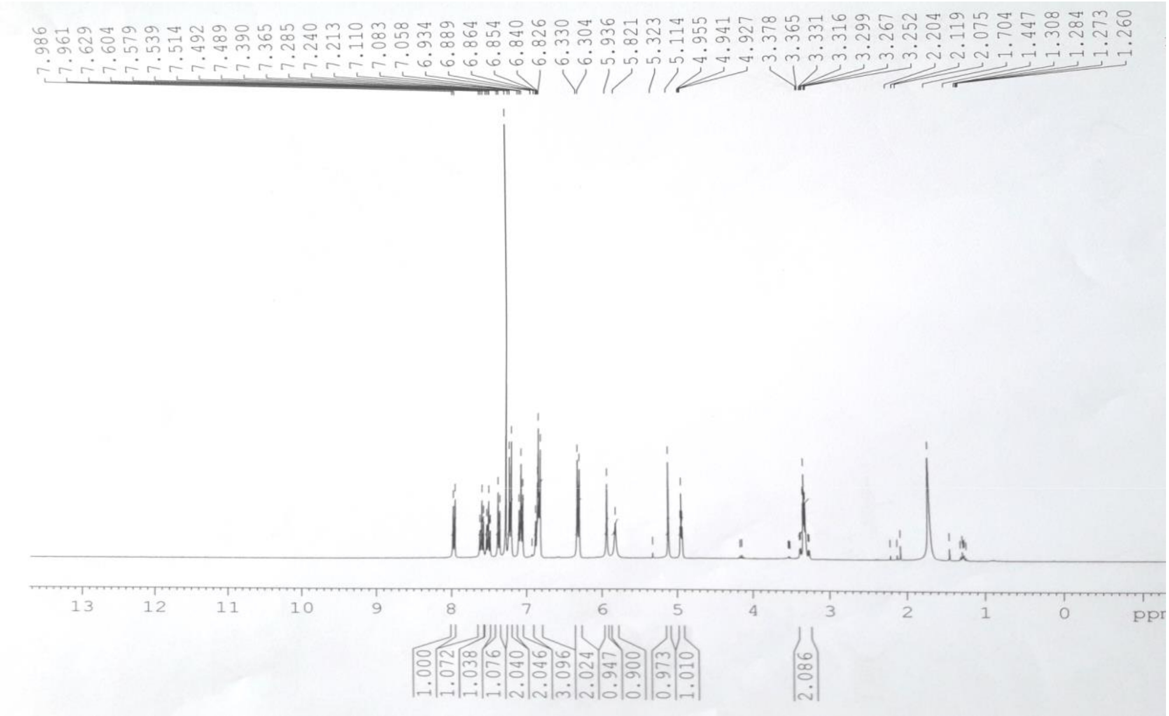

^{1}H NMRspectrum of the compound **5j** in $CDCl_3$ at 300 MHz

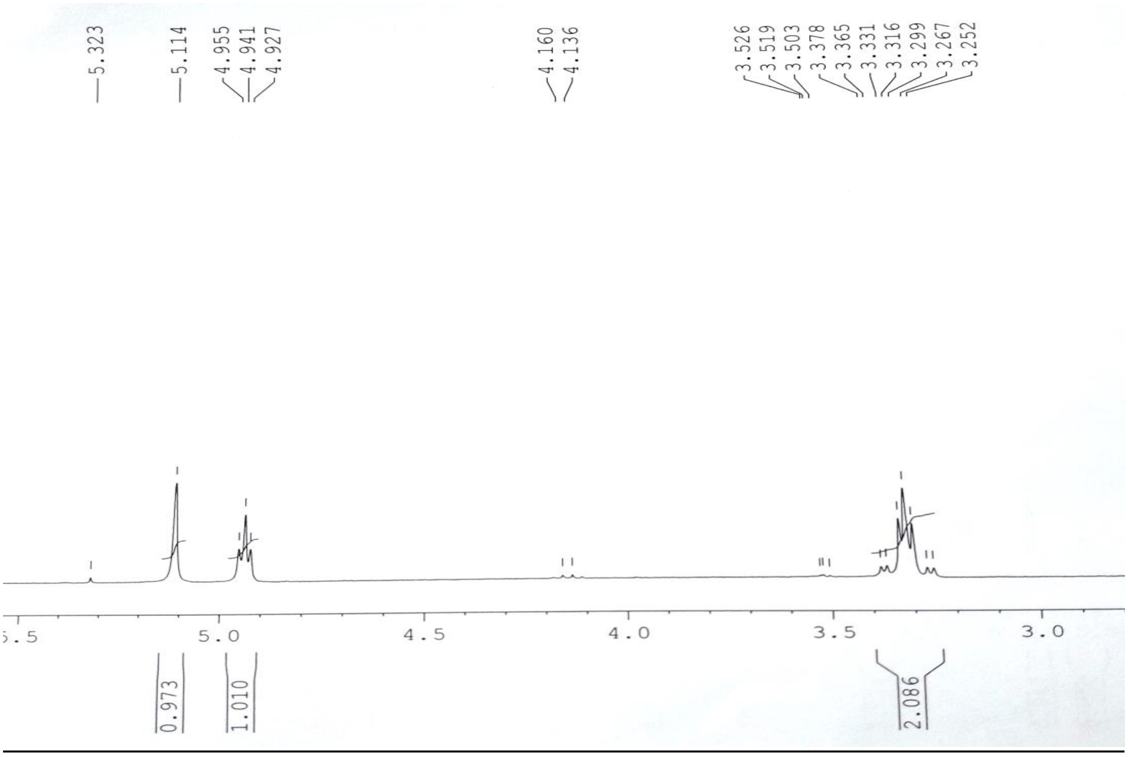

^{1}H NMRspectrum of the compound **5j** in $CDCl_3$ at 300 MHz

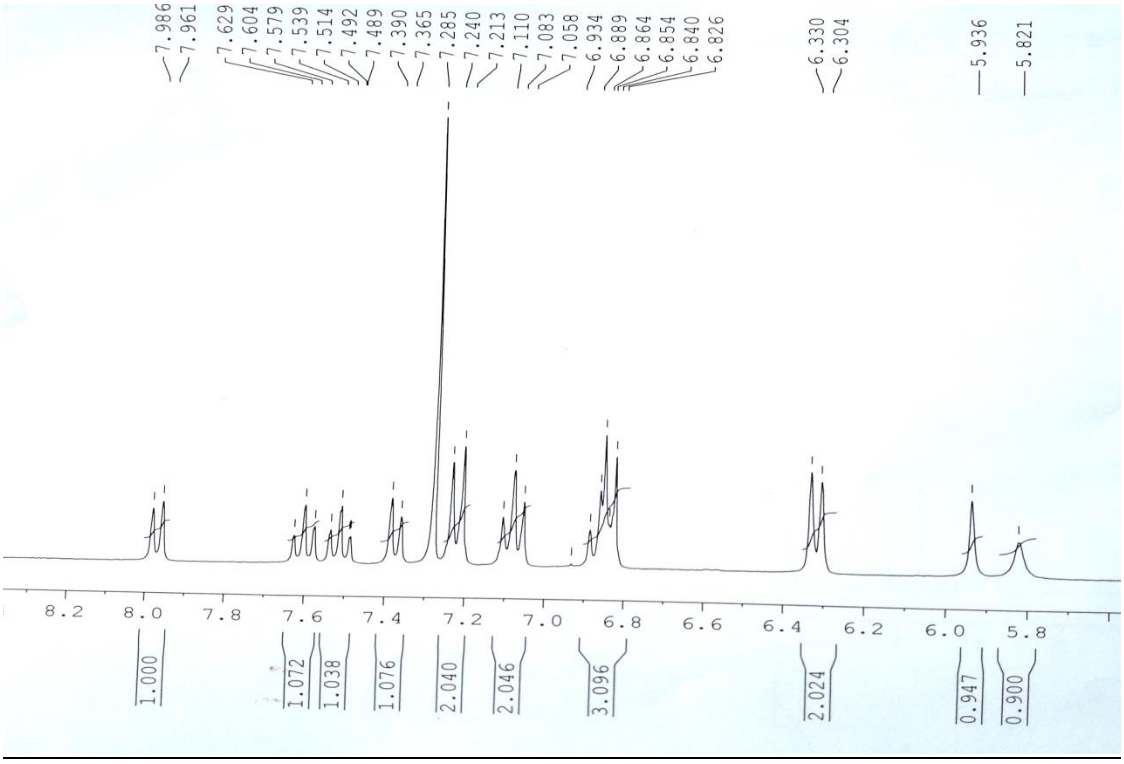

^{1}H NMRspectrum of the compound **5j** in $CDCl_3$ at 300 MHz

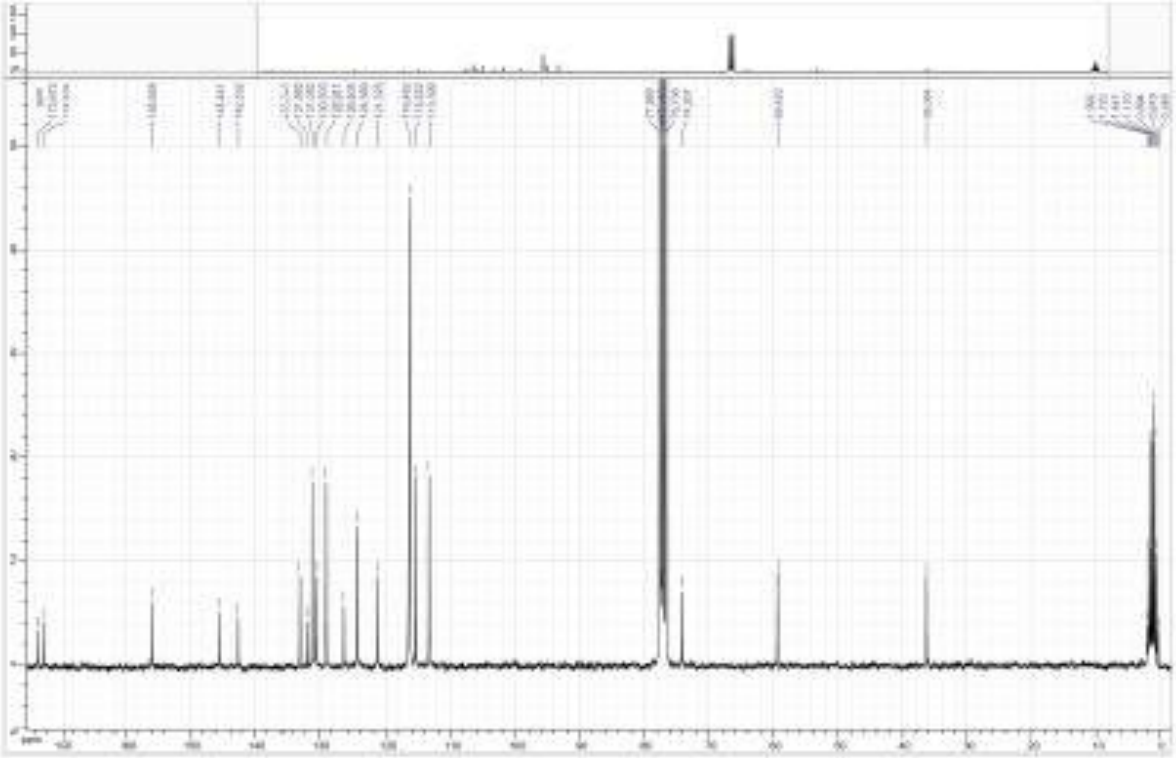

^{13}C NMRspectrum of the compound **5j** in $CDCl_3$ + CD_3CN at 75 MHz

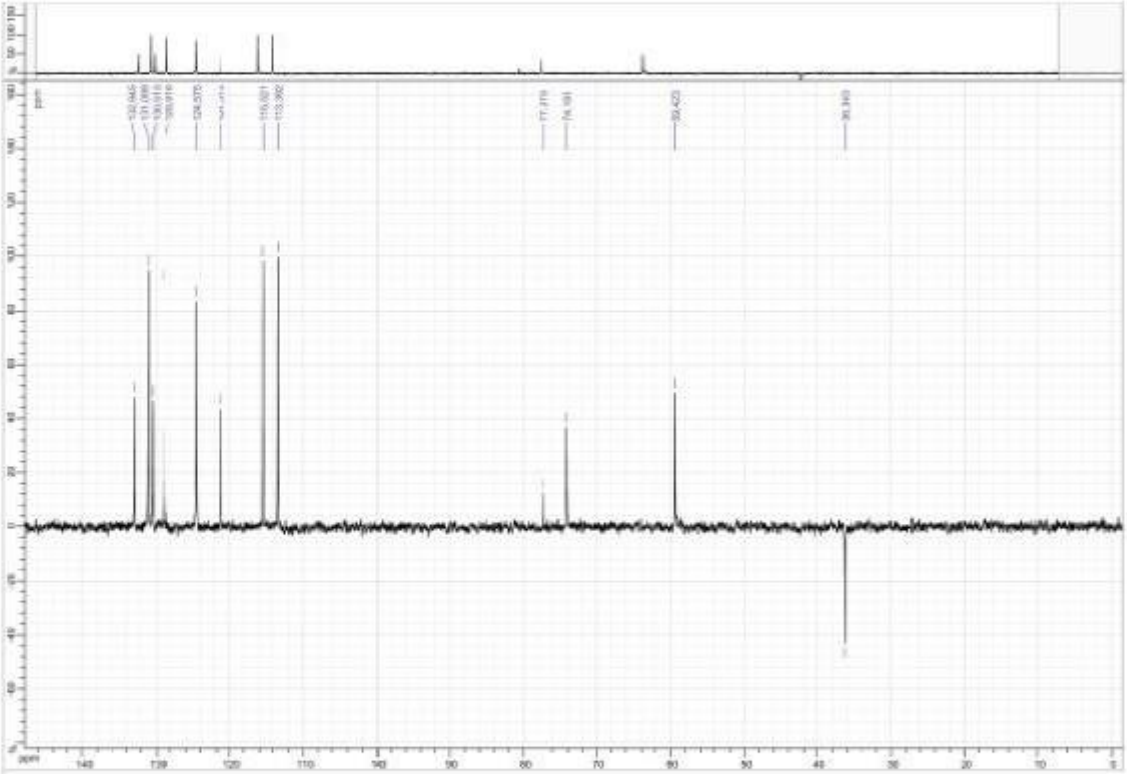

DEPT 135 NMR spectrum of the compound **5j** in $CDCl_3$ + CD_3CN at 75 MHz

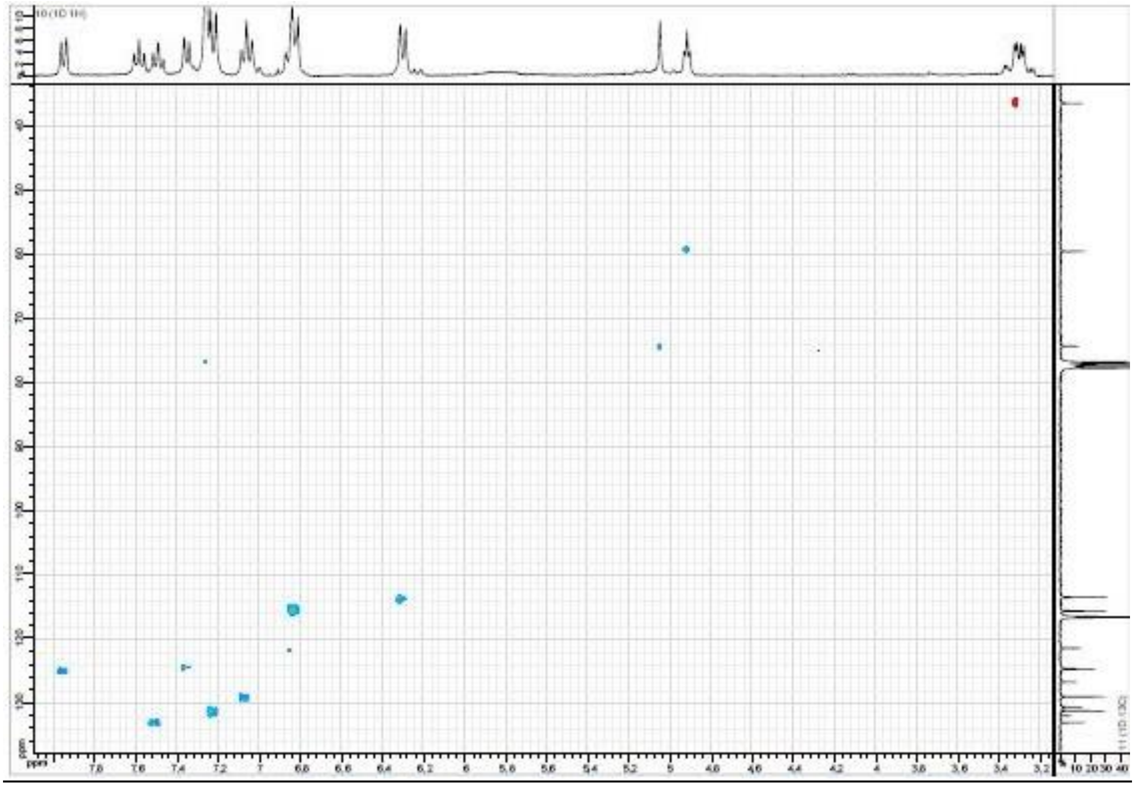

HSQC NMR spectrum of the compound **5j** in $CDCl_3$

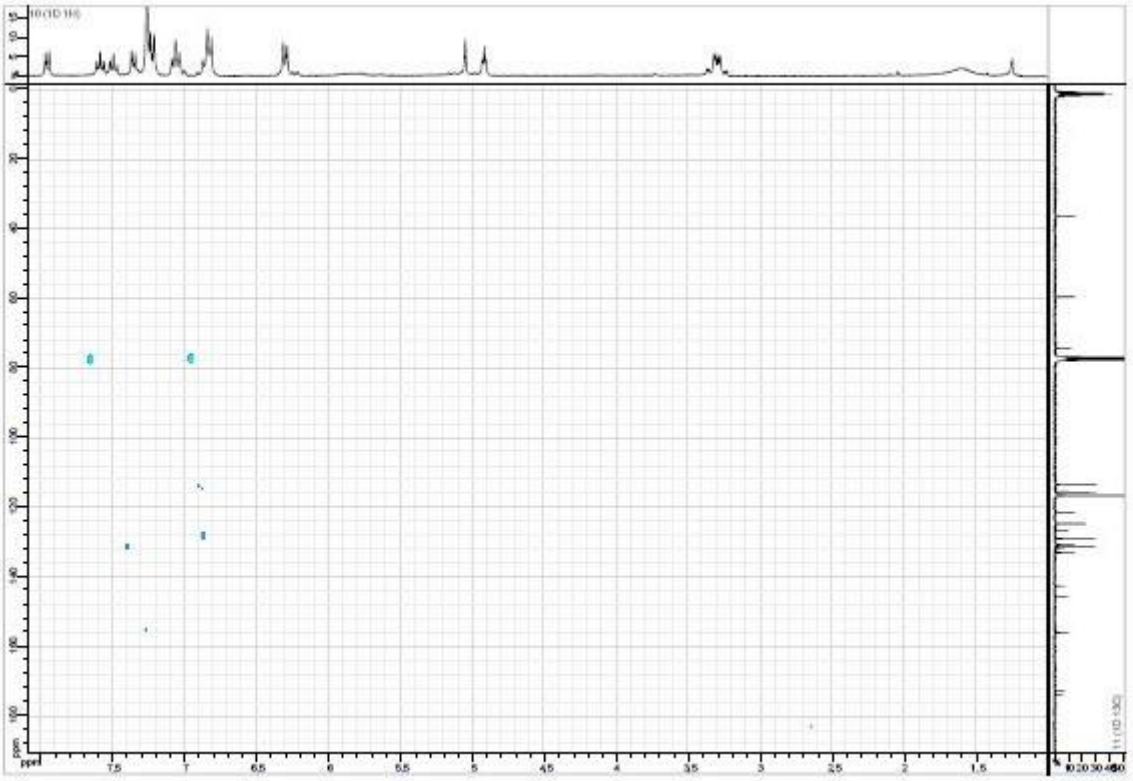

HMBC NMR spectrum of the compound **5j** in $CDCl_3$

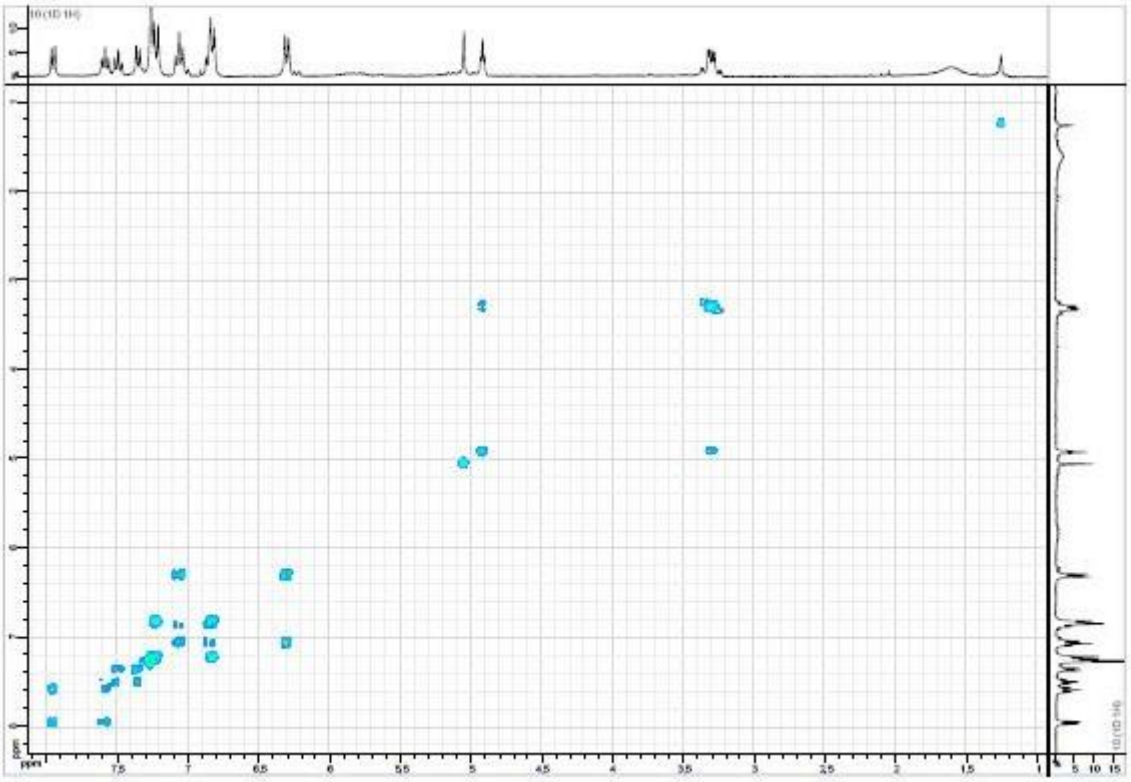

COSY NMR spectrum of the compound **5j** in $CDCl_3$

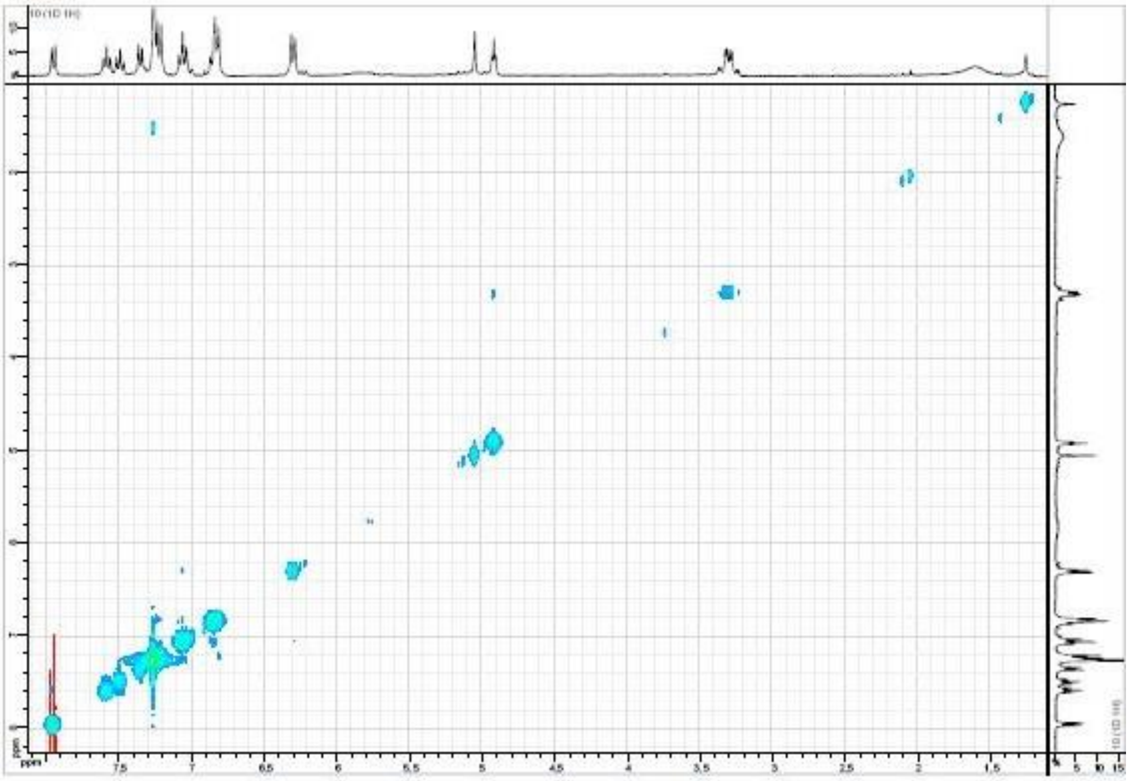

NOESY NMR spectrum of the compound **5j** in $CDCl_3$

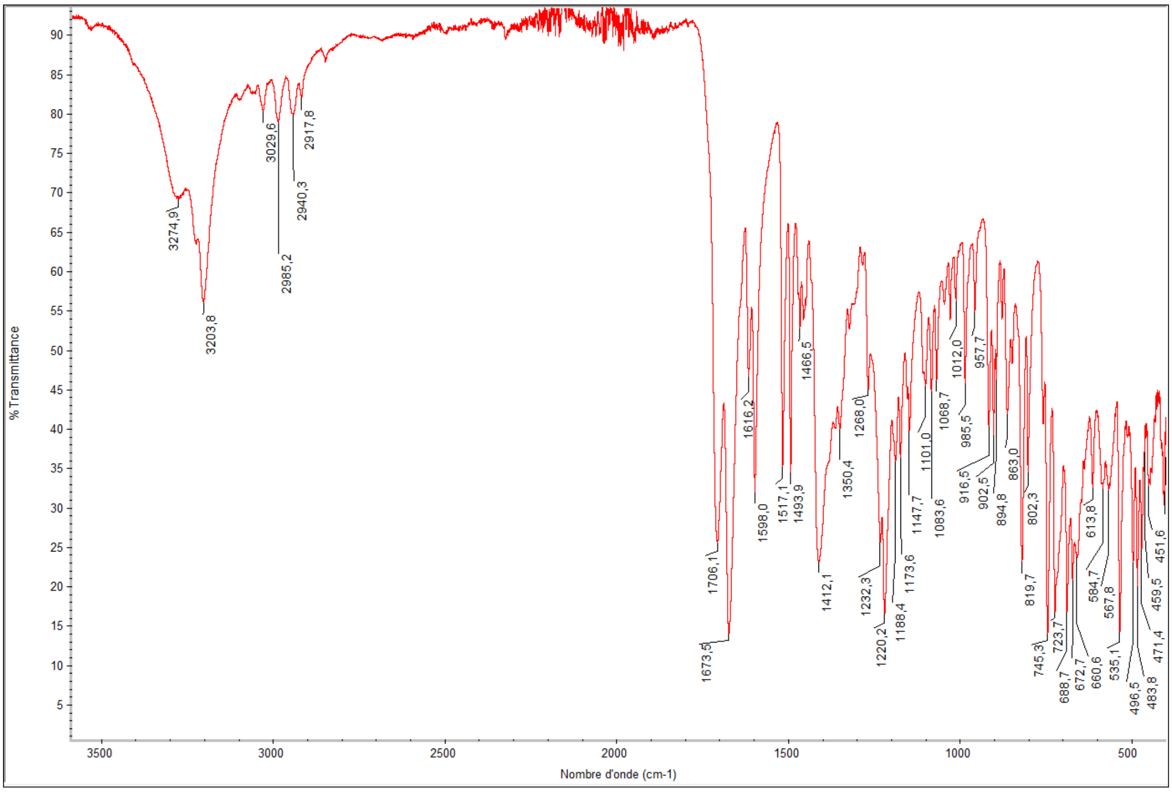

FT-IR spectrum of the compound **5j**

1-(4-Chlorophenylamino)-3-methyl-1*H*-imidazo[2,1-*a*]isoindole-2,5(3*H*,9b*H*)-dione (5k)

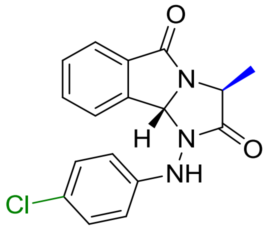

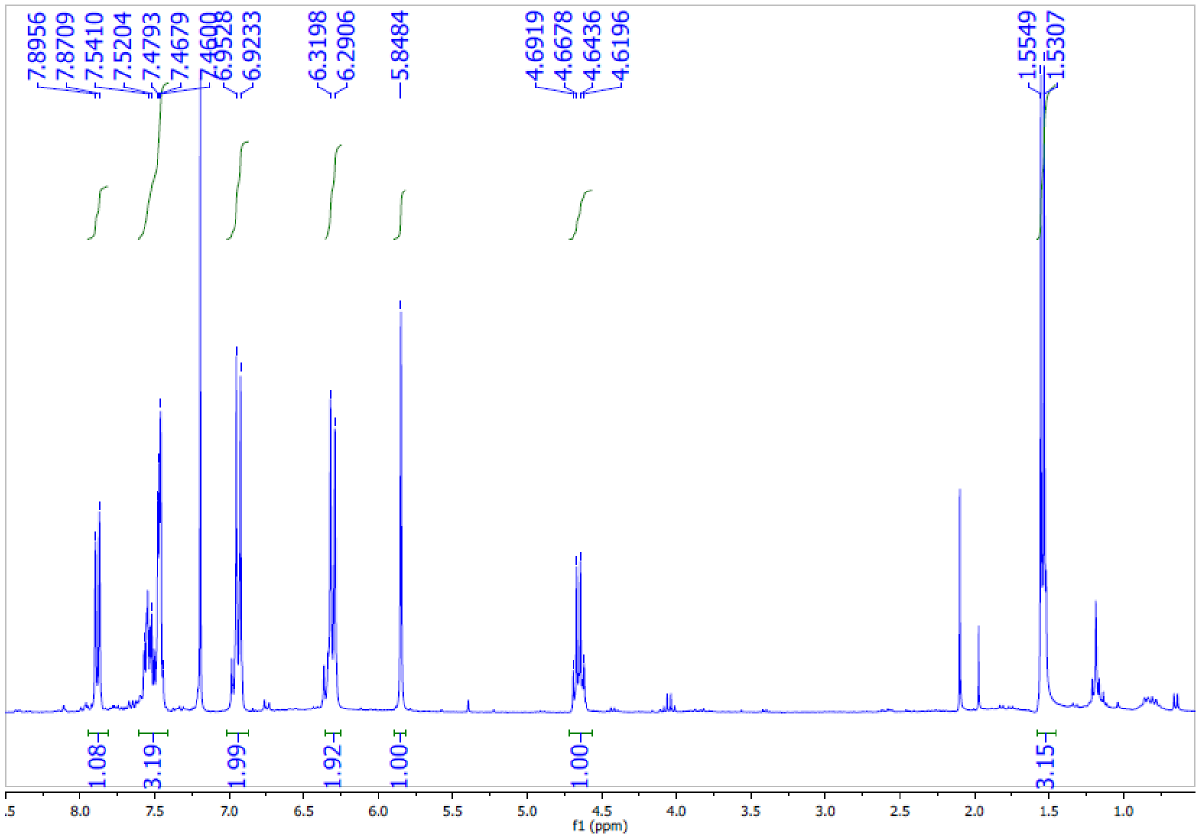

^{1}H NMRspectrum of the compound **5k** in $CDCl_3$ at 300 MHz

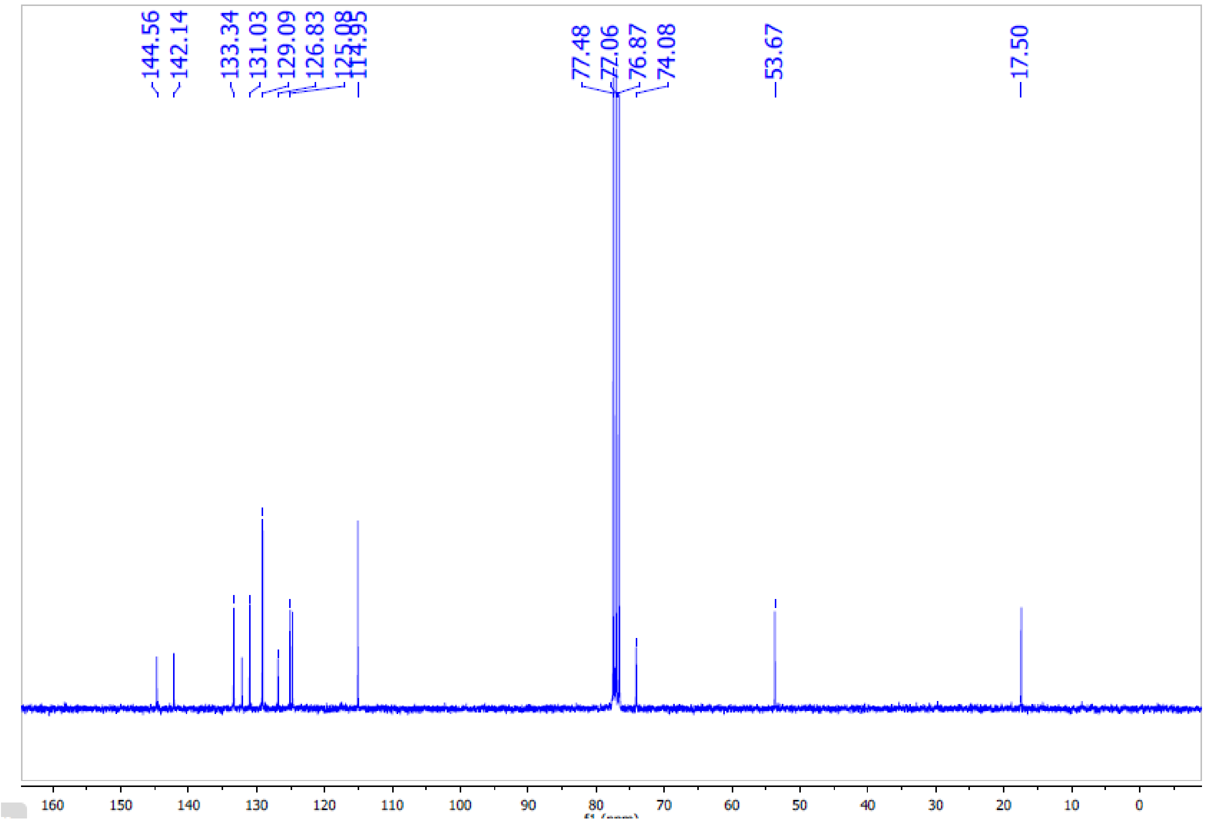

^{13}C NMRspectrum of the compound **5k** in $CDCl_3$ at 75 MHz

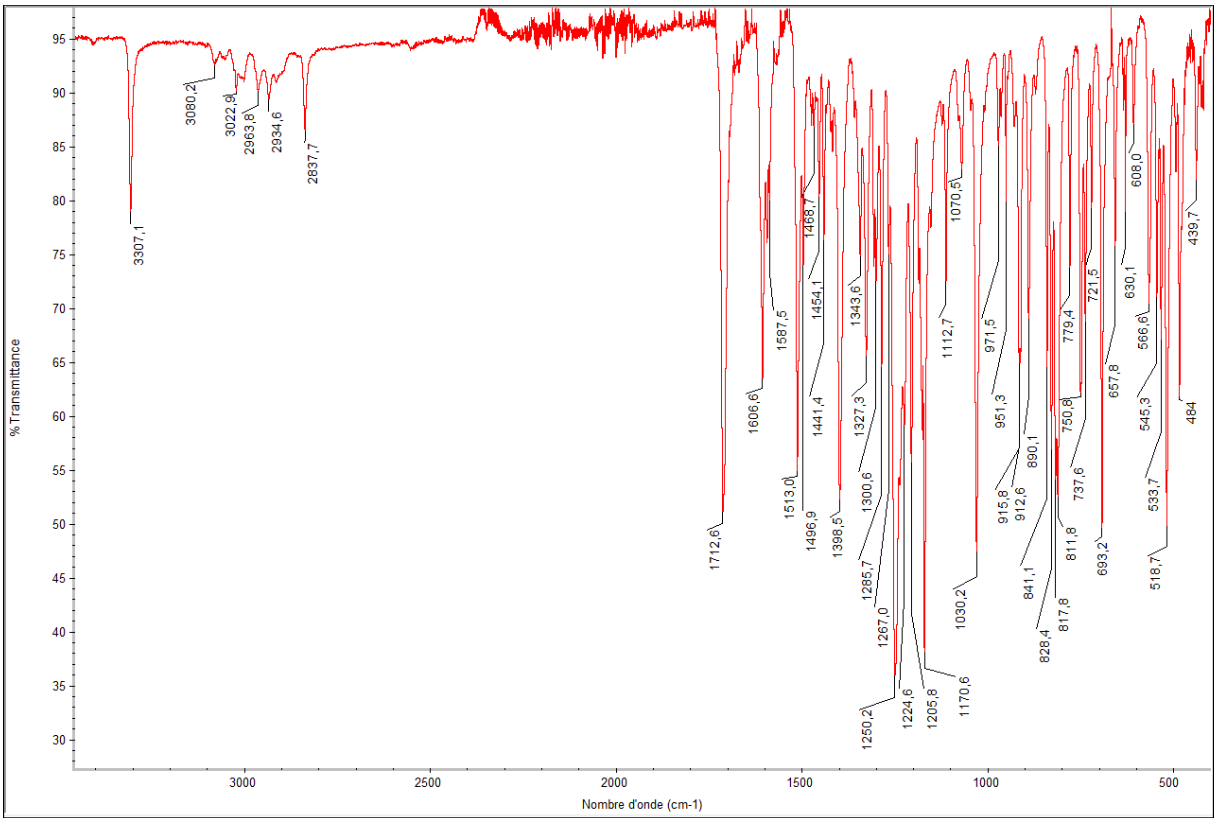

FT-IR spectrum of the compound **5k**

1-(4-Chlorophenylamino)-3-phenyl-1*H*-imidazo[2,1-*a*]isoindole-2,5(3*H*,9b*H*)-dione (5l)

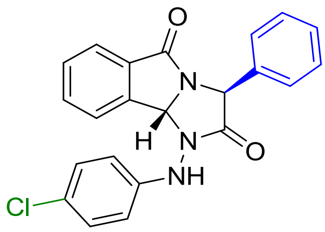

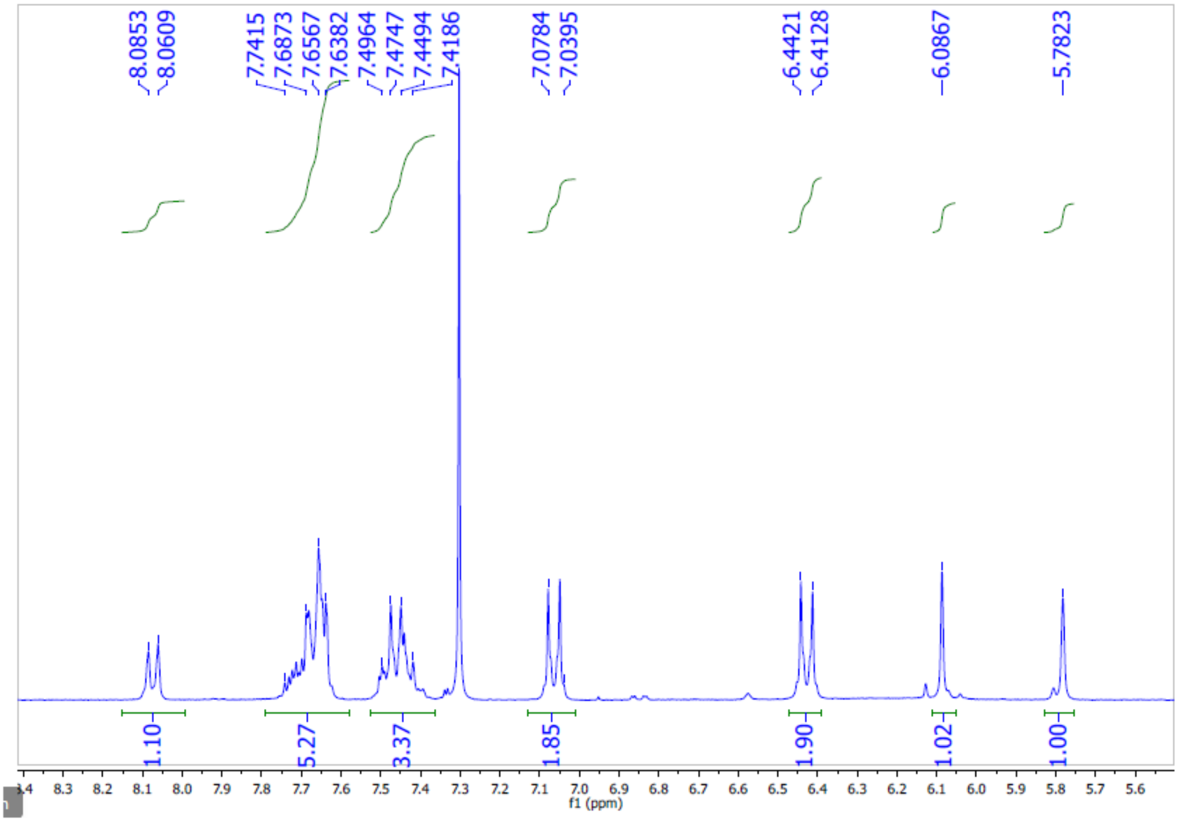

^{1}H NMRspectrum of the compound **5l** in $CDCl_3$ at 300 MHz

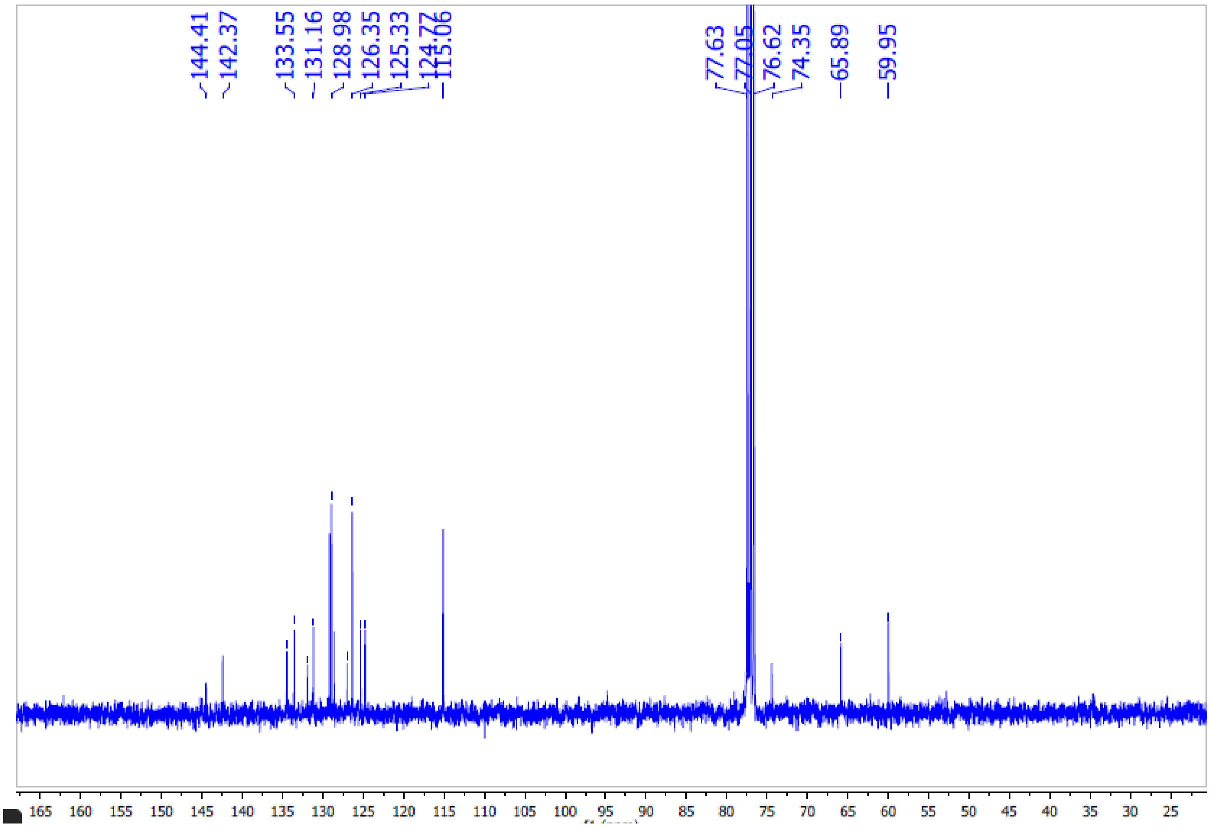

^{13}C NMRspectrum of the compound **5l** in $CDCl_3$ at 75 MHz

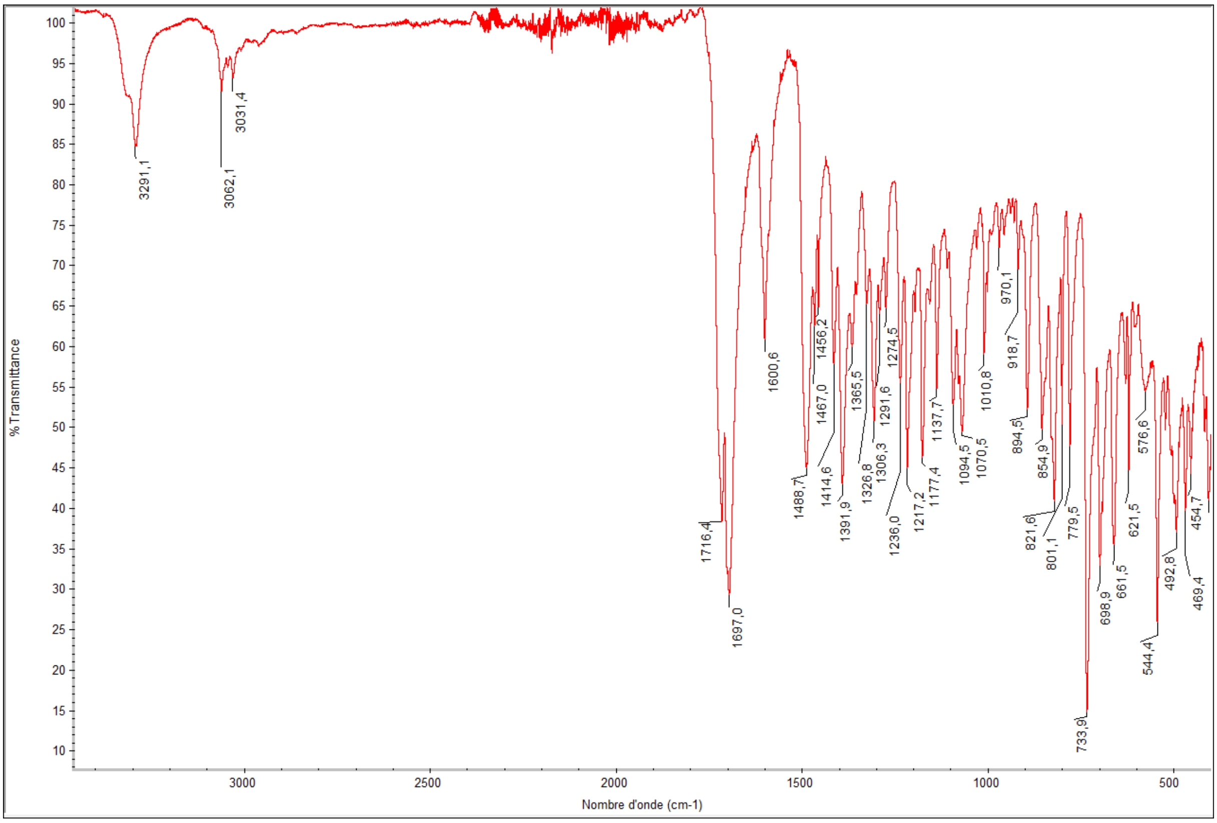

FT-IR spectrum of the compound **5l**

3-Benzyl-1-(4-chlorophenylamino)-1*H*-imidazo[2,1-*a*]isoindole-2,5(3*H*,9b*H*)-dione (5m)

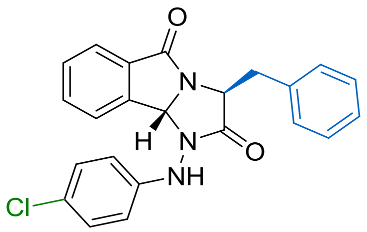

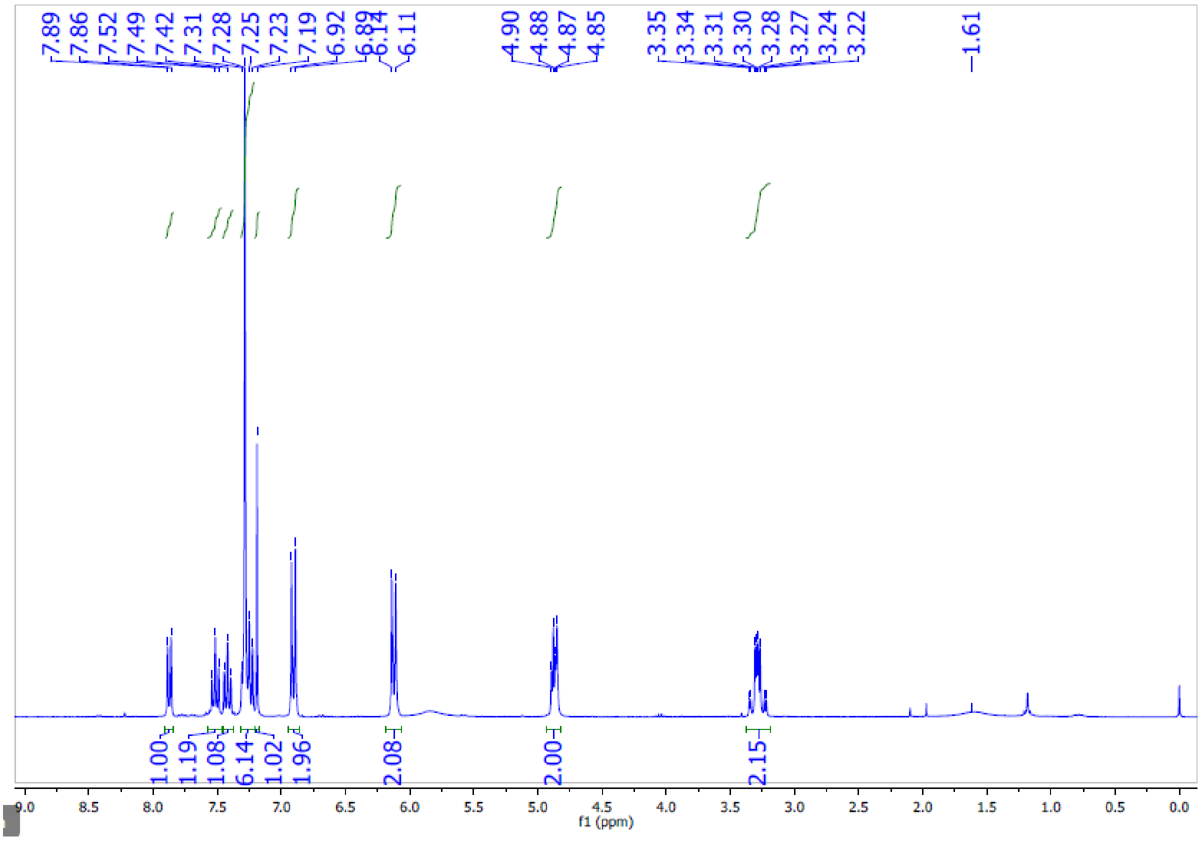

^{1}H NMRspectrum of the compound **5m** in $CDCl_3$ at 300 MHz

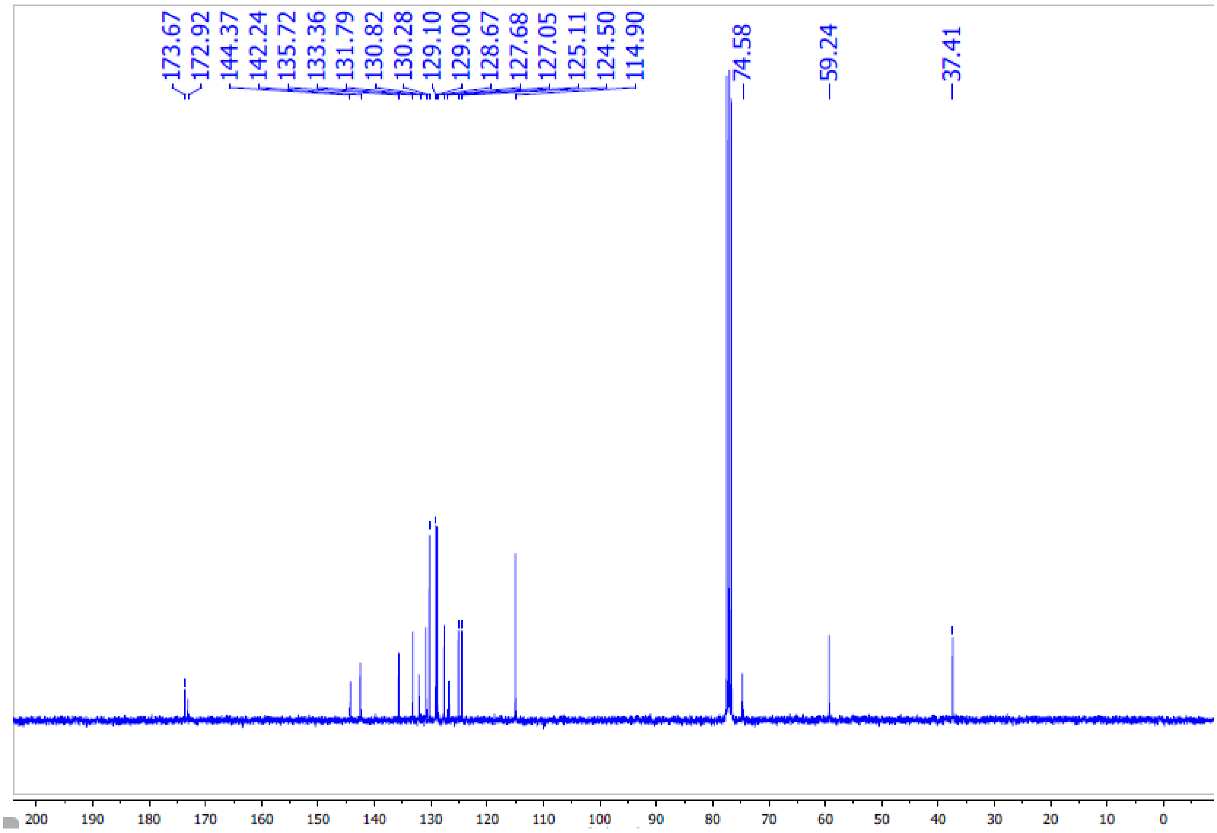

^{13}C NMRspectrum of the compound **5m** in $CDCl_3$ at 75 MHz

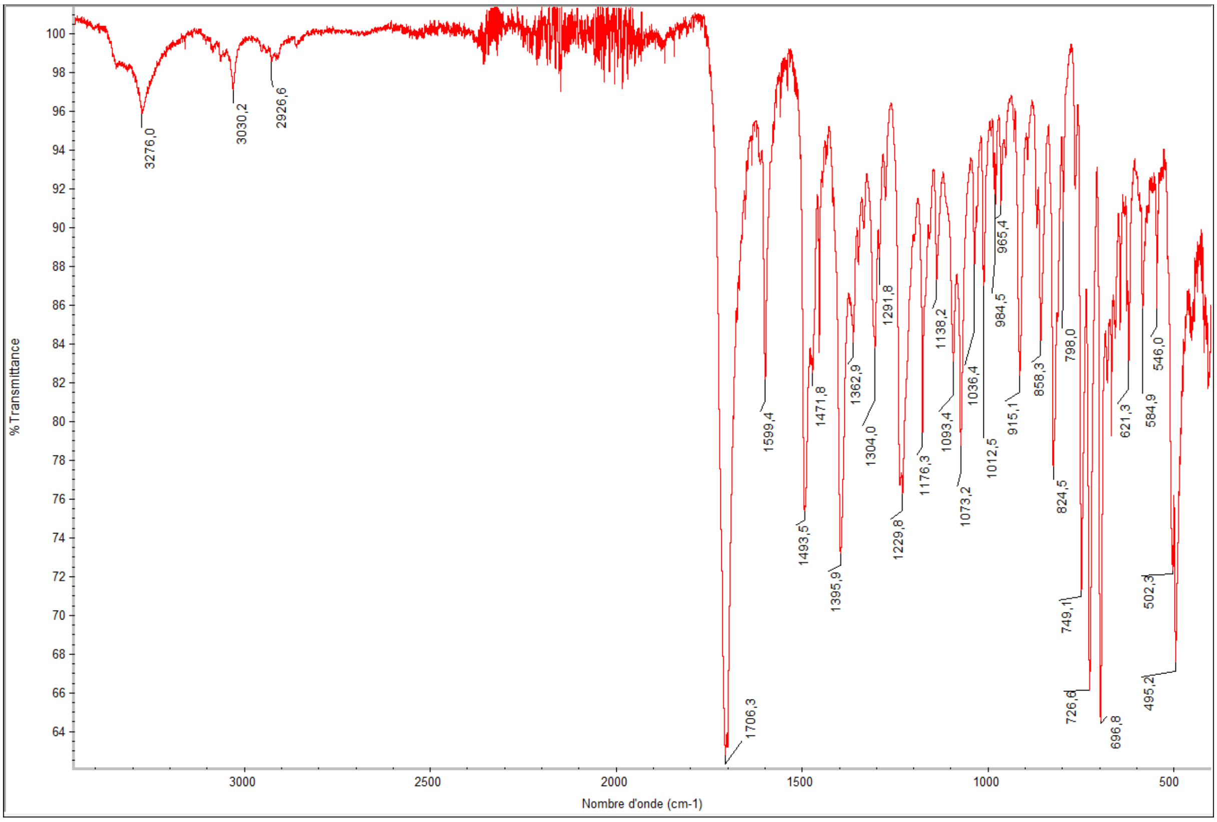

FT-IR spectrum of the compound **5m**
