# Supplementary material for: Green synthesis of new chiral 1-(arylamino)imidazo[2,1-a]isoindole-2,5-diones from the corresponding α-amino acid arylhydrazides in aqueous medium
Source: Beilstein J Org Chem. 2018 Nov 26;14:2923–30. doi: 10.3762/bjoc.14.271 (PMC6278760; doi:10.3762/bjoc.14.271)
Supplement: File 2 — Crystallographic information for compound 5f. [file Beilstein_J_Org_Chem-14-2923-s002.pdf]

BEILSTEIN JOURNAL OF ORGANIC CHEMISTRY

Supporting Information

for

Green synthesis of new chiral 1-(arylamino)imidazo[2,1-*a*]isoindole-2,5-diones from the corresponding α-amino acid arylhydrazides in aqueous medium

Nadia Bouzayani, Jamil Kraïem, Sylvain Marque, Yakdhane Kacem, Abel Carlin-Sinclair, Jérôme Marrot and Béchir Ben Hassine

Beilstein J. Org. Chem. **2018,** *14,* 2923–2930. doi:10.3762/bjoc.14.271

Crystallographic information for compound 5f

License and Terms: This is a supporting information file under the terms of the Creative Commons Attribution License (http://creativecommons.org/licenses/by/4.0). Please note that the reuse, redistribution and reproduction in particular requires that the authors and source are credited.
The license is subject to the *Beilstein Journal of Organic Chemistry* terms and conditions: (https://www.beilstein-journals.org/bjoc)

Sealed tube synthesis of new chiral 1-(phenylamino)imidazo[2,1-*a*] iso-indole-2,5-diones from *α*-amino acid phenylhydrazides in aqueous medium

Nadia Bouzayani,[a] Jamil Kraïem,[b] Sylvain Marque,[c]* Yakdhane Kacem,[a] Jérôme Marrot[c] and Béchir Ben Hassine[a]

[a]Laboratoire de Synthèse Organique Asymétrique et Catalyse Homogène, (UR 11ES56), Université de Monastir, Faculté des Sciences de Monastir, Avenue de l'environnement, 5000 Monastir, Tunisie, [b]Laboratoire de Développement Chimique, Galénique et Pharmacologique des Médicaments, Faculté de Pharmacie de Monastir, Université de Monastir, Rue Avicenne, 5000 Monastir, Tunisie, and [c]Université de Versailles Saint-Quentin en Yvelines, Institut Lavoisier de Versailles, UMR CNRS 8180, 45 Avenue des Etats Unis, 78035 Versailles Cedex, France
Correspondence email: sylvain.marque@uvsq.fr

Abstract

Related literature

Computing details

Data collection: Bruker *APEX2*; cell refinement: Bruker *SAINT*; data reduction: Bruker *SAINT*; program(s) used to solve structure: *SHELXT* 2014/5 (Sheldrick, 2014); program(s) used to refine structure: *SHELXL2018*/1 (Sheldrick, 2018); molecular graphics: Bruker *SHELXTL*; software used to prepare material for publication: Bruker *SHELXTL*.

Acknowledgements

Funding information

References

Figure 1

(sm_af_a)

Crystal data

$C_{19}H_{19}N_3O_2S$
M_r = 353.43
Orthorhombic, $P2_12_12_1$
a = 6.3980 (3) Å
b = 14.3384 (6) Å
c = 19.2989 (9) Å
V = 1770.42 (14) Å^3
Z = 4
F(000) = 744
D_x = 1.326 Mg m^{-3}
Mo $K\alpha$ radiation, λ = 0.71073 Å
Cell parameters from 9923 reflections
θ = 2.5–24.9°
μ = 0.20 mm^{-1}
T = 250 K
Needle, colorless
0.30 × 0.08 × 0.04 mm

Data collection

Bruker D8 VENTURE diffractometer
Radiation source: microsource
φ and ω scans
Absorption correction: multi-scan *SADABS* (Sheldrick, V2016/2)
33260 measured reflections
3136 independent reflections
2939 reflections with $I > 2\sigma(I)$
R_{int} = 0.069
θ_{max} = 25.0°, θ_{min} = 2.5°
h = −7→7
k = −17→17
l = −22→22

Refinement

Refinement on F^2
Least-squares matrix: full
$R[F^2 > 2\sigma(F^2)] = 0.032$
$wR(F^2) = 0.081$
$S = 1.08$
3136 reflections
231 parameters
0 restraints
Hydrogen site location: mixed
H atoms treated by a mixture of independent and constrained refinement
$w = 1/[\sigma^2(F_o^2) + (0.0387P)^2 + 0.3956P]$ where $P = (F_o^2 + 2F_c^2)/3$
$(\Delta/\sigma)_{max} < 0.001$
$\Delta\rho_{max} = 0.15$ e Å^{-3}
$\Delta\rho_{min} = -0.25$ e Å^{-3}
Absolute structure: Flack x determined using 1166 quotients [(I+)-(I-)]/[(I+)+(I-)] (Parsons, Flack and Wagner, Acta Cryst. B69 (2013) 249-259).
Absolute structure parameter: −0.03 (3)

Special details

Geometry. All e.s.d.'s (except the e.s.d. in the dihedral angle between two l.s. planes) are estimated using the full covariance matrix. The cell e.s.d.'s are taken into account individually in the estimation of e.s.d.'s in distances, angles and torsion angles; correlations between e.s.d.'s in cell parameters are only used when they are defined by crystal symmetry. An approximate (isotropic) treatment of cell e.s.d.'s is used for estimating e.s.d.'s involving l.s. planes.

Fractional atomic coordinates and isotropic or equivalent isotropic displacement parameters (Å^2)

	x	y	z	U_{iso}*/U_{eq}
S1	1.01508 (11)	0.28861 (5)	0.70664 (4)	0.0419 (2)
O1	0.4613 (3)	0.27599 (14)	0.51529 (12)	0.0507 (6)
O2	0.8482 (3)	0.52461 (15)	0.65928 (13)	0.0496 (5)
C1	0.3301 (4)	0.47544 (16)	0.61104 (12)	0.0256 (5)
H1	0.214384	0.459434	0.642940	0.031*
N2	0.3283 (3)	0.41647 (14)	0.54921 (10)	0.0272 (5)
C3	0.4622 (4)	0.34335 (17)	0.55369 (13)	0.0313 (6)
C4	0.6071 (4)	0.36106 (17)	0.61501 (14)	0.0298 (6)
H4	0.752780	0.367192	0.598310	0.036*
N5	0.5332 (3)	0.45171 (14)	0.64078 (10)	0.0266 (4)
C6	0.6638 (4)	0.52833 (18)	0.64335 (13)	0.0323 (6)
C7	0.5394 (4)	0.60980 (17)	0.62157 (13)	0.0315 (6)
C8	0.3451 (4)	0.57944 (17)	0.59960 (13)	0.0278 (5)
C9	0.1981 (5)	0.64156 (18)	0.57433 (14)	0.0365 (6)
H9	0.064472	0.621564	0.560608	0.044*
C10	0.2566 (5)	0.73491 (19)	0.57009 (15)	0.0428 (7)
H10	0.161279	0.778574	0.552150	0.051*
C11	0.4515 (6)	0.7650 (2)	0.59164 (15)	0.0462 (8)
H11	0.486216	0.828531	0.588016	0.055*
C12	0.5953 (5)	0.7033 (2)	0.61828 (15)	0.0424 (7)
H12	0.726910	0.723767	0.633728	0.051*
N13	0.1488 (3)	0.41423 (14)	0.50836 (11)	0.0289 (5)
H13	0.098 (4)	0.352 (2)	0.5048 (15)	0.039 (8)*
C14	0.1658 (4)	0.45885 (16)	0.44311 (13)	0.0276 (5)
C15	−0.0020 (5)	0.45050 (17)	0.39811 (14)	0.0352 (6)
H15	−0.120220	0.415946	0.411393	0.042*
C16	0.0040 (6)	0.49278 (19)	0.33378 (15)	0.0434 (7)
H16	−0.109403	0.485927	0.303215	0.052*
C17	0.1752 (5)	0.5450 (2)	0.31411 (14)	0.0429 (7)
H17	0.179847	0.572965	0.270095	0.051*
C18	0.3390 (5)	0.55563 (19)	0.35968 (15)	0.0405 (7)
H18	0.453978	0.592759	0.347052	0.049*

C19	0.3369 (5)	0.51228 (18)	0.42405 (14)	0.0328 (6)
H19	0.450642	0.519134	0.454450	0.039*
C20	0.5949 (4)	0.2837 (2)	0.66838 (15)	0.0366 (6)
H20A	0.628213	0.224182	0.646043	0.044*
H20B	0.451634	0.279796	0.686085	0.044*
C21	0.7439 (4)	0.2991 (2)	0.72877 (14)	0.0393 (6)
H21A	0.711367	0.253777	0.765201	0.047*
H21B	0.719468	0.361530	0.747843	0.047*
C22	1.0336 (6)	0.1653 (2)	0.6965 (2)	0.0708 (11)
H22A	0.968197	0.134734	0.735799	0.106*
H22B	1.179514	0.147241	0.694056	0.106*
H22C	0.963049	0.146701	0.654223	0.106*

Atomic displacement parameters (Å²)

	U^{11}	U^{22}	U^{33}	U^{12}	U^{13}	U^{23}
S1	0.0288 (3)	0.0342 (3)	0.0627 (5)	0.0008 (3)	−0.0109 (3)	−0.0049 (3)
O1	0.0524 (13)	0.0366 (11)	0.0630 (13)	0.0160 (10)	−0.0176 (11)	−0.0229 (10)
O2	0.0294 (11)	0.0446 (12)	0.0748 (15)	−0.0055 (9)	−0.0085 (11)	−0.0111 (11)
C1	0.0253 (12)	0.0236 (12)	0.0277 (12)	0.0004 (10)	−0.0002 (10)	0.0004 (10)
N2	0.0285 (11)	0.0248 (11)	0.0284 (11)	0.0017 (9)	−0.0054 (9)	−0.0028 (9)
C3	0.0280 (14)	0.0268 (13)	0.0391 (14)	0.0021 (11)	−0.0024 (12)	−0.0041 (11)
C4	0.0241 (13)	0.0263 (13)	0.0391 (15)	0.0034 (10)	0.0000 (11)	−0.0026 (11)
N5	0.0241 (10)	0.0247 (10)	0.0311 (11)	−0.0006 (9)	−0.0017 (9)	−0.0006 (8)
C6	0.0303 (14)	0.0309 (13)	0.0356 (14)	−0.0042 (11)	0.0016 (12)	−0.0075 (11)
C7	0.0398 (16)	0.0257 (12)	0.0291 (13)	−0.0034 (11)	0.0058 (12)	−0.0058 (10)
C8	0.0334 (14)	0.0237 (12)	0.0263 (12)	0.0013 (11)	0.0057 (11)	−0.0049 (10)
C9	0.0446 (17)	0.0299 (14)	0.0349 (14)	0.0056 (13)	−0.0017 (12)	−0.0032 (11)
C10	0.064 (2)	0.0263 (14)	0.0383 (15)	0.0100 (14)	−0.0008 (15)	−0.0011 (12)
C11	0.072 (2)	0.0242 (14)	0.0424 (16)	−0.0049 (14)	0.0084 (16)	−0.0042 (11)
C12	0.0481 (17)	0.0319 (15)	0.0473 (17)	−0.0089 (13)	0.0056 (14)	−0.0081 (13)
N13	0.0292 (12)	0.0242 (11)	0.0332 (12)	−0.0039 (10)	−0.0066 (10)	0.0002 (9)
C14	0.0349 (13)	0.0173 (11)	0.0308 (12)	0.0047 (11)	−0.0027 (11)	−0.0032 (10)
C15	0.0382 (15)	0.0254 (12)	0.0419 (14)	−0.0028 (13)	−0.0088 (13)	−0.0007 (11)
C16	0.0573 (19)	0.0315 (14)	0.0414 (15)	−0.0010 (14)	−0.0197 (15)	0.0016 (11)
C17	0.065 (2)	0.0323 (14)	0.0313 (14)	0.0017 (15)	−0.0027 (14)	0.0037 (11)
C18	0.0483 (17)	0.0335 (14)	0.0397 (16)	−0.0017 (13)	0.0077 (14)	0.0043 (12)
C19	0.0334 (14)	0.0305 (13)	0.0347 (13)	0.0003 (12)	−0.0021 (12)	−0.0024 (11)
C20	0.0241 (13)	0.0323 (14)	0.0535 (17)	−0.0002 (11)	−0.0015 (11)	0.0079 (13)
C21	0.0390 (15)	0.0417 (15)	0.0373 (14)	0.0105 (13)	0.0021 (12)	−0.0001 (13)
C22	0.049 (2)	0.0397 (18)	0.124 (3)	0.0102 (17)	−0.003 (2)	−0.011 (2)

Geometric parameters (Å, º)

S1—C22	1.783 (4)	C11—H11	0.9400
S1—C21	1.793 (3)	C12—H12	0.9400
O1—C3	1.217 (3)	N13—C14	1.417 (3)
O2—C6	1.220 (4)	N13—H13	0.95 (3)
C1—N5	1.460 (3)	C14—C15	1.386 (4)
C1—N2	1.463 (3)	C14—C19	1.386 (4)
C1—C8	1.511 (3)	C15—C16	1.382 (4)
C1—H1	0.9900	C15—H15	0.9400

N2—C3	1.357 (3)	C16—C17	1.380 (5)
N2—N13	1.393 (3)	C16—H16	0.9400
C3—C4	1.525 (4)	C17—C18	1.377 (5)
C4—N5	1.470 (3)	C17—H17	0.9400
C4—C20	1.515 (4)	C18—C19	1.389 (4)
C4—H4	0.9900	C18—H18	0.9400
N5—C6	1.381 (3)	C19—H19	0.9400
C6—C7	1.475 (4)	C20—C21	1.522 (4)
C7—C8	1.383 (4)	C20—H20A	0.9800
C7—C12	1.389 (4)	C20—H20B	0.9800
C8—C9	1.384 (4)	C21—H21A	0.9800
C9—C10	1.392 (4)	C21—H21B	0.9800
C9—H9	0.9400	C22—H22A	0.9700
C10—C11	1.384 (5)	C22—H22B	0.9700
C10—H10	0.9400	C22—H22C	0.9700
C11—C12	1.376 (5)		

C22—S1—C21	100.00 (17)	C11—C12—H12	121.1
N5—C1—N2	101.14 (19)	C7—C12—H12	121.1
N5—C1—C8	103.4 (2)	N2—N13—C14	115.4 (2)
N2—C1—C8	116.9 (2)	N2—N13—H13	109.9 (17)
N5—C1—H1	111.5	C14—N13—H13	112.7 (18)
N2—C1—H1	111.5	C15—C14—C19	119.6 (2)
C8—C1—H1	111.5	C15—C14—N13	117.3 (2)
C3—N2—N13	122.60 (19)	C19—C14—N13	123.1 (2)
C3—N2—C1	112.9 (2)	C16—C15—C14	120.2 (3)
N13—N2—C1	118.8 (2)	C16—C15—H15	119.9
O1—C3—N2	124.8 (2)	C14—C15—H15	119.9
O1—C3—C4	127.4 (2)	C17—C16—C15	120.4 (3)
N2—C3—C4	107.7 (2)	C17—C16—H16	119.8
N5—C4—C20	113.6 (2)	C15—C16—H16	119.8
N5—C4—C3	102.38 (19)	C18—C17—C16	119.3 (3)
C20—C4—C3	112.0 (2)	C18—C17—H17	120.4
N5—C4—H4	109.5	C16—C17—H17	120.4
C20—C4—H4	109.5	C17—C18—C19	120.9 (3)
C3—C4—H4	109.5	C17—C18—H18	119.5
C6—N5—C1	111.5 (2)	C19—C18—H18	119.5
C6—N5—C4	121.4 (2)	C14—C19—C18	119.5 (3)
C1—N5—C4	111.06 (19)	C14—C19—H19	120.2
O2—C6—N5	124.0 (3)	C18—C19—H19	120.2
O2—C6—C7	128.9 (3)	C4—C20—C21	112.5 (2)
N5—C6—C7	107.0 (2)	C4—C20—H20A	109.1
C8—C7—C12	121.4 (3)	C21—C20—H20A	109.1
C8—C7—C6	108.9 (2)	C4—C20—H20B	109.1
C12—C7—C6	129.7 (3)	C21—C20—H20B	109.1
C7—C8—C9	121.1 (2)	H20A—C20—H20B	107.8
C7—C8—C1	108.8 (2)	C20—C21—S1	114.3 (2)
C9—C8—C1	130.1 (3)	C20—C21—H21A	108.7
C8—C9—C10	117.2 (3)	S1—C21—H21A	108.7
C8—C9—H9	121.4	C20—C21—H21B	108.7
C10—C9—H9	121.4	S1—C21—H21B	108.7
C11—C10—C9	121.6 (3)	H21A—C21—H21B	107.6

C11—C10—H10	119.2	S1—C22—H22A	109.5
C9—C10—H10	119.2	S1—C22—H22B	109.5
C12—C11—C10	121.0 (3)	H22A—C22—H22B	109.5
C12—C11—H11	119.5	S1—C22—H22C	109.5
C10—C11—H11	119.5	H22A—C22—H22C	109.5
C11—C12—C7	117.7 (3)	H22B—C22—H22C	109.5

Hydrogen-bond geometry (Å, º)

D—H···*A*	*D*—H	H···*A*	*D*···*A*	*D*—H···*A*
C1—H1···S1^{i}	0.99	3.02	3.827 (3)	139
C1—H1···O2^{i}	0.99	2.54	3.297 (3)	133
N13—H13···O1^{ii}	0.95 (3)	2.07 (3)	3.014 (3)	171 (3)
C20—H20*B*···S1^{i}	0.98	2.82	3.783 (3)	166

Symmetry codes: (i) *x*−1, *y*, *z*; (ii) *x*−1/2, −*y*+1/2, −*z*+1.
