# Supplementary material for: Green synthesis of new chiral 1-(arylamino)imidazo[2,1-a]isoindole-2,5-diones from the corresponding α-amino acid arylhydrazides in aqueous medium
Source: Beilstein J Org Chem. 2018 Nov 26;14:2923–30. doi: 10.3762/bjoc.14.271 (PMC6278760; doi:10.3762/bjoc.14.271)
Supplement: File 3 — HPLC analysis of the products. [file Beilstein_J_Org_Chem-14-2923-s003.pdf]

BEILSTEIN JOURNAL OF ORGANIC CHEMISTRY

Supporting Information

for

Green synthesis of new chiral 1-(arylamino)imidazo[2,1-*a*]isoindole-2,5-diones from the corresponding α-amino acid arylhydrazides in aqueous medium

Nadia Bouzayani, Jamil Kraïem, Sylvain Marque, Yakdhane Kacem, Abel Carlin-Sinclair, Jérôme Marrot and Béchir Ben Hassine

Beilstein J. Org. Chem. **2018,** *14,* 2923–2930. doi:10.3762/bjoc.14.271

HPLC analysis of the products

License and Terms: This is a supporting information file under the terms of the Creative Commons Attribution License (http://creativecommons.org/licenses/by/4.0). Please note that the reuse, redistribution and reproduction in particular requires that the authors and source are credited.
The license is subject to the *Beilstein Journal of Organic Chemistry* terms and conditions: (https://www.beilstein-journals.org/bjoc)

Table of contents

1. HPLC analysis of **5a**……………………………………………………………………..S2

2. HPLC analysis of **5b**……………………………………………………………………..S3

3. HPLC analysis of **5c**……………………………………………………………………..S4

4. HPLC analysis of **5d**……………………………………………………………………..S5

5. HPLC analysis of **5e**……………………………………………………………………..S6

6. HPLC analysis of **5f**……………………………………………………………………..S7

7. HPLC analysis of **5g**……………………………………………………………………..S8

8. HPLC analysis of **5h**……………………………………………………………………..S9

9. HPLC analysis of **5i**……………………………………………………………………..S10

10. HPLC analysis of **5j**…………………………………………………………………S11

11. Tables of retention times and ratios……………………………………………..……..S12

1. HPLC analysis of **5a**

High performance liquid chromatography (HPLC) was performed on a Welk RR column (4.6 × 250 mm) (REGIS Technologies) using a mobile phase: heptane / iPrOH / CH_2Cl_2 (50/40/10) with a flow rate of 1.0 mL / min at 25 °C.

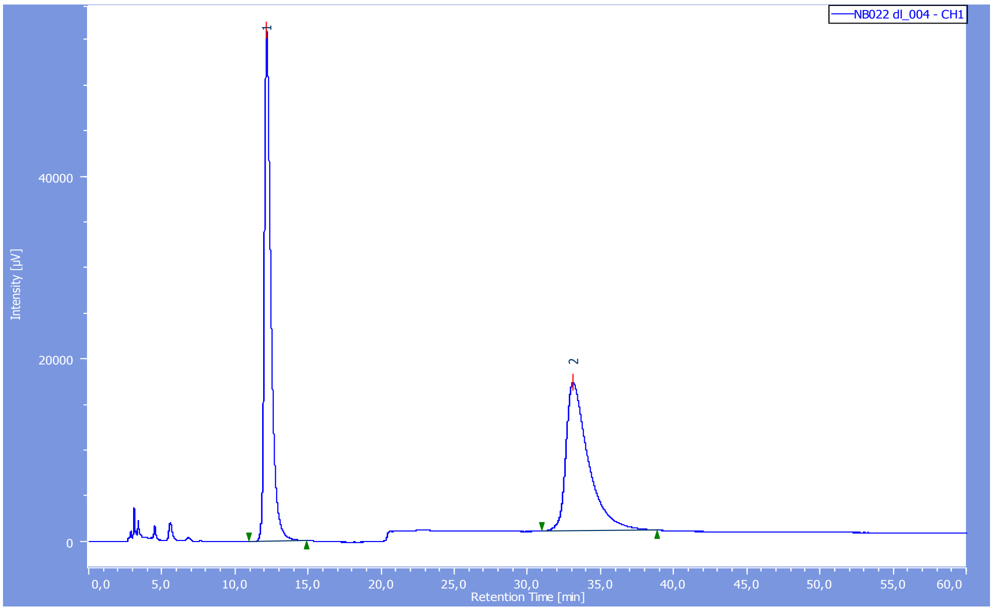

Chromatogram of the compound **5a** starting with the racemic phenylhydrazide **3a**

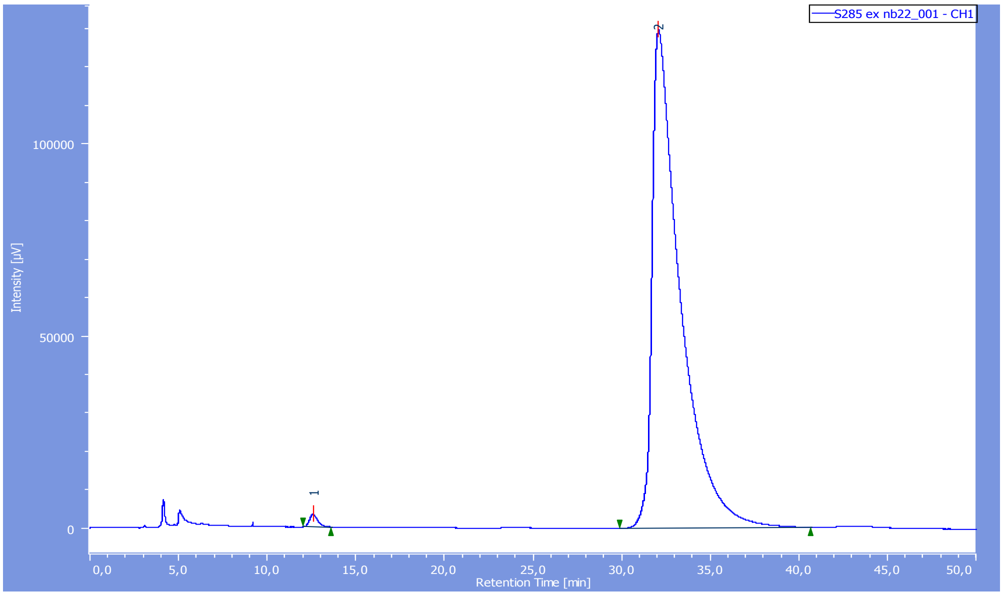

Chromatogram of the compound **5a** starting with (L)-phenylhydrazide **3a**

2. HPLC analysis of **5b**

High performance liquid chromatography (HPLC) was performed on a Welk RR column (4.6 × 250 mm) (REGIS Technologies) using a mobile phase: heptane / iPrOH / CH_2Cl_2 (50/40/10) with a flow rate of 1.0 mL / min at 25 °C.

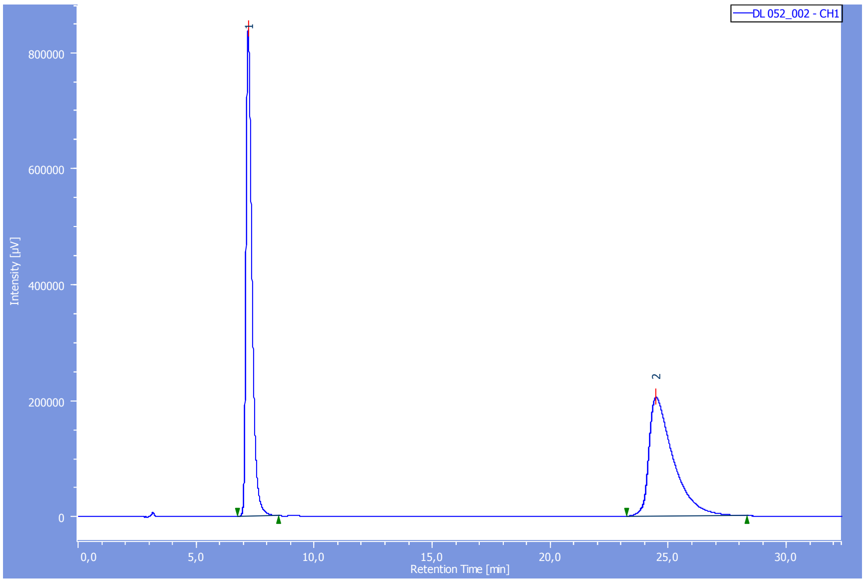

Chromatogram of the compound **5b** starting with the racemic phenylhydrazide **3b**

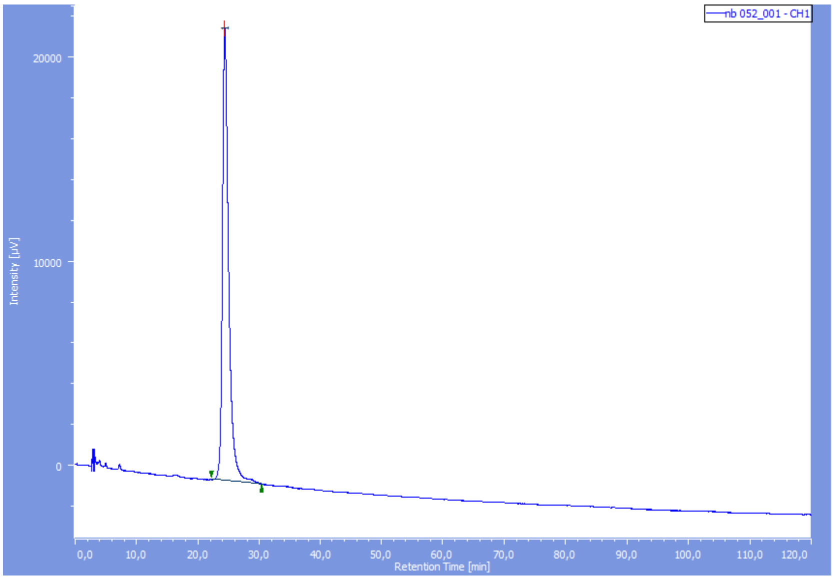

Chromatogram of the compound **5b** starting with (L)-phenylhydrazide **3b**

3. HPLC analysis of **5c**

High performance liquid chromatography (HPLC) was performed on a Welk RR column (4.6 × 250 mm) (REGIS Technologies) using a mobile phase: heptane / iPrOH (30/70) with a flow rate of 1.0 mL / min at 25 °C.

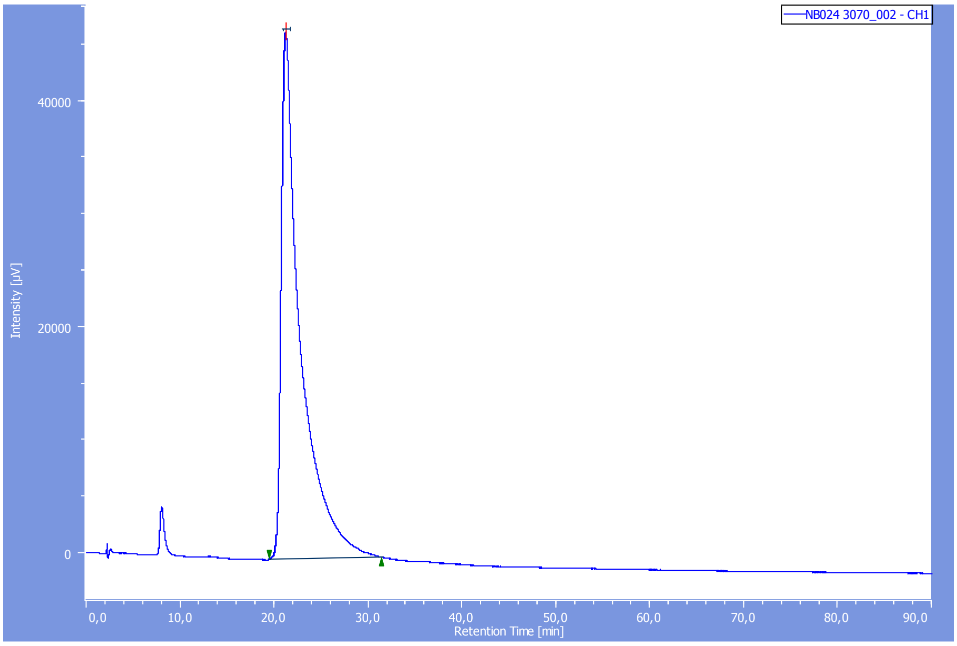

Chromatogram of the compound **5c** starting with (L)-phenylhydrazide **3c**

4. HPLC analysis of **5d**

High performance liquid chromatography (HPLC) was performed on a Welk RR column (4.6 × 250 mm) (REGIS Technologies) using a mobile phase: heptane / iPrOH / CH_2Cl_2 (50/40/10) with a flow rate of 1.0 mL / min at 25 °C.

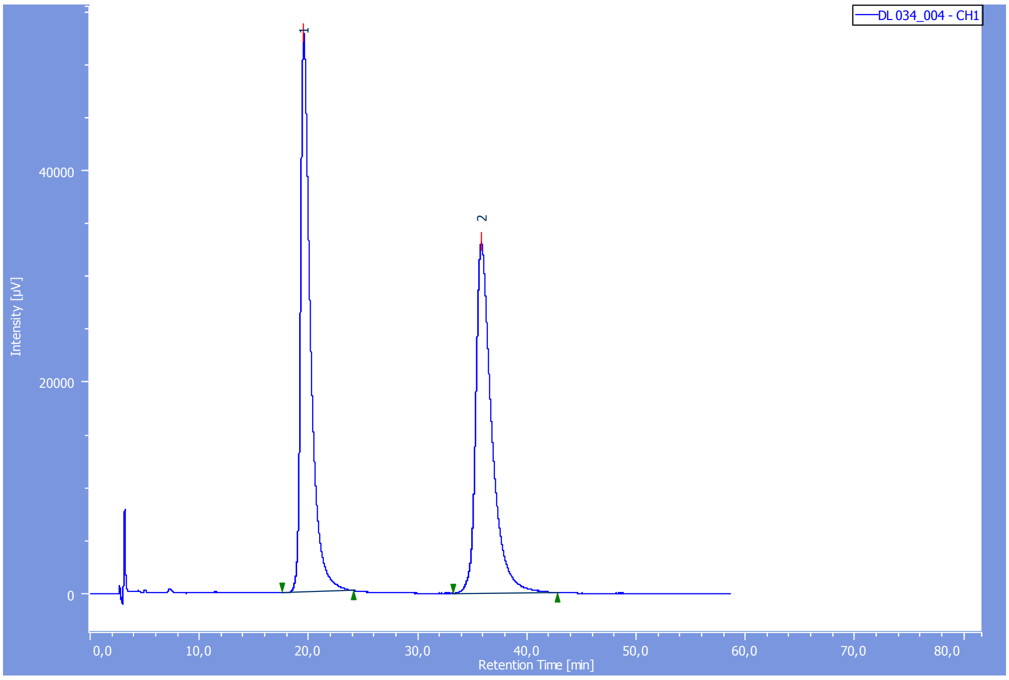

Chromatogram of the compound **5d** starting with the racemic phenylhydrazide **3d**

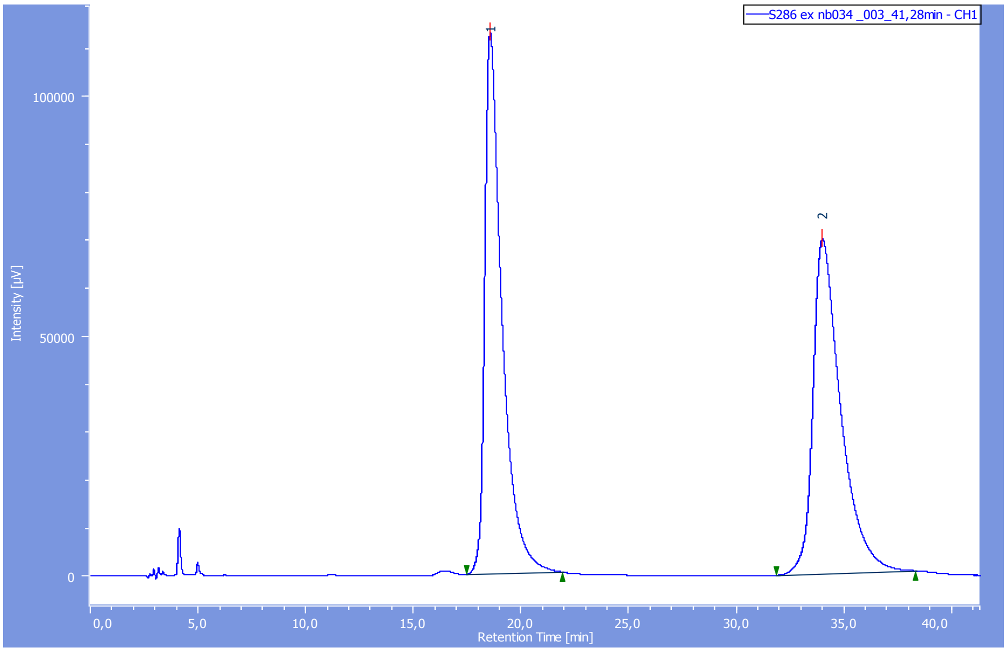

Chromatogram of the compound **5d** starting with (L)-phenylhydrazide **3d**

5. HPLC analysis of **5e**

High performance liquid chromatography (HPLC) was performed on a Welk RR column (4.6 × 250 mm) (REGIS Technologies) using a mobile phase: heptane / iPrOH / CH_2Cl_2 (50/40/10) with a flow rate of 1.0 mL / min at 25 °C.

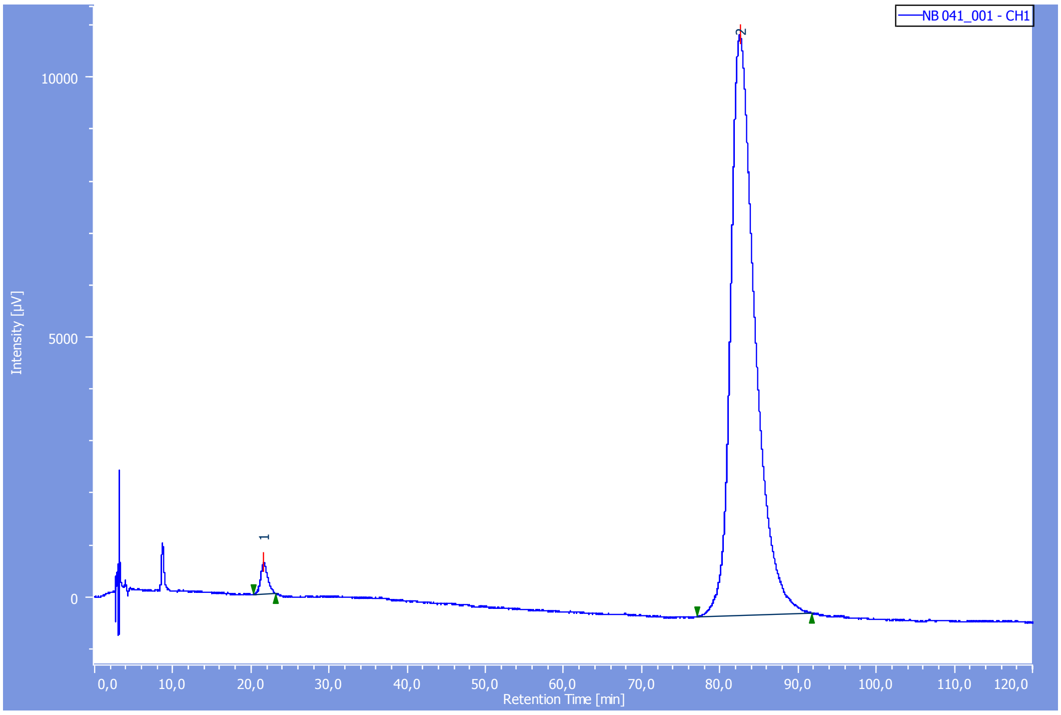

Chromatogram of the compound **5e** starting with (L)-phenylhydrazide **3e**

6. HPLC analysis of **5f**

High performance liquid chromatography (HPLC) was performed on a Welk RR column (4.6 × 250 mm) (REGIS Technologies) using a mobile phase: heptane / iPrOH / CH_2Cl_2 (50/40/10) with a flow rate of 1.0 mL / min at 25 °C.

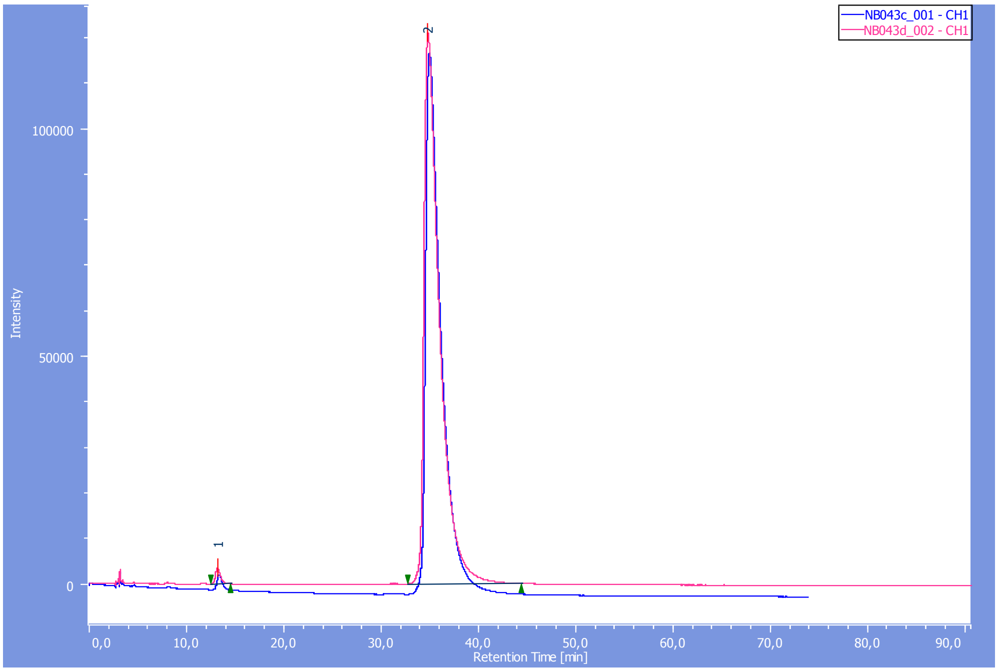

Chromatogram of the compound **5f** starting with (L)-phenylhydrazide **3f**

7. HPLC analysis of **5g**

High performance liquid chromatography (HPLC) was performed on a Welk RR column (4.6 × 250 mm) (REGIS Technologies) using a mobile phase: heptane / iPrOH / CH_2Cl_2 (50/40/10) with a flow rate of 1.0 mL / min at 25 °C.

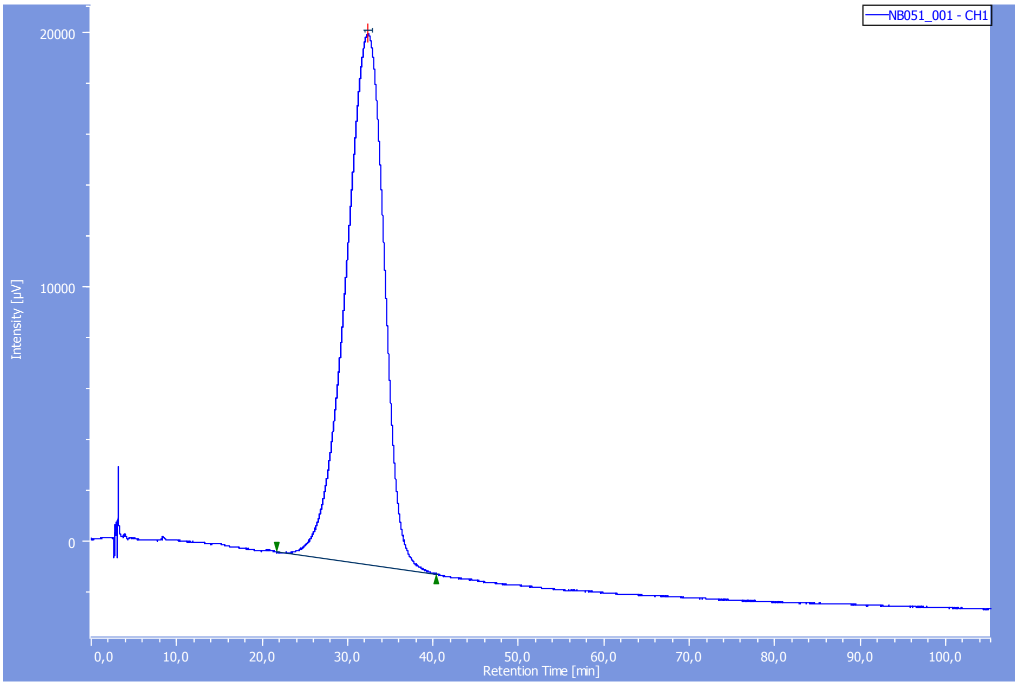

Chromatogram of the compound **5g** starting with (L)-phenylhydrazide **3g**

8. HPLC analysis of **5h**

High performance liquid chromatography (HPLC) was performed on a Welk RR column (4.6 × 250 mm) (REGIS Technologies) using a mobile phase: heptane / iPrOH / CH_2Cl_2 (50/40/10) with a flow rate of 1.0 mL / min at 25 °C.

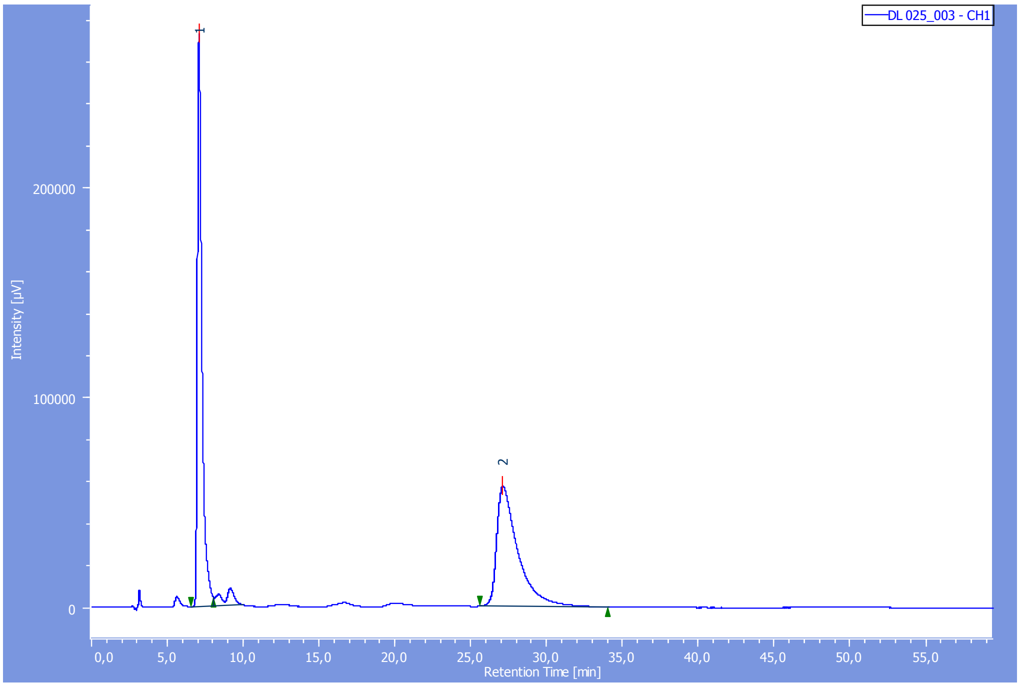

Chromatogram of the compound **5h** starting with the racemic phenylhydrazide **3h**

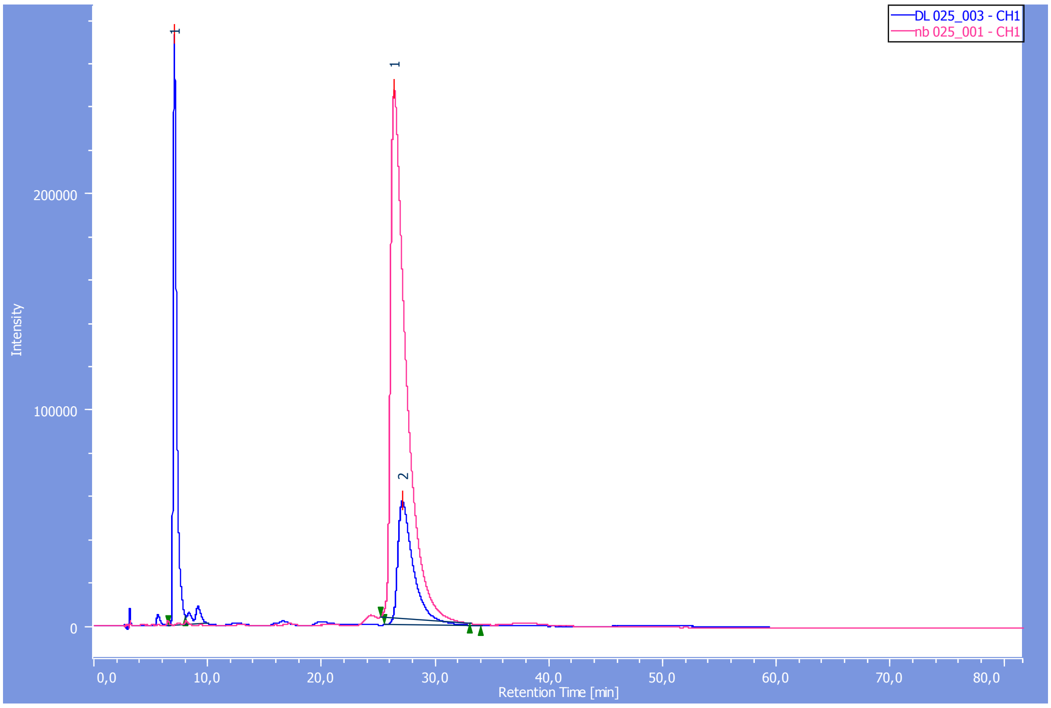

Chromatogram of the compound **5h** starting with (L)-phenylhydrazide **3h**

9. HPLC analysis of **5i**

High performance liquid chromatography (HPLC) was performed on a Welk RR column (4.6 × 250 mm) (REGIS Technologies) using a mobile phase: heptane / iPrOH / CH_2Cl_2 (50/40/10) with a flow rate of 1.0 mL / min at 25 °C.

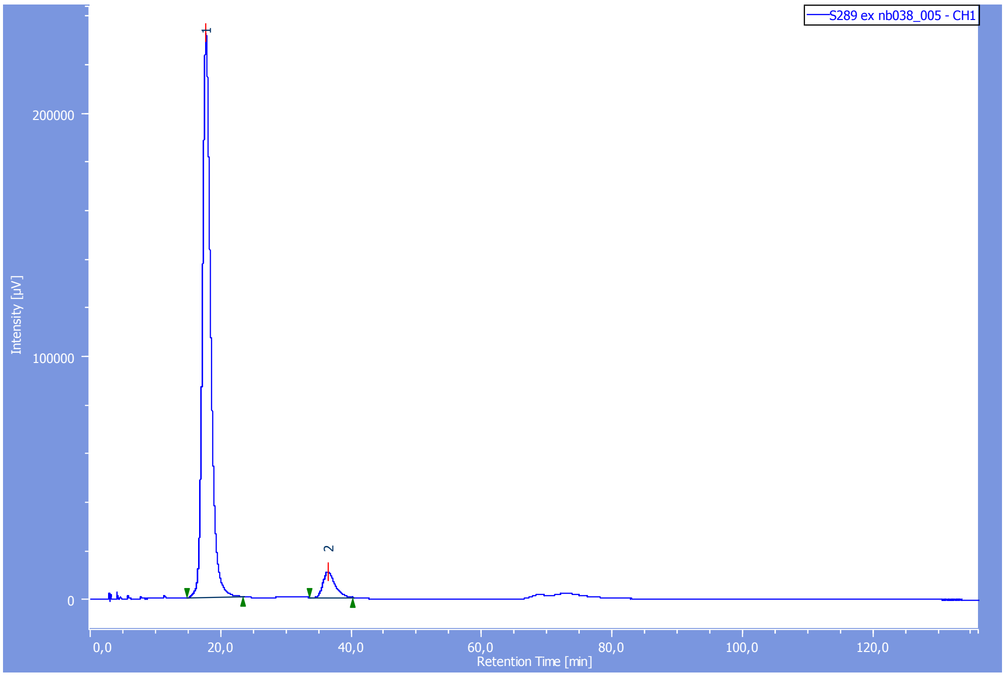

Chromatogram of the compound **5i** in the racemic form and starting with (L)-phenylhydrazide **3i**

10. HPLC analysis of **5j**

High performance liquid chromatography (HPLC) was performed on a Welk RR column (4.6 × 250 mm) (REGIS Technologies) using a mobile phase: heptane / iPrOH / CH_2Cl_2 (50/40/10) with a flow rate of 1.0 mL / min at 25 °C.

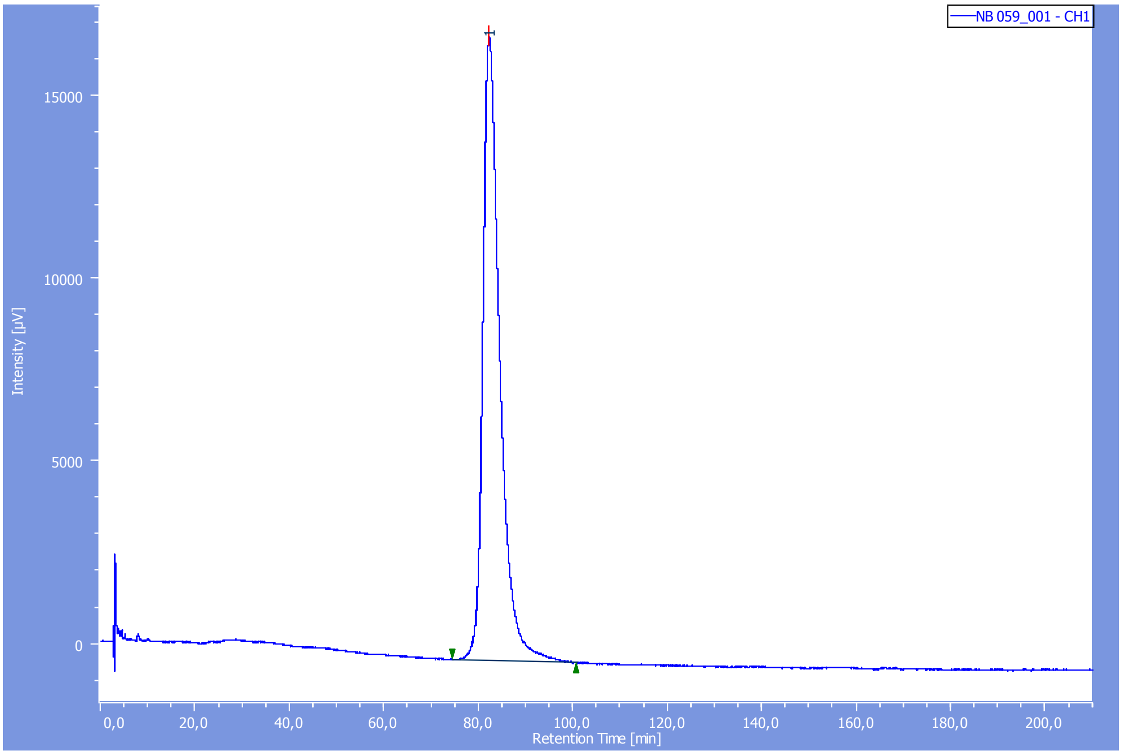

Chromatogram of the compound **5j** starting with (L)-phenylhydrazide **3j**

11. Tables of retention times and ratios

**Table 1:** HPLC analysis of the compounds **5** starting with (L)-arylhydrazide **3**					
**Compound**	**Retention times (min)**	**Area**	**Height**	**Area (%)**	**Height (%)**
**5a**	t_r(1) = 12.56<br>t_r(2) = 33.04	104274<br>14372948	3230<br>129432	0.72<br>99.28	2.435<br>97.565
**5b**	24.32	1549623	22079	100	100
**5c**	21.222	6602367	46520	100	100
**5d**	t_r(1) = 18.55<br>t_r(2) = 33.96	6289878<br>6255211	112666<br>69641	50.13<br>49.86	60.8<br>38.2
**5e**	t_r(1) = 21.612<br>t_r(2) = 82.445	36782<br>2417900	598<br>11143	1.498<br>98.502	5.093<br>94.907
**5f**	t_r(1) = 13.172<br>t_r(2) = 34.792	121372<br>13384956	3446<br>120671	0.899<br>99.101	2.777<br>97.223
**5g**	30.22	3073988	9823	100	100
**5h**	26.382	23202810	243831	100	100
**5i**	t_r(1) = 17.738<br>t_r(2) = 36.418	20256023<br>1354870	231702<br>10584	93.731<br>6.269	95.632<br>4.368
**5j**	82.245	4280786	17038	100	100

**Table 2:** HPLC analysis of the compounds **5** starting with the racemic arylhydrazides **3**					
**Compound**	**Retention times (min)**	**Area**	**Height**	**Area (%)**	**Height (%)**
**5a**	t_r(1) = 12.158<br>t_r(2) = 33.083	1759890<br>1731411	55831<br>16224	50.408<br>49.592	77.484<br>22.516
**5b**	t_r(1) = 7.2<br>t_r(2) = 24.46	14584432<br>14619815	837955<br>204368	49.939<br>50.061	80.393<br>19.607
**5d**	t_r(1) = 19.553<br>t_r(2) = 35.792	2701250<br>2711097	39036<br>32914	49.926<br>50.074	61.427<br>38.573
**5h**	t_r(1) = 7.075<br>t_r(2) = 27.085	5697073<br>5544905	272298<br>57144	50.677<br>49.323	82.654<br>17.346
